# Erratum: Genetic loci associated with heart rate variability and their effects on cardiac disease risk

**DOI:** 10.1038/ncomms16140

**Published:** 2017-08-02

**Authors:** Ilja M. Nolte, M. Loretto Munoz, Vinicius Tragante, Azmeraw T. Amare, Rick Jansen, Ahmad Vaez, Benedikt von der Heyde, Christy L. Avery, Joshua C. Bis, Bram Dierckx, Jenny van Dongen, Stephanie M. Gogarten, Philippe Goyette, Jussi Hernesniemi, Ville Huikari, Shih-Jen Hwang, Deepali Jaju, Kathleen F. Kerr, Alexander Kluttig, Bouwe P. Krijthe, Jitender Kumar, Sander W. van der Laan, Leo-Pekka Lyytikäinen, Adam X. Maihofer, Arpi Minassian, Peter J. van der Most, Martina Müller-Nurasyid, Michel Nivard, Erika Salvi, James D. Stewart, Julian F. Thayer, Niek Verweij, Andrew Wong, Delilah Zabaneh, Mohammad H. Zafarmand, Abdel Abdellaoui, Sulayma Albarwani, Christine Albert, Alvaro Alonso, Foram Ashar, Juha Auvinen, Tomas Axelsson, Dewleen G. Baker, Paul I. W. de Bakker, Matteo Barcella, Riad Bayoumi, Rob J. Bieringa, Dorret Boomsma, Gabrielle Boucher, Annie R. Britton, Ingrid Christophersen, Andrea Dietrich, George B. Ehret, Patrick T. Ellinor, Markku Eskola, Janine F. Felix, John S. Floras, Oscar H. Franco, Peter Friberg, Maaike G. J. Gademan, Mark A. Geyer, Vilmantas Giedraitis, Catharina A. Hartman, Daiane Hemerich, Albert Hofman, Jouke-Jan Hottenga, Heikki Huikuri, Nina Hutri-Kähönen, Xavier Jouven, Juhani Junttila, Markus Juonala, Antti M. Kiviniemi, Jan A. Kors, Meena Kumari, Tatiana Kuznetsova, Cathy C. Laurie, Joop D. Lefrandt, Yong Li, Yun Li, Duanping Liao, Marian C. Limacher, Henry J. Lin, Cecilia M. Lindgren, Steven A. Lubitz, Anubha Mahajan, Barbara McKnight, Henriette Meyer zu Schwabedissen, Yuri Milaneschi, Nina Mononen, Andrew P. Morris, Mike A. Nalls, Gerjan Navis, Melanie Neijts, Kjell Nikus, Kari E. North, Daniel T. O'Connor, Johan Ormel, Siegfried Perz, Annette Peters, Bruce M. Psaty, Olli T. Raitakari, Victoria B. Risbrough, Moritz F. Sinner, David Siscovick, Johannes H. Smit, Nicholas L. Smith, Elsayed Z. Soliman, Nona Sotoodehnia, Jan A. Staessen, Phyllis K. Stein, Adrienne M. Stilp, Katarzyna Stolarz-Skrzypek, Konstantin Strauch, Johan Sundström, Cees A. Swenne, Ann-Christine Syvänen, Jean-Claude Tardif, Kent D. Taylor, Alexander Teumer, Timothy A. Thornton, Lesley E. Tinker, André G. Uitterlinden, Jessica van Setten, Andreas Voss, Melanie Waldenberger, Kirk C. Wilhelmsen, Gonneke Willemsen, Quenna Wong, Zhu-Ming Zhang, Alan B Zonderman, Daniele Cusi, Michele K. Evans, Halina K. Greiser, Pim van der Harst, Mohammad Hassan, Erik Ingelsson, Marjo-Riitta Järvelin, Stefan Kääb, Mika Kähönen, Mika Kivimaki, Charles Kooperberg, Diana Kuh, Terho Lehtimäki, Lars Lind, Caroline M. Nievergelt, Chris J. O'Donnell, Albertine J. Oldehinkel, Brenda Penninx, Alexander P. Reiner, Harriëtte Riese, Arie M. van Roon, John D. Rioux, Jerome I. Rotter, Tamar Sofer, Bruno H. Stricker, Henning Tiemeier, Tanja G. M. Vrijkotte, Folkert W. Asselbergs, Bianca J. J.M Brundel, Susan R. Heckbert, Eric A. Whitsel, Marcel den Hoed, Harold Snieder, Eco J. C. de Geus

Nature Communications 8: Article number: 15805; DOI: 10.1038/ncomms15805 (2017); Published: 06 14 2017; Updated: 08 02 2017

In Supplementary Fig. 10 of this Article, images for panels a and b were inadvertently omitted. The correct version of Supplementary Fig. 10 is provided as Supplementary Information associated with this Erratum. Furthermore, the affiliation details for Azmeraw T. Amare, Benedikt von der Heyde, and Marcel den Hoed are incorrect in this Article. The correct affiliation details for these authors are given below.

Azmeraw T. Amare:

Department of Epidemiology, University of Groningen, University Medical Center Groningen, PO Box 30001, Groningen 9700 RB, The Netherlands.

School of Medicine, University of Adelaide, Adelaide, South Australia, SA 5005, Australia

College of Medicine and Health Sciences, Bahir Dar University, Bahir Dar 6000, Ethiopia.

Benedikt von der Heyde:

Department of Immunology, Genetics and Pathology, Medical Genetics and Genomics, Uppsala University, Uppsala 75327, Sweden.

Science for Life Laboratory, Uppsala University, Uppsala 75237, Sweden.

Marcel den Hoed:

Department of Immunology, Genetics and Pathology, Medical Genetics and Genomics, Uppsala University, Uppsala 75327, Sweden.

Science for Life Laboratory, Uppsala University, Uppsala 75237, Sweden.

## Supplementary Material

Supplementary Information

