# Supplementary material for: Genetic loci associated with heart rate variability and their effects on cardiac disease risk
Source: Nat Commun. 2017 Jun 14;8:15805. doi: 10.1038/ncomms15805 (PMC5474732; doi:10.1038/ncomms15805)
Supplement: Supplementary Information [file ncomms15805-s1.pdf]

SI GUIDE

Type of file: pdf
Size of file: 4,288 KB
Title of file for HTML: Supplementary Information
Description: Supplementary Figures, Supplementary Tables, Supplementary Notes and Supplementary references.

Supplementary Data

Type of file: XLSX
Size of file: 37 KB
Title of file for HTML: Supplementary Data 1
Description: Stage 1, stage 2, and combined stage 1+2 meta-analysis results for SDNN, RMSSD, and pvRSA/HF for the 23 SNPs that were genome-wide suggestive (p<1x10-6) after stage 1 in the meta-analysis for any of the traits.

Type of file: XLSX
Size of file: 63 KB
Title of file for HTML: Supplementary Data 2
Description: In silico annotation and GWAS catalog lookup results of the 17 HRV SNPs identified in this study.

Type of file: XLSX
Size of file: 58 KB
Title of file for HTML: Supplementary Data 3
Description: DEPICT tissue enrichment analysis.
Type of file: pdf
Size of file: 1,017 KB
Title of file for HTML: Peer Review File
Description:

SUPPLEMENTARY FIGURES

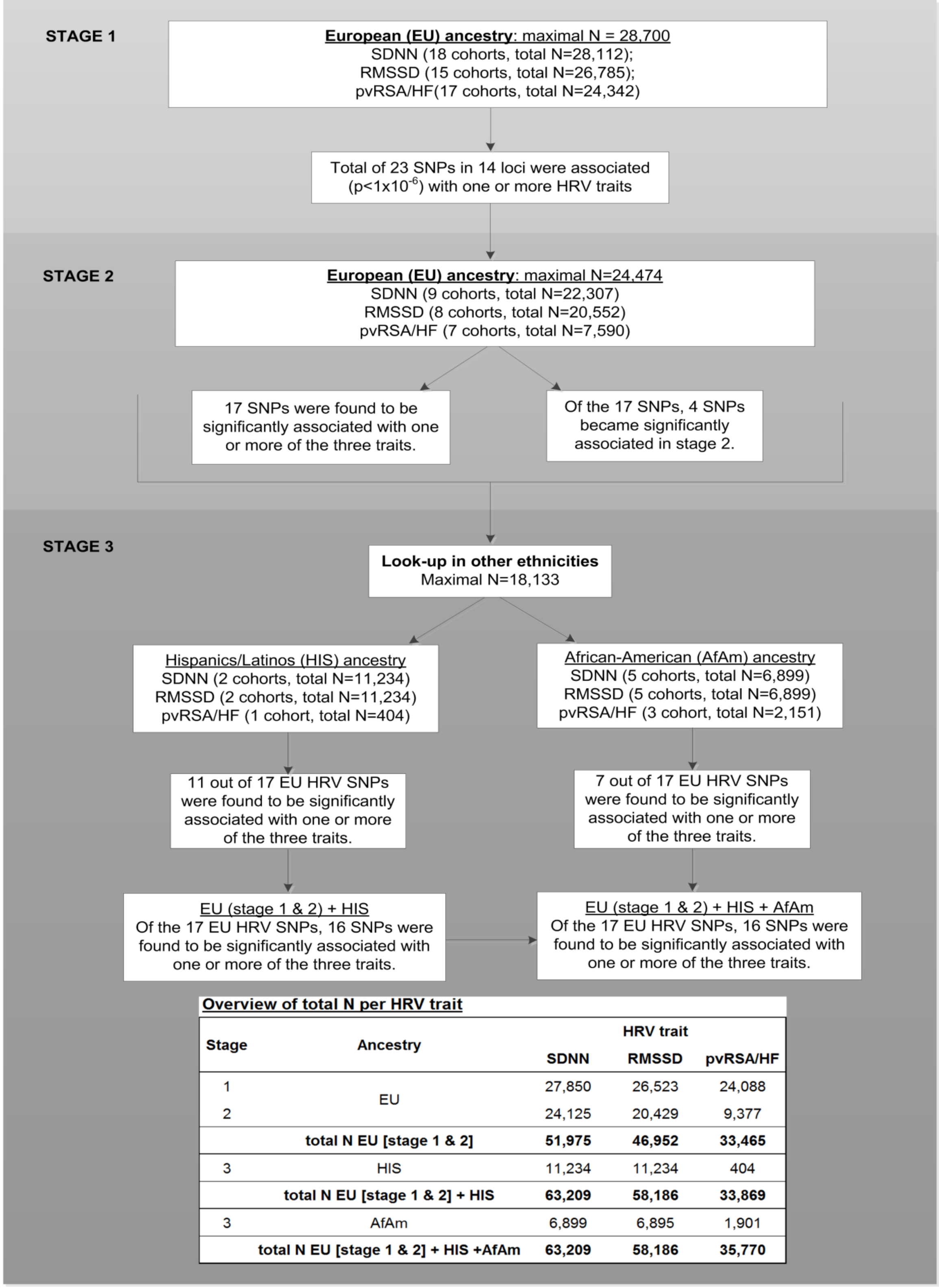

Overview of total N per HRV trait

Stage	Ancestry	HRV trait SDNN	RMSSD	pvRSA/HF
1	EU	27,850	26,523	24,088
2		24,125	20,429	9,377
	**total N EU [stage 1 & 2]**	**51,975**	**46,952**	**33,465**
3	HIS	11,234	11,234	404
	**total N EU [stage 1 & 2] + HIS**	**63,209**	**58,186**	**33,869**
3	AfAm	6,899	6,895	1,901
	**total N EU [stage 1 & 2] + HIS +AfAm**	**63,209**	**58,186**	**35,770**

Supplementary Figure 1: V_gHRV GWA meta-analysis study design.

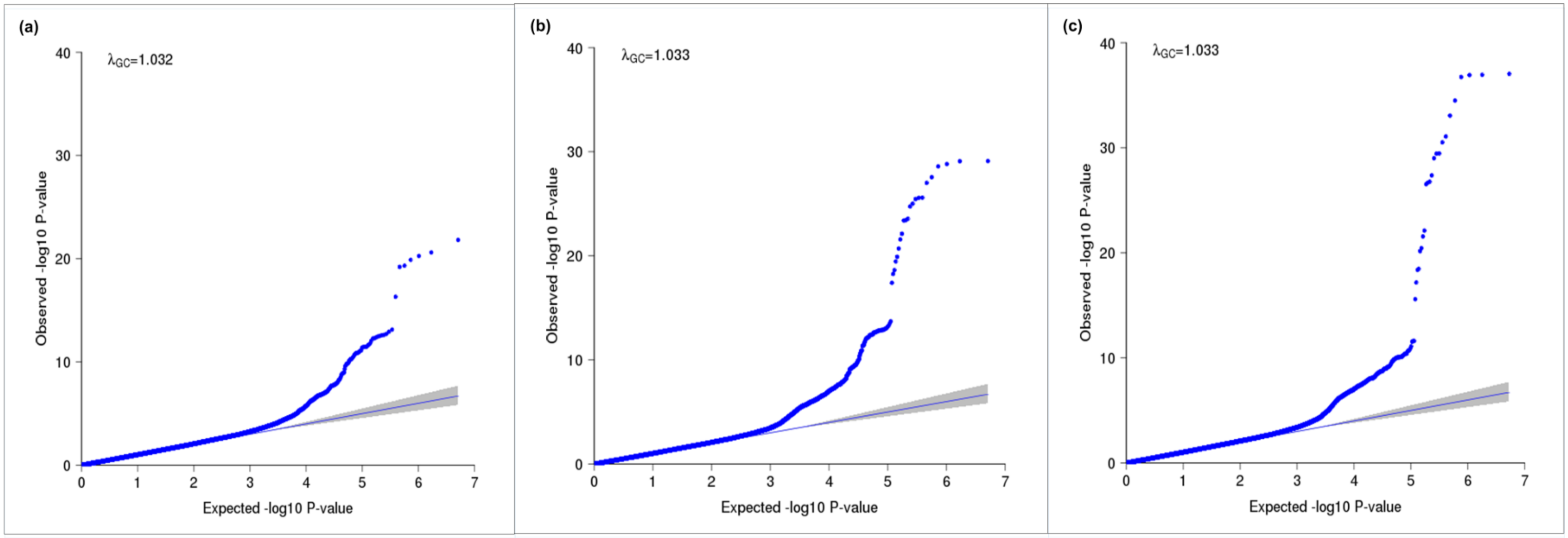

Supplementary Figure 2: Quantile-quantile-plots of the three meta-analyses of GWAS results of (a) SDNN, (b) RMSSD, and (c) pvRSA/HF for the stage 1 meta-analysis on individuals of European ancestry.

NOTE: Expected –$\log_{10}$(*p*-values) assuming a normal distribution are shown on the x-axis. The y-axis depicts the observed –$\log_{10}$(*p*-values). Only SNPs with a minor allele frequency >1% and that were present in at least 1/3 of the sample are shown.

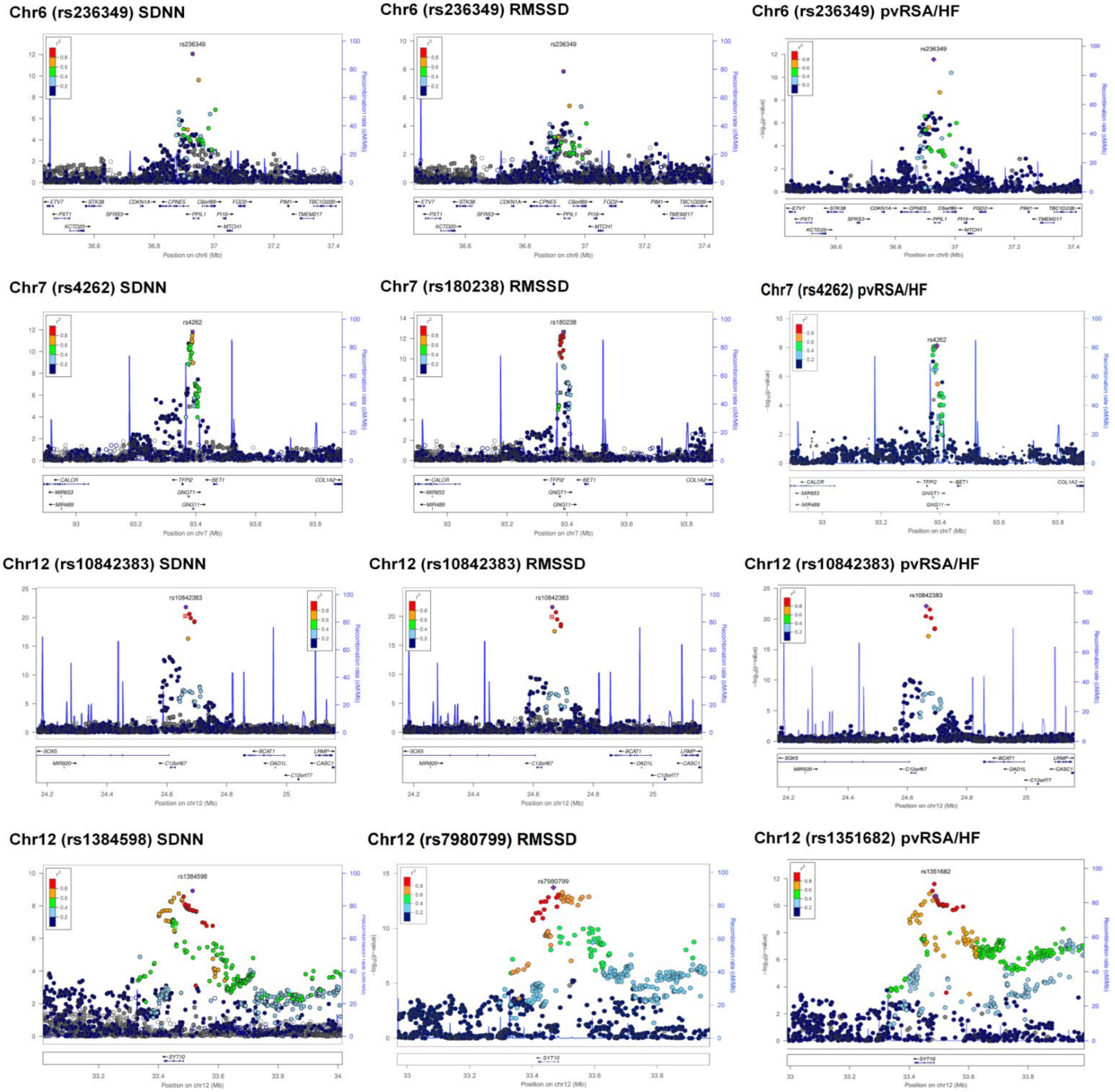

Supplementary Figure 3: Regional association plots of the 17 HRV SNPs from the stage 1 meta-analysis on individuals of European ancestry.

NOTE: Lead SNP and HRV trait are indicated in the top line. In the plots rs-numbers of the lead SNPs are given. Color coding reflects the LD (r^2) of the SNP with this lead SNP according to the legend. The left y-axis represent -$\log_{10}$(*p*-value) of association; the right y-axis indicates recombination rate. The x-axis depicts the position on the chromosome (Mb) and lists known genes near the locus. Symbols annotation: framestop (triangle), splice (triangle), non-synonymous (inverted triangle), synonymous (square), UTR (square), TFBScon [in a conserved region predicted to be a transcription factor binding site] (eight pointed star), MCS44 [in a region highly conserved within placental mammals] (square with two diagonal lines) and none-of-the-above (filled circle).

Chr14 (rs36423) SDNN

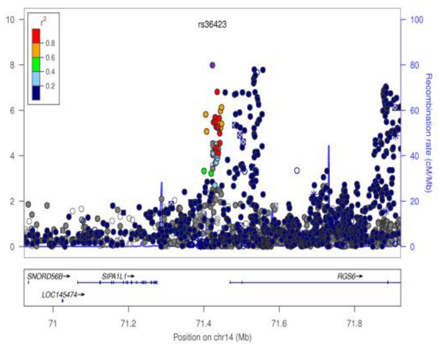

Chr14 (rs36423) RMSSD

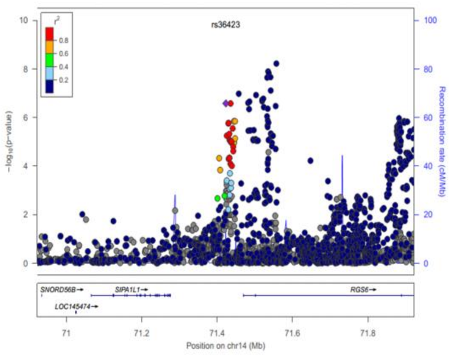

Chr14 (rs4899412) SDNN

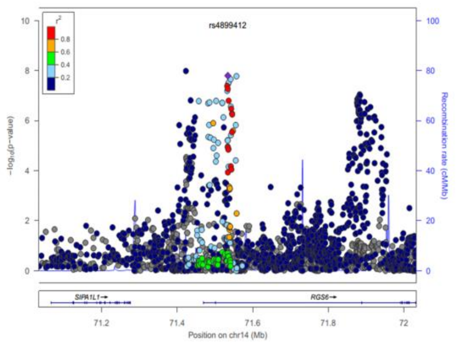

Chr14 (rs2052015) RMSSD

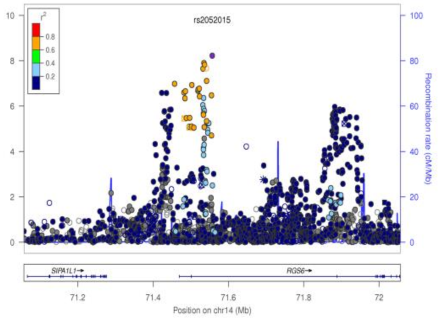

Chr14 (rs2529471) SDNN

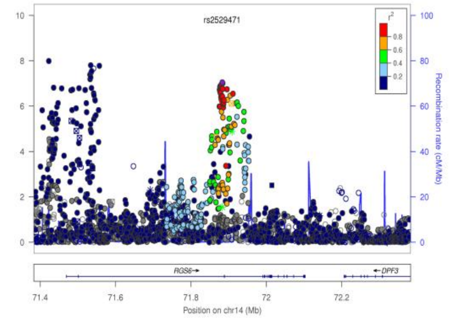

Chr15 (rs2680344) SDNN

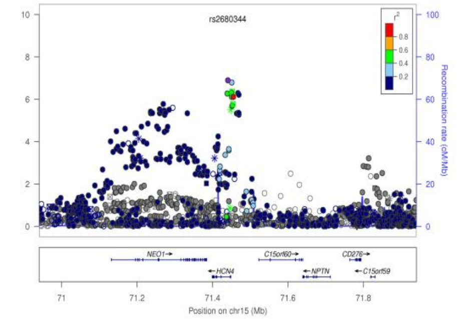

Chr15 (rs1812835) RMSSD

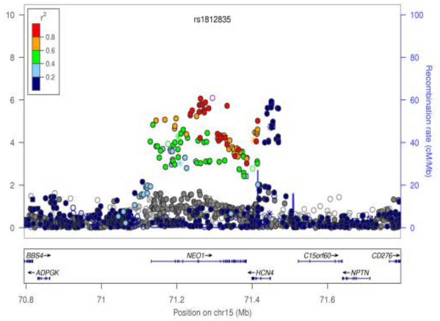

Chr19 (rs12980262) SDNN

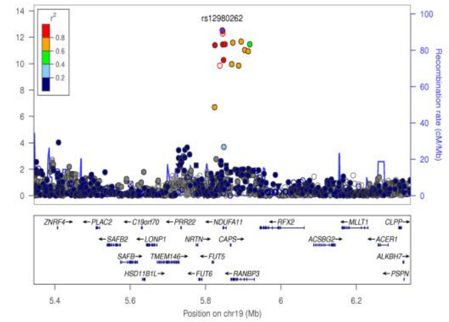

Chr19 (rs12974991) RMSSD

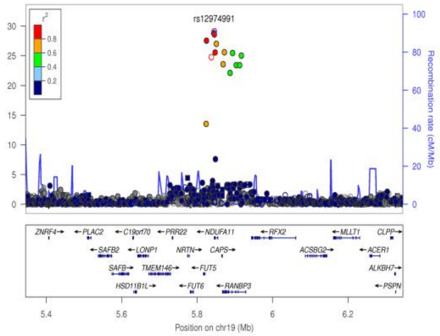

Chr19 (rs12974440) pvRSA/HF

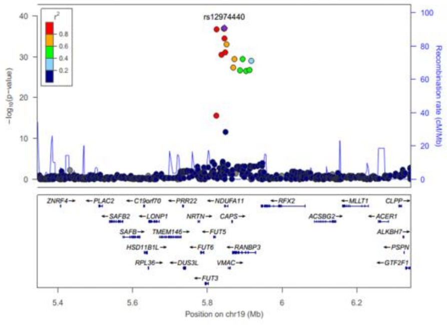

Chr20 (rs6123471) RMSSD

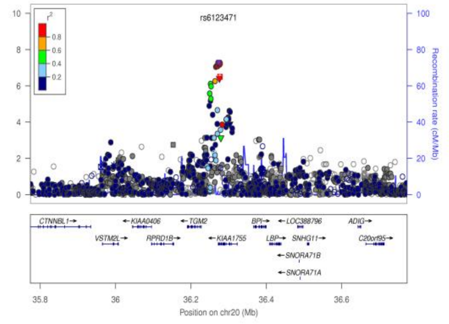

Supplementary Figure 3 (continued)

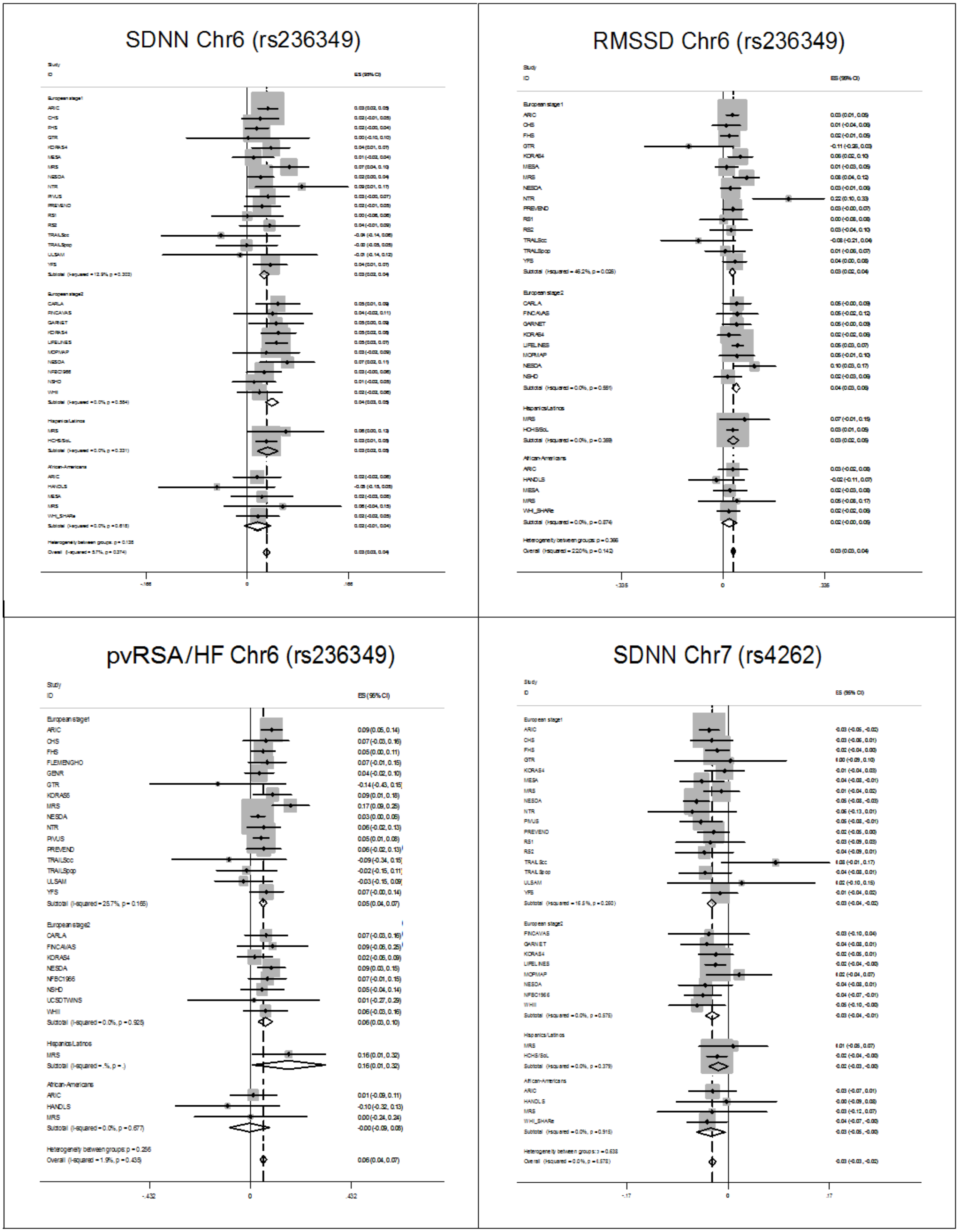

Supplementary Figure 4: Forest plots of the 17 HRV SNPs showing the effect sizes from the individual cohorts and from the stage 1, 2, and 3 meta-analyses on individuals of European ancestry, Hispanic/Latino ancestry, and African American ancestry.
NOTE: Meta-analysis derived effect sizes and 95% CI are given per stage and ancestry. The lower lines represent the heterogeneity test and the overall effect size based on all individuals from all ethnicities.

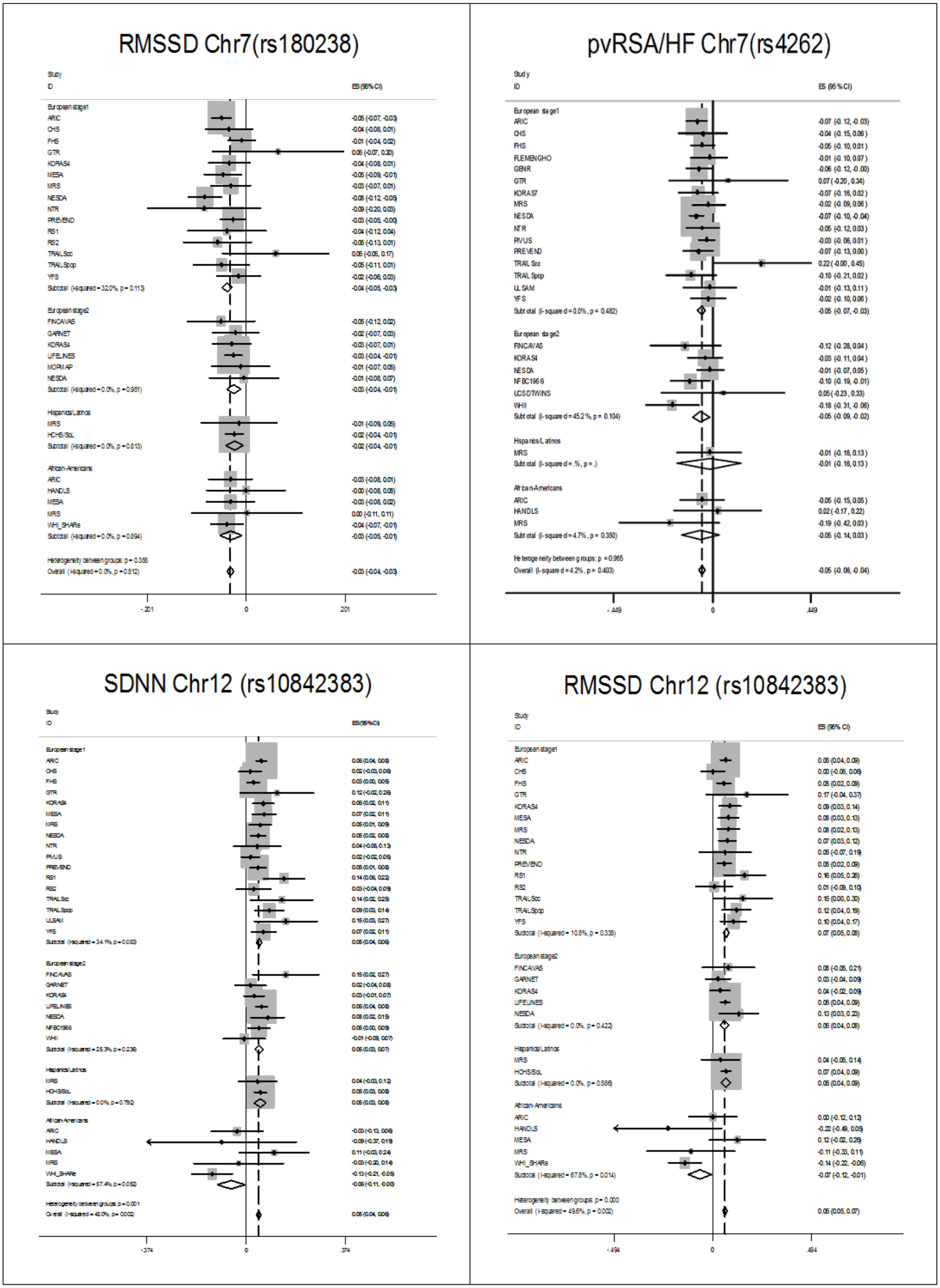

Supplementary Figure 4 (continued)

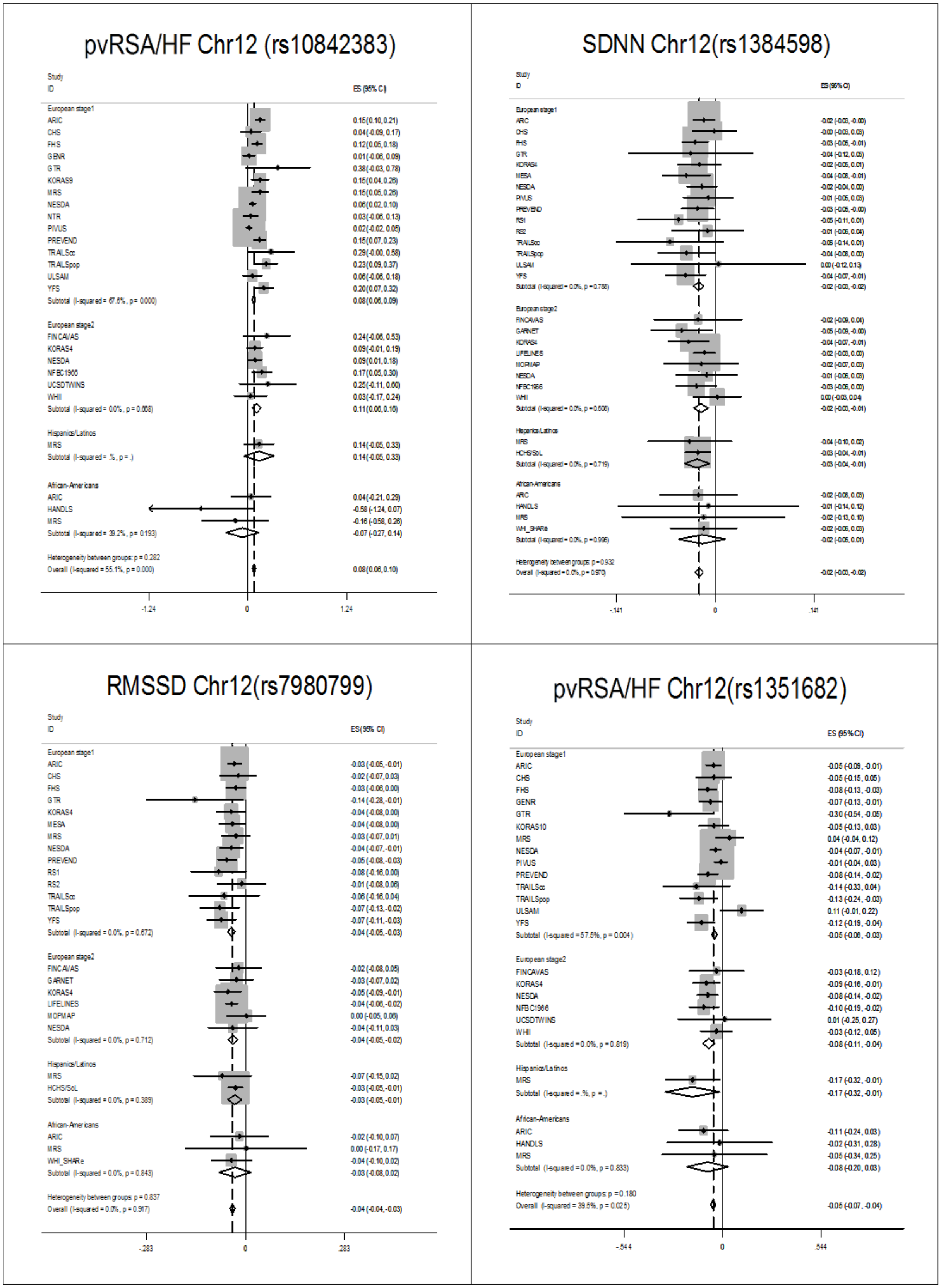

Supplementary Figure 4 (continued)

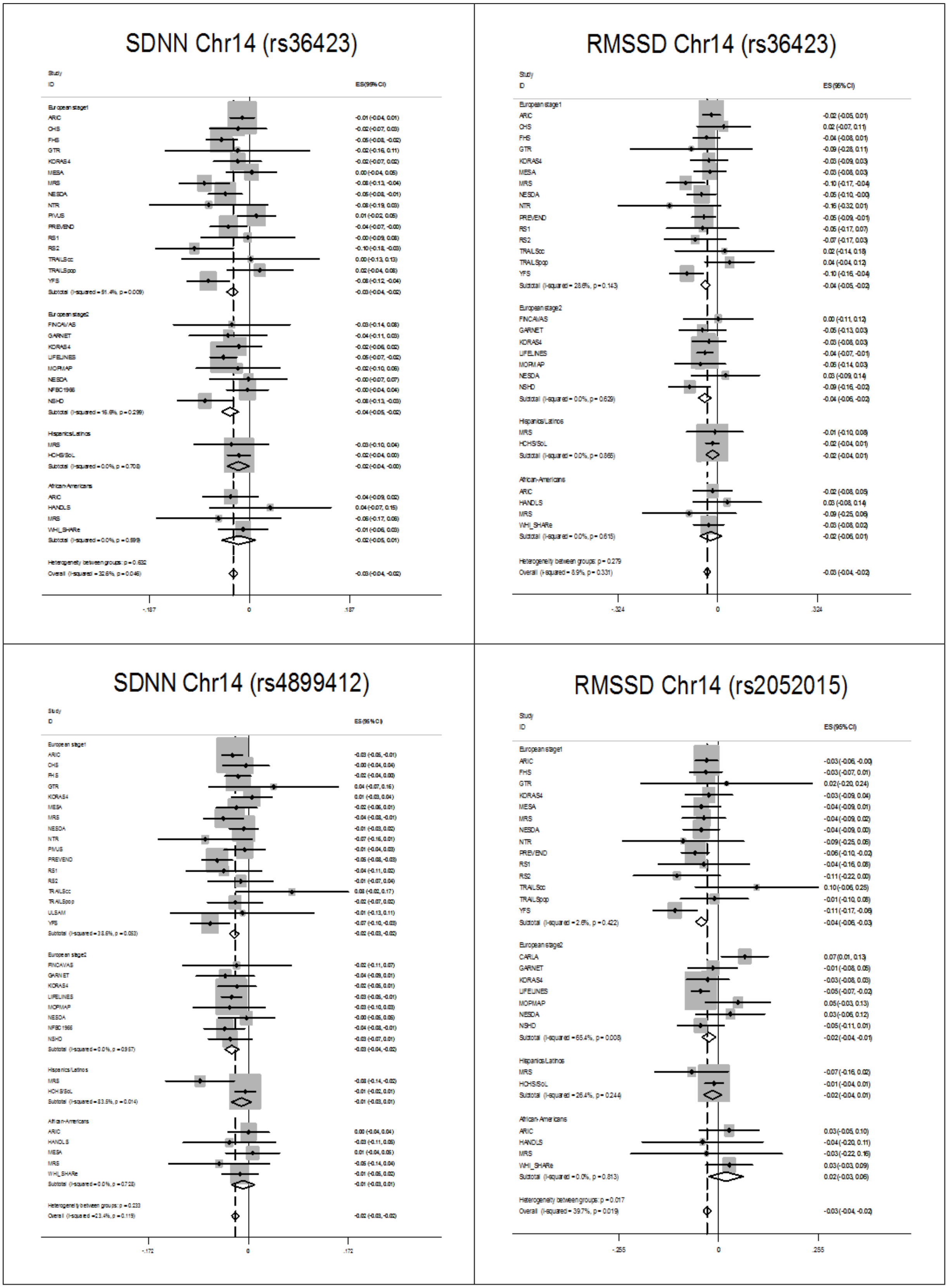

Supplementary Figure 4 (continued)

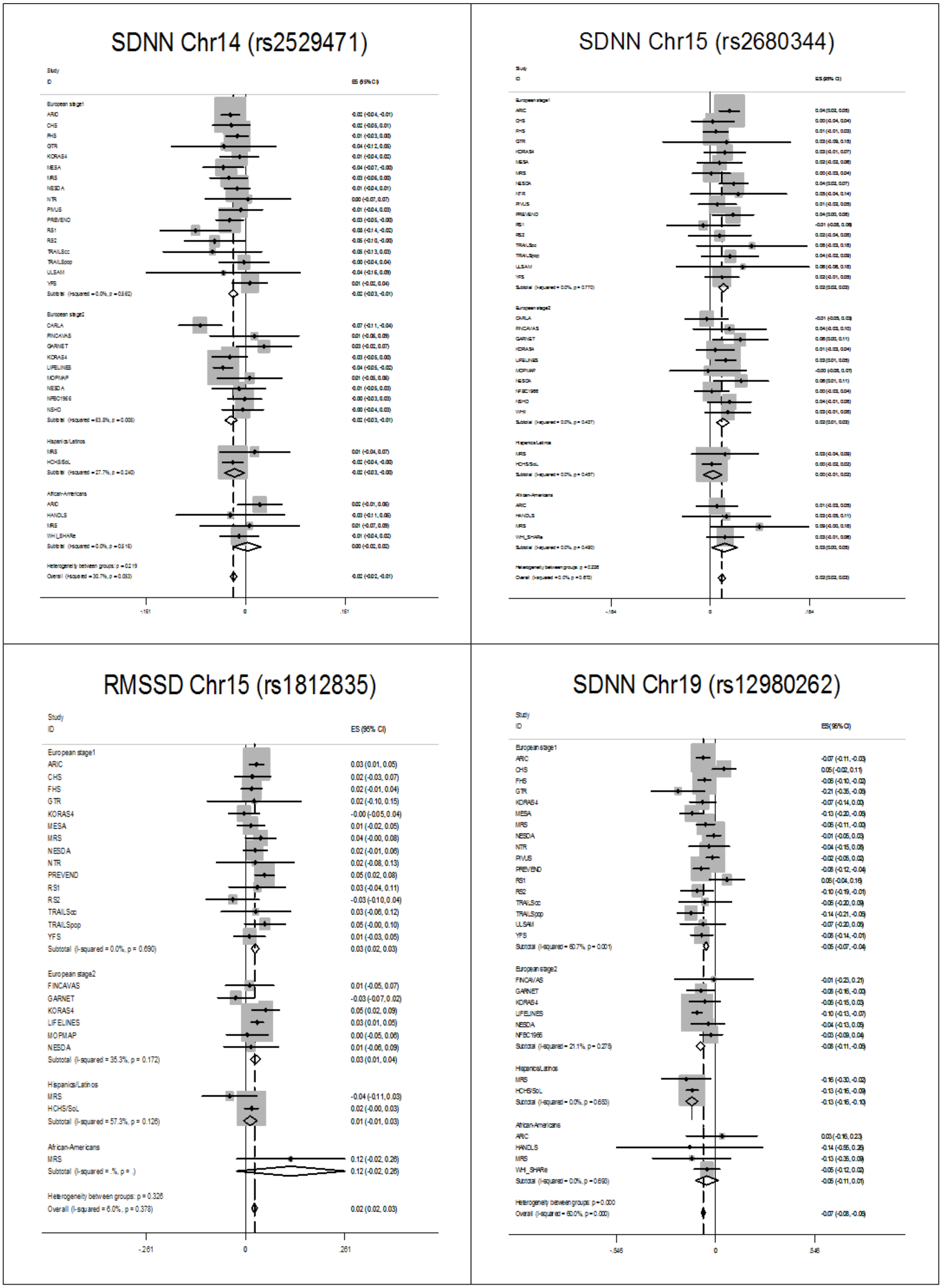

Supplementary Figure 4 (continued)

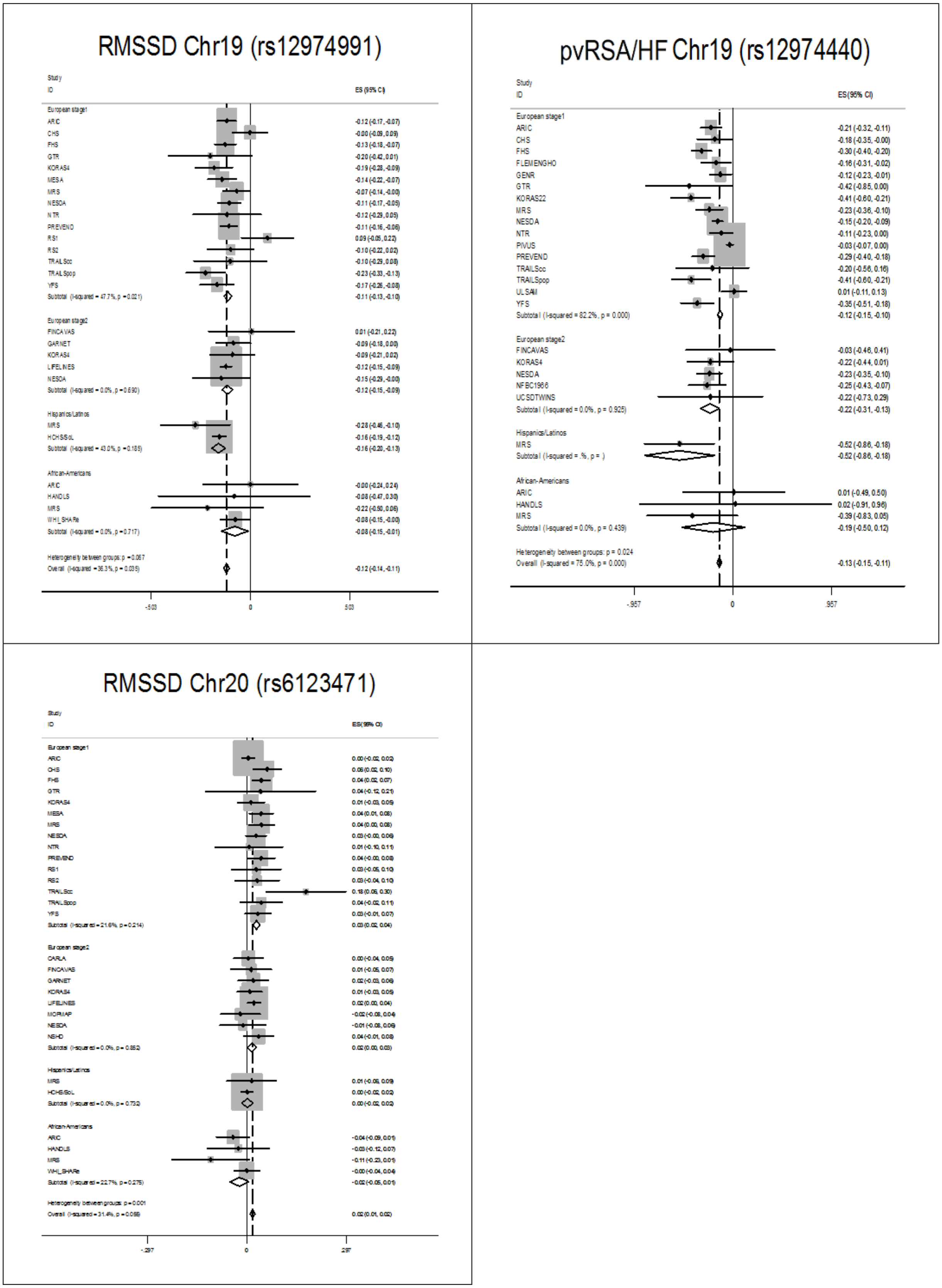

Supplementary Figure 4 (continued)

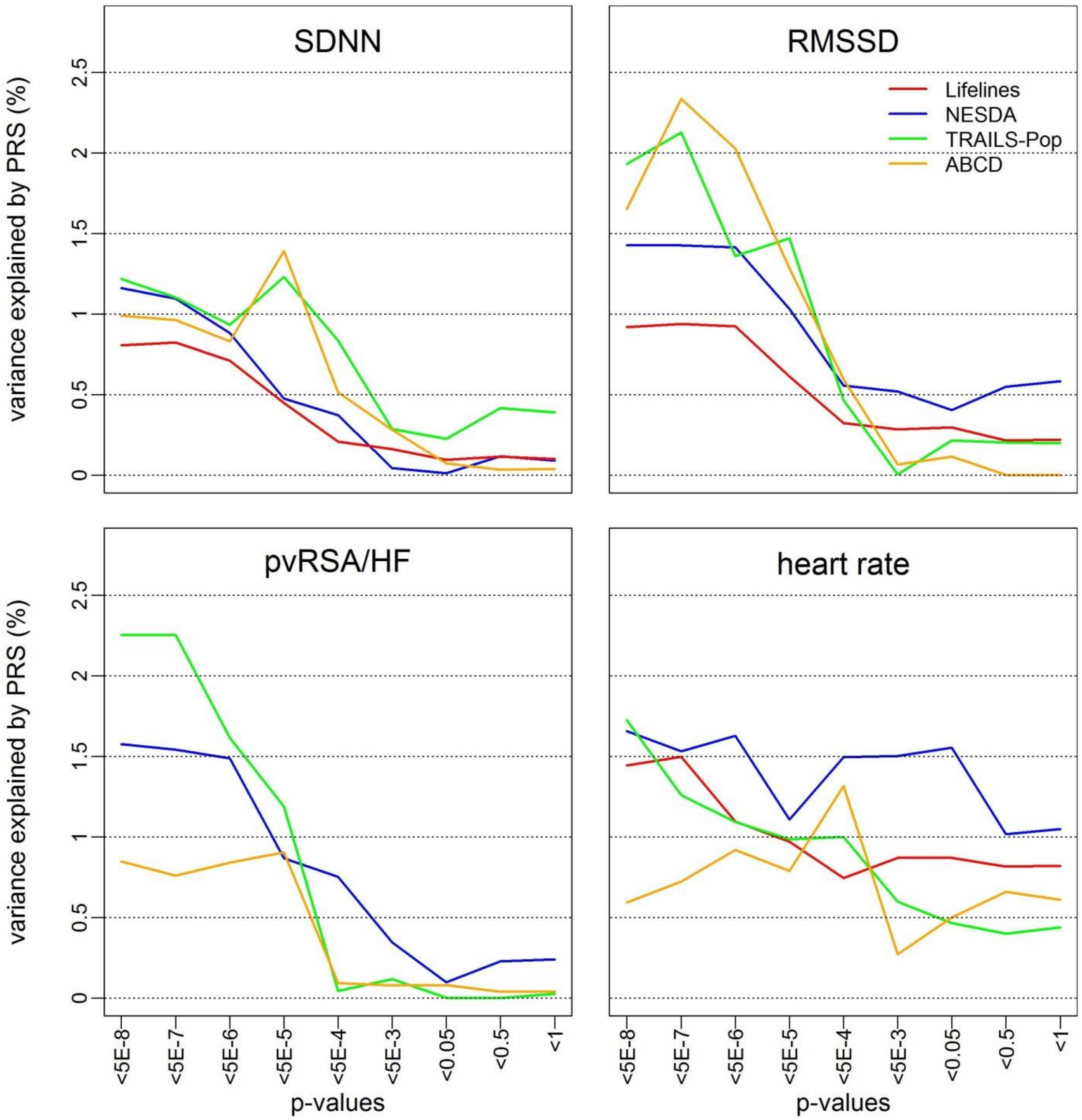

Supplementary Figure 5: Explained variance in HRV traits and heart rate by polygenic risk scores (PRS) for HRV in the Lifelines (n=12,101), NESDA (n=2,118), TRAILS-Pop (n=1,191), and ABCD (n=1,094) cohorts.

NOTE: Significance thresholds to include HRV SNPs in the polygenic risk scores are shown on the x-axis and were $p < 5.0\text{x}10^{-8}$; $p < 5.0\text{x}10^{-7}$; $p < 5.0\text{x}10^{-6}$; $p < 5.0\text{x}10^{-5}$; $p < 5.0\text{x}10^{-4}$; $p < 5.0\text{x}10^{-3}$; $p < 0.05$; $p < 0.5$; and $p < 1$. The y-axis depicts the percentage of variance explained by the polygenic risk score.

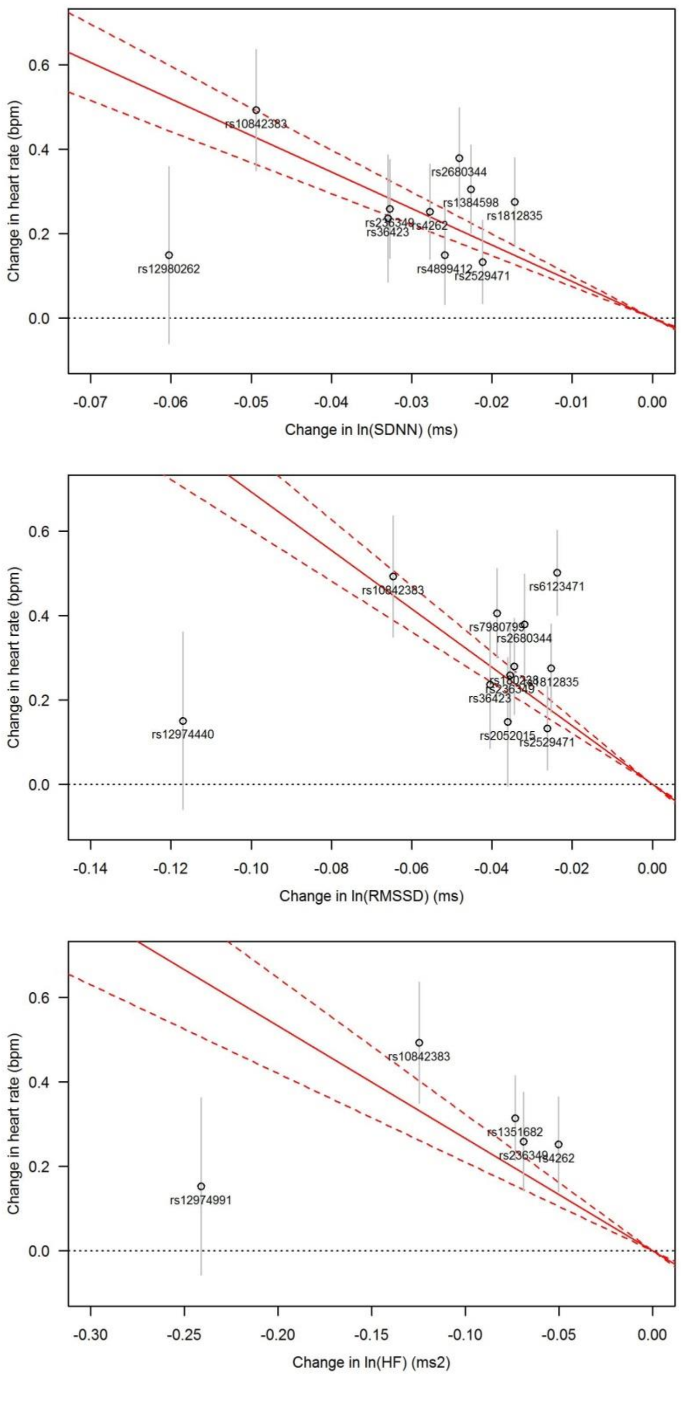

Supplementary Figure 6: Diagnostic plot for the association between genetic risk alleles for the HRV traits and heart rate.

NOTE: Each SNP is plotted by its decreasing effect on HRV per risk effect allele (x-axis) versus the estimated effect of that allele on the risk for high heart rate (y-axis). A solid red line shows the effect size estimate, called α, for the risk score on HRV. The 95% CI of α is represented by red dashed lines. The grey vertical lines indicate the 95% confidence interval (CI) of the effect on heart rate for each individual SNP. The estimated effects on heart rate are in beats per minute (bpm). Graphs from top to bottom are for SDNN, RMSSD, and pvRSA/HF, respectively.

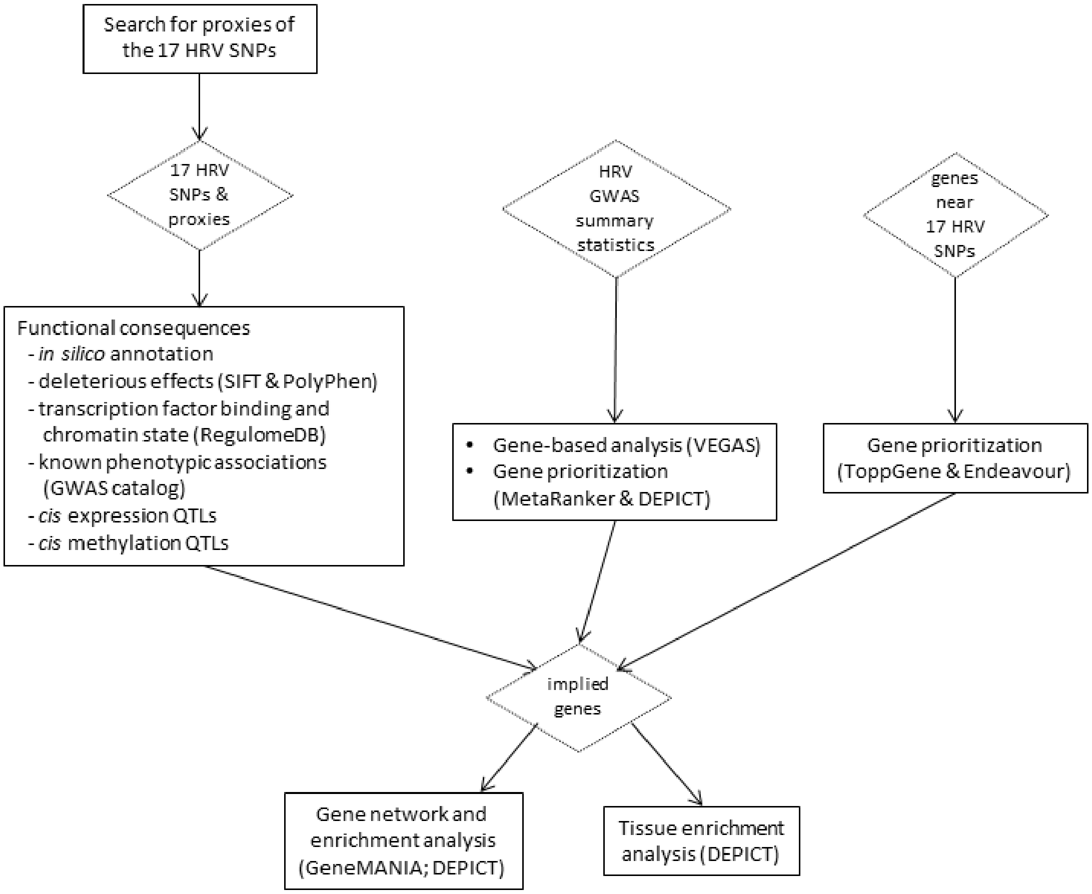

Supplementary Figure 7: Flowchart depicting the post-GWAS steps to annotate the HRV SNPs using public online resources**:** SIFT [1], PolyPhen [2], RegulomeDB [3] , VEGAS [4], MetaRanker [5], DEPICT, [6], ToppGene [7], Endeavour [8], and GeneMANIA [9].

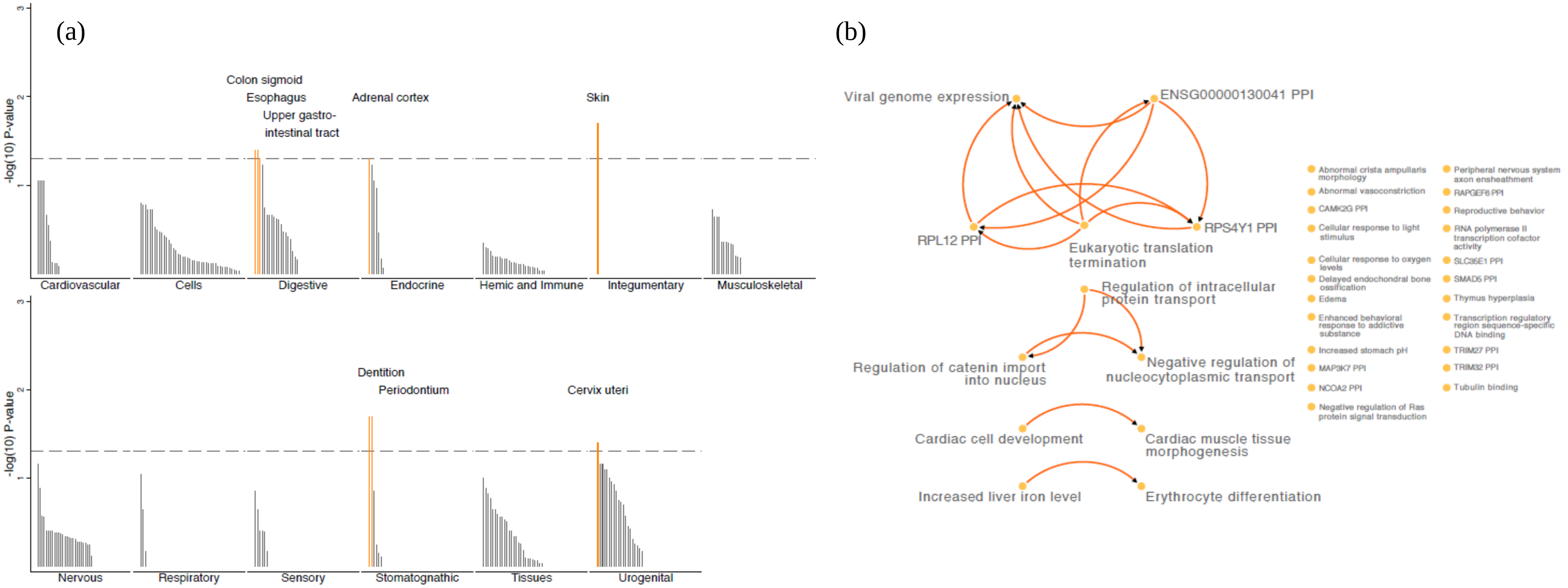

Supplementary Figure 8: Tissue enrichment and reconstituted gene set enrichment analysis using DEPICT for (a-b) SDNN; (c-d) RMSSD; and (e-f) pvRSA/HF.
NOTE: In panels (a), (c), and (e) graphical representations of DEPICT's tissue enrichment analysis are provided, highlighting annotated tissues that are enriched for expression of genes located within ±40kb of SNPs associated with $p < 10^{-5}$ to SDNN, RMSSD, and pvRSA/HF, respectively (enrichment in orange $p < 0.05$). In panels (b), (d), and (f) graphical representations of DEPICT's reconstituted gene set enrichment analysis are shown ($p < 0.05$ after Bonferroni correction) for examining SDNN, RMSSD, and pvRSA/HF, respectively. DEPICT calculates every gene's likelihood to be a member of KEGG, GEO, or REACTOME-based gene sets amongst others (N=14,461) resulting in reconstituted gene sets. Shown are the reconstituted gene sets enriched for genes in SDNN-, RMSSD-, and pvRSA/HF-associated loci (nodes), respectively, and the significant interactions between these gene sets (edges).

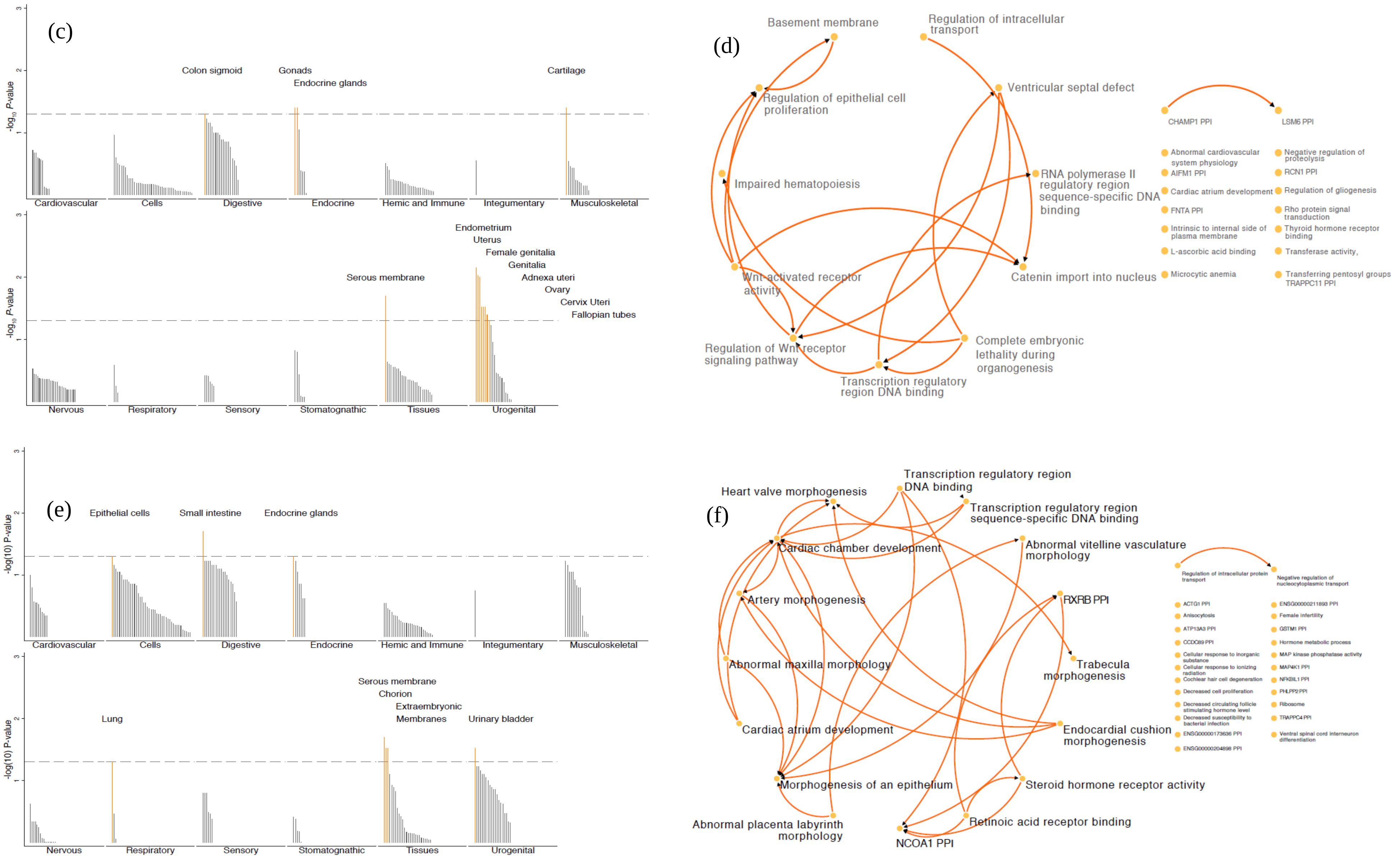

Supplementary Figure 8 (continued)

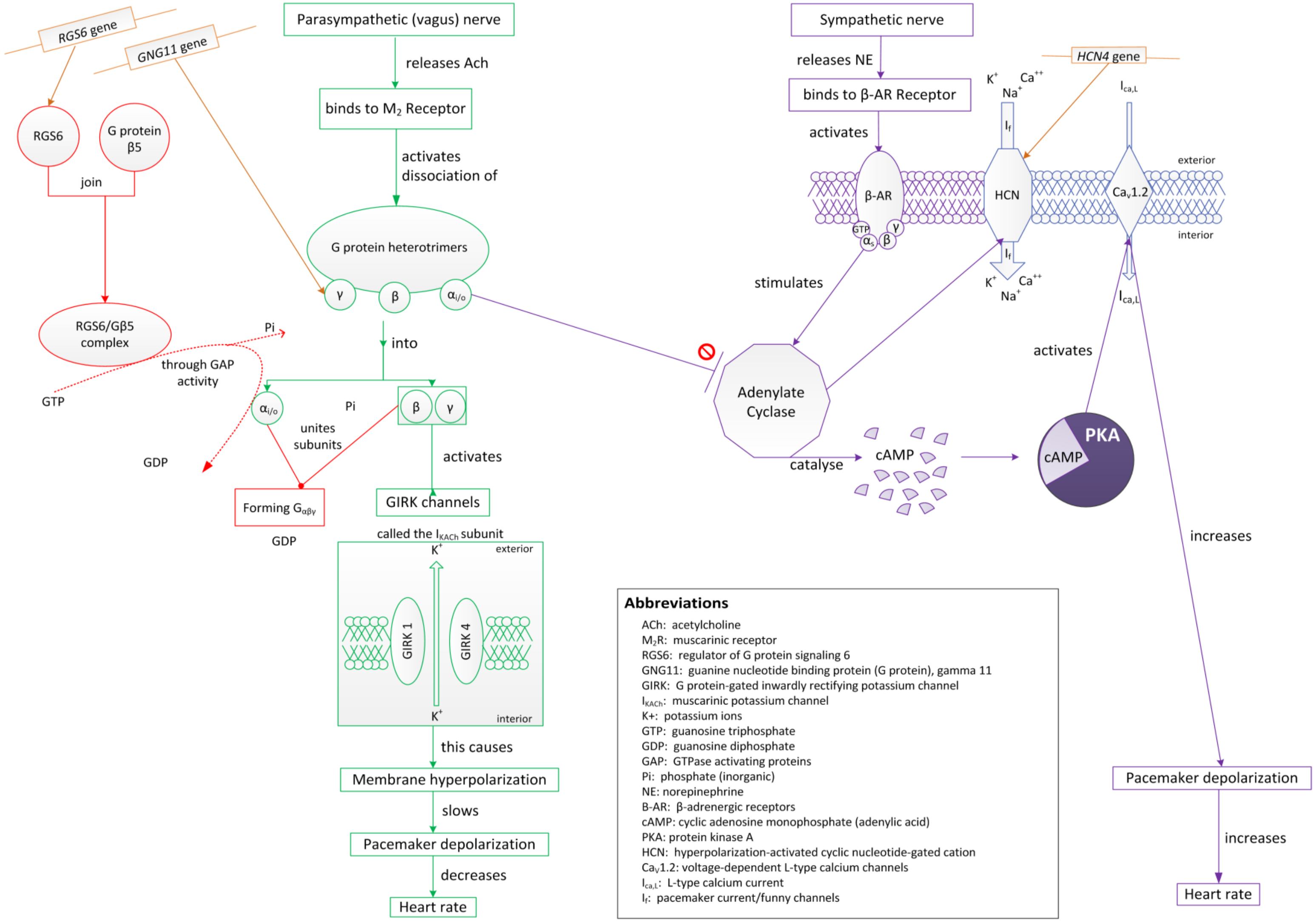

Supplementary Figure 9: A schematic representation of known cardiac vagal effects on the sinoatrial node and the potential roles of HRV SNPs in *GNG11*, *RGS6*, and *HCN4*. See for more details on next page.

Various pathways convey the effects of cardiac autonomic activity on heart rate regulation[10, 11]. To these known pathways we added the hypothesized effects of some of the HRV SNPs.
Green colored pathway: The vagal nerve releases acetylcholine (ACh), which binds to the muscarinic (M2R) receptor. This dissociates the inactive G protein heterotrimer (Gαβγ) composed of three subunits (α, β, and γ) into two components, namely the α subunit and a Gβγ component. The Gβγ component then interacts and activates the G protein-gated inwardly rectifying potassium (GIRK) channel, which is a potassium channel (I_{KACh}) composed of GIRK1 and GIRK4 subunits. This causes potassium ions (K^+) to permeate outwardly, which results in a membrane hyperpolarization slowing pacemaker depolarization and subsequently decreasing heart rate. We hypothesize that HRV SNPs in *GNG11* may lower the availability of the γ11 subunit and could reduce Gβγ component induced GIRK activation, which is expected to blunt the effects of phasic changes in cardiac vagal activity, thereby decreasing HRV.
Red colored pathway: RGS6 binds with G-protein β5 to create the RGS6/Gβ5 dimer complex, which activates GTPase activating proteins (GAPs), regulatory proteins that hydrolyze guanosine triphosphate (GTP) breaking a phosphate bond (Pi) to make guanosine diphosphate (GDP) on the α subunit. This causes the Gαi/o subunit and a Gβγ component to rejoin into the inactive Gαβγ heterotrimer. This ends GIRK channel activation and leads to an increase in heart rate.
We hypothesize the HRV SNP in *RGS6* to increase the availability of RGS6, which gives rise to a decrease in GIRK channel signaling, blunting the effects of phasic changes in cardiac vagal activity, thereby decreasing HRV.
Purple colored pathway: Catecholamine-mediated activation of β-adrenergic receptors also act as a guanine nucleotide exchange factor to dissociate the Gαβγ, with the GTP-Gαs then causing adenylate-cyclase to catalyze cAMP production. cAMP-dependent protein kinases then activate the L-type Ca,1,2 channels that increase depolarization of the pacemaker membrane, which leads to an increase in tonic heart rate.
Blue colored pathway: Through this same adenylate/cAMP signaling pathway, vagal activation of the M2R also impacts on the funny channels (I_f) of which the hyperpolarization-activated cyclic nucleotide-gated channel isoform 4 (HCN4) is the predominant molecular constituent. The I_f is permeable to K^+, Na^+ and Ca^{++} yielding a net inward current that plays a key role in the generation of the pacemaker potential[12, 13]. The unique property of reverse voltage dependence of the funny channel causes a spontaneous gradual depolarization of the pacemaker membrane until the action potential threshold, at which potential the systolic phase of the next heartbeat commences, whereas the sympathetic Gαs subunit speeds up I_f diastolic depolarization, the vagal Gαi/o subunit counters this by slowing the I_f diastolic depolarization.
We hypothesize the HRV SNP in *HCN4* to increase permeability of the HCN channel to speed up depolarization of the pacemaker membrane, thereby increasing tonic heart rate and reducing HRV.

(a) See VgHRV_Supplementary_Figure10a.png

(b) See VgHRV_Supplementary_Figure10b.png

Supplementary Figure 10: Functional enrichment analysis based on 24 query genes resulting from the GWAS meta-analyses and post-GWAS analyses on the three HRV traits.
NOTE: The following genes were used as input for GeneMANIA: *NDUFA11*, *FUT5*, *CAPS*, *PPIL1*, *C6orf89*, *SYT10*, *GNG11*, *GNGT1*, *RGS6*, *HCN4*, *NEO1*, *KIAA1755*, *CCDC141*, *TFPI2*, *ALG10*, *ALG10B*, *CPNE8*, *NRTN*, *FUT6*, *FUT3*, *VMAC*, *RFX2*, *RANBP3*, and *CPNE5*. The resulting significantly enriched gene ontology terms (false discovery rate<0.10) are visualized as highlighted boxes within their corresponding gene ontology tree depicted by RamiGO R package, as (a) yellow category: terms related to cell membrane signal transduction, and (b) green category: terms related to cellular anabolic, catabolic, and respiratory processes. The relations between the boxes have standard color scheme: green, red, black, blue, and light blue represent ‘positively regulates’, ‘negatively regulates’, ‘regulates’, ‘is a’, and ‘part of’, respectively (http://www.geneontology.org/GO.ontology-ext.relations.shtml).

SUPPLEMENTARY TABLES

Supplementary Table 1: Description of stage 1 discovery, stage 2 replication, stage 3 other ethnicity lookup, and post-GWAS cohorts and lookup trait/disease consortia used in the V_gHRV analyses.

Study acronym	Study full name	Study design	Country	Ethnicity	Study reference
**STAGE 1 DISCOVERY COHORTS**					
ARIC	Atherosclerosis Risk in Communities study	prospective population-based cohort study	USA	EUR	PMID: 2646917
CHS	Cardiovascular Health Study	prospective population-based cohort study	USA	EUR	PMID: 1669507
FHS	Framingham Heart Study	prospective population-based cohort study	USA	EUR	PMID: 14819398
FINGESTURE	FINnish GEnetic STUdy of aRrhythmic Events	prospective case-control study (cases: post-AMI patients)	Finland	EUR	ClinicalTrials.gov Identifier: NCT02075866
FLEMENGHO-EPOGH	FLEMish study on Environment, Genes and Health Outcomes – European Project on Genes in Hypertension	prospective population-based cohort study	Belgium, Romania, Poland, Italy, Russian Federation and Czech Republic	EUR	PMID: 12640246 & 22184326
GenR	Generation R Study	prospective population-based cohort study of infants	Netherlands	EUR	PMID: 16826450
GTR	Groningen Twin Registry	prospective twin study	Netherlands	EUR	PMID: 23186546
KORA S4	KOoperative gesundheitsforschung in der Region Augsburg – Survey 4	prospective population-based cohort study	Germany	EUR	PMID: 16032513 & 16032514
MESA	Multi-Ethnic Study of Atherosclerosis	prospective population-based cohort study	USA	EUR	PMID: 12397006
MRS	Marine Resiliency Study	prospective population-based cohort study	USA	EUR	DOI: http://dx.doi.org/10.5888/pcd9.110134; PMID: 25456346

NESDA	Netherlands Study of Depression and Anxiety	prospective case-control study (cases: MDD patients)	Netherlands	EUR	PMID: 18763692
NTR	Netherlands Twin Register	prospective twin-family study	Netherlands	EUR	PMID: 12537867
PIVUS	Prospective Investigation of the Vasculature in Uppsala Seniors	prospective population-based cohort study	Sweden	EUR	PMID: 16141402
PREVEND	Prevention of Renal and Vascular ENd-stage Disease	prospective population-based cohort study enriched for individuals with microalbuminuria	Netherlands	EUR	PMID: 11004219
RS(1+2)	Rotterdam Study	prospective population-based cohort study	Netherlands	EUR	PMID: 24258680
TRAILS-CC	TRacking Adolescents Individual Lives Survey – CliniCal cohort	prospective high-risk cohort study of adolescents	Netherlands	EUR	PMIDs: 25431468, 25066533
TRAILS-Pop	TRacking Adolescents Individual Lives Survey – POPulation cohort	prospective population-based cohort study of adolescents	Netherlands	EUR	see TRAILS-CC
ULSAM	Uppsala Longitudinal Study of Adult Men	prospective population-based cohort study	Sweden	EUR	PMID: 1216390; http://www2.pubcare.uu.se/ULSAM/
YFS	Cardiovascular Risk in Young Finns Study	prospective population-based cohort study	Finland	EUR	PMID: 18263651; http://youngfinnsstudy.utu.fi/
**STAGE 2 REPLICATION COHORTS**					
CARLA	CARdiovascular disease, Living and Ageing in Halle	prospective population-based cohort study	Germany	EUR	PMID: 19199053
FINCAVAS	FINnish CArdioVAscular Study	prospective population-based cohort study	Finland	EUR	PMID: 16515696
KORA S4	KOoperative gesundheitsforschung	prospective population-based cohort study	Germany	EUR	PMID: 16032513

	in der Region Augsburg – Survey 4				
Lifelines	Lifelines Cohort Study	prospective population-based cohort study	Netherlands	EUR	PMID: 18075776
MRC NSHD	Medical Research Council National Survey of Health and Development	prospective population-based birth cohort study	UK	EUR	PMID: 21345808; http://www.nshd.mrc.ac.uk
NESDA	Netherlands Study of Depression and Anxiety	prospective case-control study (cases: MDD patients)	Netherlands	EUR	PMID: 18763692
NFBC 1966	Northern Finland Birth Cohort 1966	prospective population-based birth cohort study	Finland	EUR	PMID 19060910
UCSD TWINS	University of California San Diego TWINS	prospective twin study	USA	EUR	PMID: 22676942
WHI CT – GARNET	Women's Health Initiative Clinical Trials (Genomics And Randomized Trials Network) controls	prospective cohort study	USA	EUR	PMID: 9492970 and http://www.genome.gov/27541119
WHI CT – MOPMAP	Women's Health Initiative Clinical Trials (Modification Of PM-Mediated Arrhythmogenesis in Populations) controls	prospective cohort study	USA	EUR	PMID: 9492970 and http://projectreporter.nih.gov/project_info_description.cfm?aid=7984809& icde=19283008
WHII	Whitehall-II study	prospective population-based cohort study	UK	EUR	PMID: 15576467
**STAGE 3 LOOKUP COHORTS OF OTHER ETHNICITY**					
HCHS/SOL	Hispanic Community Health Study/Study of Latinos	multicenter community-based cohort study	USA	HIS	PMID: 20609343, 20609344
MRS	Marine Resiliency Study	prospective population-based cohort study	USA	HIS	DOI: http://dx.doi.org/10.5888/pcd9.110134; PMID: 25456346
ARIC	Atherosclerosis Risk in Communities study	prospective population-based cohort study	USA	AfAm	PMID: 2646917

HANDLS	Healthy Aging in Neighborhoods of Diversity across Life Span	prospective community-based study	USA	AfAm	PMID: 20828101
MESA	Multi-Ethnic Study of Atherosclerosis	prospective population-based cohort study	USA	AfAm	PMID: 12397006
MRS	Marine Resiliency Study	prospective population-based cohort study	USA	AfAm	DOI: http://dx.doi.org/10.5888/pcd9.110134; PMID: 25456346
WHI CT – SHARe	Women's Health Initiative Clinical Trials (Single nucleotide polymorphism Health Association Resource)	prospective cohort study	USA	AfAm	PMID: 9492970 and https://www.nhlbi.nih.gov/resources/geneticsgenomics/programs/share.htm
**POST-GWAS COHORTS & CONSORTIA providing lookups**					
ABCD	Amsterdam Born Children and their Development	population-based	Netherlands	EUR	PMID: 20813863
OFS	Oman Family Study	five large inbred pedigrees	Oman	Arabic	PMID: 15767758
CHARGE-HF	CHARGE-Heart Failure Working Group	GWAS Consortium	multiple	EUR	PMID 20445134
CKDGen	CKDGen Consortium	GWAS Consortium	multiple	EUR	PMID: 21355061, 22479191
ICBP	International Consortium for Blood Pressure	GWAS Consortium	multiple	EUR	PMID: 21909115
AFGen Consortium	AFGen Consortium	GWAS Consortium	multiple	EUR	PMID: 22544366
CHARGE-SCD	CHARGE-SCD Consortium	GWAS Consortium	multiple	EUR	PMID:21738491

EUR: European; HIS: Hispanic/Latino; AfAm: African American; PMID: PubMed ID; NCT: ClinicalTrails.gov identifier; DOI: digital object identifier.

Supplementary Table 2: Phenotyping information of stage 1 discovery, stage 2 replication, stage 3 other ethnicity lookup, and post-GWAS cohorts used in the V_gHRV analyses.

Study acronym	Analysis sample size (N)	HRV assessment method	HRV measurement	Female/male participation
**STAGE 1 DISCOVERY COHORTS**				
ARIC	8262	3-lead ECG; 2 minutes; supine	RMSSD; SDNN; HF	Men and women
CHS	759	24hr Holter monitor	RMSSD; SDNN; HF	Men and women
FHS	1944	2hr ambulatory ECG	RMSSD; SDNN; HF	Men and women
FINGESTURE	494	24hr Holter monitor	SDNN; HF	Men and women
FLEMENGHO-EPOGH	196	12-lead ECG & nasal thermistor for RSA: PSA to estimate HF ranges ; ECG recording for 15 min ; supine	pvRSA	Men and women
GenR	392	3-pole ECG & breathing pattern using a piëzo-electric transducer ; 100-180 seconds ; sitting	HF	Men and women
GTR	134	type II 3-lead ECGs & respiration with a flexible band around upper thorax; 5 minutes; sitting	RMSSD; SDNN; HF	Women
KORA S4	1617	2-lead ECG ; 5 minutes ; supine	RMSSD; SDNN; HF	Men and women
MESA	2401	12-lead ECG; average from 3 sequential 10-second ECGs; supine; resting	RMSSD; SDNN	Men and women
MRS	1383	finger photoplethysmograph ; 5 minutes ; sitting	RMSSD; SDNN; HF	Men
NESDA	1740	type II, 3-lead ECG & breathing recorded from thorax impedance ; ~90 minutes ; sitting	RMSSD; SDNN; pvRSA	Men and women
NTR	439	type II, 3-lead ECG & breathing recorded from respitrace ; 8 minutes ; sitting	RMSSD; SDNN; pvRSA	Men and women
PIVUS	766	6-precordial-lead ECG & breathing recorded using custom-made impedance device ; 5-minutes ; supine ; controlled breathing (12 breaths/min)	SDNN; pvRSA	Men and women
PREVEND	2793	beat-to-beat blood pressure pulse wave recording on middle finger (Portapres); 15 minutes; supine	RMSSD; SDNN; HF	Men and women
RS1	972	12-lead ECG ; 10 seconds; resting	RMSSD; SDNN	Men and women
RS2	985	12-lead ECG ; 10 seconds; resting	RMSSD; SDNN	Men and women
TRAILS-CC	307	type II 3-lead ECG; 4 minutes (T1); supine	RMSSD; SDNN; HF	Men and women
TRAILS-Pop	1222	type II 3-lead ECG; 4 minutes (T1), 5 minutes (T3); supine	RMSSD; SDNN; HF	Men and women
ULSAM	67	6-precordial-lead ECG & breathing recorded using custom-made impedance device from a 24hr recording during normal activity	SDNN; pvRSA	Men
YFS	1827	2-lead ECG ; 3 minutes ; supine	RMSSD; SDNN; HF	Men and women

**STAGE 2 REPLICATION COHORTS**				
CARLA	1367	12-lead ECG ; 5 minutes for HRV analysis ; supine	RMSSD; SDNN; HF	Men and women
FINCAVAS	542	12-lead ECG ; 1 minute ; supine	RMSSD; SDNN; HF	Men and women
KORA S4	1959	2-lead ECG ; 5 minutes ; supine	RMSSD; SDNN; HF	Men and women
Lifelines	12101	12-lead ECG; 10 seconds; supine	RMSSD; SDNN	Men and women
MRC NSHD	1127	3 lead ECG ; 6 minutes ; supine	RMSSD; SDNN; HF	Men and women
NESDA	606	type II, 3-lead ECG & breathing recorded from thorax impedance ; ~90 minutes ; sitting	RMSSD; SDNN; pvRSA	Men and women
NFBC 1966	1941	lead-II ECG recording ; 3 minutes ; sitting	SDNN; HF	Men and women
UCSD TWINS	230	3 lead ECG ; 5 minutes; sitting	HF	Men and women
WHI CT – GARNET	1648	12-lead ECG; 10 seconds; resting; supine	RMSSD; SDNN	Women
WHI CT – MOPMAP	1198	12-lead ECG; 10 seconds; resting; supine	RMSSD; SDNN	Women
WHII	1755	12-lead ECG; 5 minutes; resting; supine	SDNN; HF	Men and women
**STAGE 3 LOOKUP COHORTS OF OTHER ETHNICITY**				
HCHS/SOL	10830	12-lead ECG; one 10-second ECG; supine; resting	RMSSD; SDNN	Men and women
MRS	404	finger photoplethysmograph & (simultaneous)2-lead ECG recording ; 5 minutes ; sitting	RMSSD; SDNN; HF	Men
ARIC	1582	3-lead ECG; 2 minutes; supine	RMSSD; SDNN; HF	Men and women
HANDLS	188	Portapres ambulatory HR and BP monitor; 5 minutes; sitting	RMSSD; SDNN; HF	Men and women
MESA	1480	12-lead ECG; average from 3 sequential 10-second ECGs; supine; resting	RMSSD; SDNN	Men and women
MRS	131	finger photoplethysmograph ; 5 minutes ; sitting	RMSSD; SDNN; HF	Men
WHI CT – SHARe	3518	12-lead ECG; 10 seconds; supine	RMSSD; SDNN	Women
**POST-GWAS COHORTS**				
ABCD	1094	type II, 3-lead ECG & breathing recorded from thorax impedance ; ~4 minutes ; sitting	RMSSD; SDNN; pvRSA	Men and women
OFS	1326	type II, 6-lead ECG ; 10 minutes; supine	RMSSD; SDNN; HF	Men and women

ECG: electrocardiogram; RSA: respiratory sinus arrhythmia; PSA: proportion of specific agreement; HF: high frequency; HR: heart rate; BP: blood pressure.

Supplementary Table 3: Clinical characteristics of stage 1 discovery, stage 2 replication, and stage 3 other ethnicity, and post-GWAS cohorts used in the V_gHRV analyses.

Study acronym	Analysis sample size (N)	Women (%)		Age	SDNN	ln(SDNN)	RMSSD	ln(RMSSD)	pvRSA/HF	ln(pvRSA/HF)
				Mean (SD)						
**STAGE 1 DISCOVERY COHORTS**										
ARIC	8.262	54.9	overall	54.04 (5.65)	37.58 (19.14)	3.52 (0.46)	27.87 (21.73)	3.15 (0.58)	16.89 (44.33)	2.03 (1.27)
			women	53.74 (5.63)	36.27 (18.16)	3.49 (0.45)	27.96 (20.37)	3.16 (0.58)	17.96 (37.96)	2.16 (1.24)
			men	54.41 (5.66)	39.18 (20.16)	3.56 (0.46)	27.75 (23.28)	3.14 (0.58)	15.59 (51.00)	1.87 (1.29)
CHS	759	58.1	overall	71.2 (4.5)	96.7 (28.4)	4.5 (0.3)	21.5 (11.8)	2.9 (0.4)	115.8 (172.1)	4.3 (0.8)
			women	70.6 (4.1)	93.1 (25.4)	4.5 (0.3)	20.3 (9.1)	2.9 (0.4)	104.9 (116.6)	4.3 (0.8)
			men	72.0 (4.9)	101.6 (32.0)	4.6 (0.3)	23.2 (14.8)	3.0 (0.5)	130.8 (227.7)	4.3 (0.9)
FHS	1.944	53.86	overall	51.70 (12.85)	91.76 (27.97)	4.47 (0.31)	33.54 (16.61)	3.41 (0.44)	34.07 (38.51)	5.44 (0.88)
			women	52.23 (13.06)	93.60 (27.79)	4.45 (0.32)	33.94 (16.76)	3.40 (0.44)	33.91 (37.67)	5.44 (0.89)
			men	51.09 (12.60)	89.61 (28.18)	4.49 (0.30)	33.08 (16.43)	3.42 (0.44)	34.26 (39.47)	5.43 (0.86)
FINGESTURE	494	23.68	overall	61 (9.96)	97.84 (31.86)	4.53 (0.34)	n.a.	n.a.	525.4 (885.4)	5.83 (0.84)
			women	64 (9.56)	87.88 (32.36)	4.41 (0.36)	n.a.	n.a.	630.8 (1400.1)	5.85 (0.89)
			men	60 (9.89)	100.72 (31.17)	4.58 (0.32)	n.a.	n.a.	493.7 (661.1)	5.82 (0.83)
FLEMENGHO -EPOGH	196	48.98	overall	52.74 (11.44)	n.a.	n.a.	n.a.	n.a.	n.a.	3.07 (0.40)
			women	53.00 (10.54)	n.a.	n.a.	n.a.	n.a.	n.a.	3.02 (0.37)
			men	52.50 (12.30)	n.a.	n.a.	n.a.	n.a.	n.a.	3.11 (0.41)
GenR	392	49.49	overall	1.22 (0.08)	n.a.	n.a.	n.a.	n.a.	n.a.	2.85 (0.42)
			women	1.22 (0.08)	n.a.	n.a.	n.a.	n.a.	n.a.	2.86 (0.44)
			men	1.22 (0.07)	n.a.	n.a.	n.a.	n.a.	n.a.	2.84 (0.38)
GTR	134	100	overall	23.43 (3.39)	51.05 (20.96)	3.86 (0.39)	37.17 (25.04)	3.45 (0.55)	1034.68 (1684.47)	6.29 (1.1)
			women	23.43 (3.39)	51.05 (20.96)	3.86 (0.39)	37.17 (25.04)	3.45 (0.55)	1034.68 (1684.47)	6.29 (1.1)
			men	n.a.	n.a.	n.a.	n.a.	n.a.	n.a.	n.a.
KORA S4	1.617	51.64	overall	53.46 (8.82)	35.93 (19.15)	3.47 (0.46)	25.26 (19.11)	3.04 (0.61)	61.20 (215.59)	3.25 (1.25)
			women	53.26 (8.77)	35.36 (19.34)	3.46 (0.46)	25.79 (19.32)	3.06 (0.60)	61.43 (106.10)	3.37 (1.24)
			men	53.68 (8.87)	36.54 (18.94)	3.49 (0.46)	24.69 (18.89)	3.01 (0.61)	60.96 (290.09)	3.12 (1.26)
MESA	2.401	52.35	overall	62.3 (10.1)	33.6 (16.4)	2.9 (0.6)	25.0 (22.5)	3.0 (0.66)	n.a.	n.a.
			women	62.3 (10.2)	22.5 (14.5)	2.9 (0.6)	25.3 (19.5)	3.0 (0.64)	n.a.	n.a.
			men	62.4 (9.9)	22.6 (18.1)	2.9 (0.6)	24.7 (25.4)	2.9 (0.67)	n.a.	n.a.
MRS	1.383	0	overall	22.75 (3.48)	65.43 (27.43)	4.10 (0.41)	59.51 (34.98)	3.94 (0.54)	4304.72 (5277.67)	7.86 (1.04)
			women	n.a.	n.a.	n.a.	n.a.	n.a.	n.a.	n.a.
			men	22.75 (3.48)	65.43 (27.43)	4.10 (0.41)	59.51 (34.98)	3.94 (0.54)	4304.72 (5277.67)	7.86 (1.04)
NESDA	1.740	68.05	overall	41.88 (12.59)	61.19 (25.20)	4.11 (3.23)	33.14 (20.66)	3.50 (3.03)	42.95 (24.23)	1.57 (0.24)
			women	40.95 (12.69)	60.31 (24.43)	4.10 (3.20)	33.96 (20.83)	3.53 (3.04)	45.65 (24.60)	1.60 (0.23)
			men	43.86 (12.15)	63.07 (26.71)	4.14 (3.29)	31.38 (20.20)	3.45 (3.01)	37.19 (22.35)	1.50 (0.25)

NTR	439	65.15	overall	30.83 (14.42)	77.09 (32.75)	4.21 (0.46)	61.02 (34.41)	3.97 (0.55)	76.73 (46.56)	4.18 (0.58)
			women	29.84 (14.71)	60.65 (34.16)	3.98 (0.54)	75.22 (31.28)	4.24 (0.44)	79.52 (45.94)	4.23 (0.57)
			men	32.67 (7.84)	81.28 (35.47)	4.30 (0.50)	61.80 (41.20)	3.97 (0.59)	71.51 (47.41)	4.09 (0.62)
PIVUS	766	53.66	overall	70.20 (0.18)	34.56 (18.19)	3.43 (0.46)	n.a.	n.a.	243.20 (385.89)	4.79 (1.20)
			women	70.27 (0.15)	34.60 (18.61)	3.44 (0.45)	n.a.	n.a.	240.6 (335.90)	4.84 (1.17)
			men	70.13 (0.18)	34.52 (17.73)	3.43 (0.47)	n.a.	n.a.	246.2 (437.10)	4.73 (1.24)
PREVEND	2.793	49.48	overall	53.22 (11.68)	35.64 (17.46)	3.47 (0.45)	29.35 (19.70)	3.24 (0.51)	438.32 (1089.07)	5.39 (1.13)
			women	53.90 (12.06)	36.15 (18.19)	3.48 (0.47)	28.11 (19.76)	3.19 (0.51)	398.76 (1109.34)	5.27 (1.13)
			men	52.55 (11.25)	35.14 (16.71)	3.46 (0.44)	30.57 (19.58)	3.28 (0.51)	477.12 (1067.78)	5.50 (1.12)
RS1	972	37.55	overall	79.13 (4.79)	36.70 (31.39)	3.37 (0.64)	35.66 (45.39)	3.13 (0.86)	n.a.	n.a.
			women	78.80 (4.47)	43.07 (39.77)	3.48 (0.71)	43.30 (57.93)	3.23 (3.23)	n.a.	n.a.
			men	79.23 (4.97)	79.23 (4.97)	3.31 (0.59)	31.06 (35.05)	5.66 (3.08)	n.a.	n.a.
RS2	985	58.78	overall	72.13 (5.00)	35.10 (27.72)	3.38 (0.56)	31.91 (45.54)	3.07 (0.77)	n.a.	n.a.
			women	72.25 (5.18)	32.87 (25.68)	3.33 (0.52)	29.49 (44.97)	3.03 (0.72)	n.a.	n.a.
			men	71.96 (4.71)	38.27 (30.15)	3.44 (0.60)	35.34 (46.18)	3.12 (0.85)	n.a.	n.a.
TRAILS-CC	307	30.62	overall	11.11 (0.48)	75.79 (38.05)	6.51 (0.50)	89.21 (54.25)	4.31 (0.62)	4277.19 (5828.07)	7.64 (1.27)
			women	11.07 (0.55)	73.52 (30.44)	6.51 (0.44)	82.15 (42.75)	4.26 (0.59)	3509.59 (3736.52)	7.60 (1.16)
			men	11.13 (0.45)	76.79 (40.98)	6.51 (0.52)	92.31 (58.41)	4.33 (0.64)	4619.12 (6526.94)	7.65 (1.31)
TRAILS-Pop	1.222	52.7	overall	11.46 (1.57)	70.74 (35.84)	4.13 (0.51)	77.61 (51.06)	4.14 (0.68)	3715.66 (5230.48)	7.47 (1.32)
			women	11.42 (1.54)	67.49 (33.42)	4.09 (0.49)	73.04 (48.02)	4.09 (0.66)	3327.63 (4981.83)	7.36 (1.30)
			men	11.50 (1.60)	74.34 (38.05)	4.18 (0.53)	82.69 (53.83)	4.20 (0.69)	4145.51 (5465.12)	7.58 (1.35)
ULSAM	67	0	overall	71.05 (0.46)	163.81 (52.87)	5.05 (0.31)	n.a.	n.a.	11.37 (6.60)	2.25 (0.54)
			women	n.a.	n.a.	n.a.	n.a.	n.a.	n.a.	n.a.
			men	71.05 (0.46)	163.81 (52.87)	5.05 (0.31)	n.a.	n.a.	11.37 (6.60)	2.25 (0.54)
YFS	1.827	55.12	overall	31.68 (4.99)	50.76 (23.36)	3.83 (0.44)	48.48 (32.34)	3.69 (0.62)	1010 (1497)	6.26 (1.18)
			women	31.68 (4.98)	50.6 (24.35)	3.82 (0.46)	50.05 (34.74)	3.70 (0.65)	1161 (1759)	6.37 (1.23)
			men	31.68 (4.99)	50.97 (22.1)	3.84 (0.42)	46.56 (29.03)	3.67 (0.58)	823.8 (1063)	6.13 (1.11)
**Stage 1 discovery total**	**28.700**									
**STAGE 2 REPLICATION COHORTS**										
CARLA	1.367	47.7	overall	62.74 (9.77)	30.55 (15.89)	3.30 (0.48)	23.25 (19.14)	2.94 (0.61)	291.60 (557.22)	4.88 (1.22)
			women	62.56 (9.59)	31.61 (16.18)	3.35 (0.45)	24.94 (19.35)	3.03 (0.58)	343.60 (623.83)	5.14 (1.14)
			men	62.91 (9.93)	29.58 (15.57)	3.27 (0.49)	21.70 (18.82)	2.86 (0.62)	244.00 (484.18)	4.65 (1.25)
FINCAVAS	542	44.83	overall	52.90 (12.94)	28.96 (15.35)	3.22 (0.57)	20.95 (12.15)	2.89 (0.56)	102.48 (190.76)	3.80 (1.29)
			women	54.51 (11.94)	27.29 (14.05)	3.16 (0.56)	19.98 (11.07)	2.85 (0.55)	91.89 (137.72)	3.76 (1.27)
			men	51.59 (13.57)	30.31 (16.23)	3.26 (0.58)	21.74 (12.92)	2.92 (0.57)	111.09 (224.71)	3.83 (1.32)
KORA S4	1.959	50.89	overall	44.98 (15.85)	42.40 (23.38)	3.62 (0.51)	33.76 (23.98)	3.30 (0.68)	105.48 (191.06)	3.80 (1.41)
			women	44.21 (15.41)	42.85 (24.47)	3.63 (0.51)	35.37 (25.40)	3.35 (0.67)	117.80 (220.36)	3.94 (1.37)
			men	45.78 (16.26)	41.94 (22.20)	3.61 (0.52)	32.09 (22.30)	3.25 (0.68)	92.71 (154.03)	3.65 (1.43)

Lifelines	12.101	58.59	overall	48.01 (11.14)	33.42 (24.22)	3.29 (0.66)	33.41 (28.13)	3.26 (0.71)	n.a.	n.a.
			women	48.00 (11.10)	33.84 (24.22)	3.31 (0.65)	34.87 (29.06)	3.30 (0.70)	n.a.	n.a.
			men	48.00 (11.21)	32.84 (24.22)	3.27 (0.68)	31.35 (26.62)	3.19 (0.72)	n.a.	n.a.
MRC NSHD	1.127	55.72	overall	63.3 (1.0)	32.3 (13.1)	3.4 (0.4)	21.2 (12.2)	2.9 (0.5)	227.3 (1.3)	4.7 (1.0)
			women	63.4 (1.0)	31.7 (12.3)	3.4 (0.4)	21.8 (12.2)	2.9 (0.5)	240.0 (4.7)	4.8 (1.0)
			men	63.3 (1.1)	33.1 (14.0)	3.4 (0.4)	20.5 (12.1)	2.9 (0.5)	162.9 (207.8)	4.6 (1.0)
NESDA	606	64.36	overall	42.33 (14.32)	77.01 (34.27)	4.25 (0.43)	45.34 (35.24)	3.57 (0.68)	47.78 (32.08)	3.68 (0.64)
			women	42.06 (14.17)	75.41 (31.94)	4.24 (0.42)	45.12 (33.08)	3.59 (0.65)	50.38 (32.43)	3.76 (0.60)
			men	42.82 (14.62)	79.90 (38.02)	4.28 (0.45)	45.76 (38.92)	3.54 (0.74)	43.08 (30.95)	3.54 (0.69)
NFBC 1966	1.941	54.97	overall	46.6 (0.6)	32.5 (14.3)	3.4 (0.4)	n.a.	n.a.	264.6 (407.9)	4.8 (1.3)
			women	46.6 (0.6)	32.3 (13.5)	3.4 (0.4)	n.a.	n.a.	304.8 (447.2)	5.0 (1.3)
			men	46.6 (0.6)	32.9 (15.3)	3.4 (0.5)	n.a.	n.a.	215.6 (348.2)	4.6 (1.3)
UCSD TWINS	230	76.52	overall	41.03 (16.53)	61.89 (32.41)	3.98 (0.6)	n.a.	n.a.	152.16 (364.59)	3.96 (1.60)
			women	41.27 (16.38)	61.52 (33.14)	3.98 (0.6)	n.a.	n.a.	165.10 (407.87)	4.02 (1.59)
			men	38.78 (16.96)	63.07 (30.18)	4.00 (0.63)	n.a.	n.a.	110.01 (150.62)	3.77 (1.60)
WHI CT–GARNET (controls)	1.648	100	overall	65.26 (6.77)	19.73 (15.63)	2.76 (0.65)	21.09 (19.74)	2.79 (0.68)	n.a.	n.a.
			women	65.26 (6.77)	19.73 (15.63)	2.76 (0.65)	21.09 (19.74)	2.79 (0.68)	n.a.	n.a.
			men	n.a.	n.a.	n.a.	n.a.	n.a.	n.a.	n.a.
WHI CT–MOPMAP (controls)	1.198	100	overall	63.11 (6.61)	20.29 (15.93)	2.79 (0.65)	22.26 (20.86)	2.84 (0.69)	n.a.	n.a.
			women	63.11 (6.61)	20.29 (15.93)	2.79 (0.65)	22.26 (20.86)	2.84 (0.69)	n.a.	n.a.
			men	n.a.	n.a.	n.a.	n.a.	n.a.	n.a.	n.a.
WHII	1.755	24.22	overall	60.30 (5.85)	38.73 (27.04)	3.53 (0.47)	n.a.	n.a.	382.48 (2618.92)	4.76 (1.20)
			women	60.64 (5.83)	40.81 (28.20)	3.52 (0.46)	n.a.	n.a.	484.38 (2851.76)	5.02 (1.22)
			men	60.19 (5.83)	38.06 (25.19)	3.56 (0.48)	n.a.	n.a.	349.92 (2697.58)	4.67 (1.19)
**Stage 2 replication total**	**24.474**									
**STAGE 3 LOOKUP COHORTS OF OTHER ETHNICITY**										
HCHS/SOL	10.830	59.41	overall	45.18 (13.61)	29.75 (22.53)	3.16 (0.69)	35.79 (29.25)	3.31 (0.73)	n.a.	n.a.
			women	45.91 (13.41)	29.89 (22.03)	3.18 (0.67)	36.98 (29.56)	3.36 (0.72)	n.a.	n.a.
			men	44.11 (13.83)	29.55 (23.23)	3.14 (0.71)	34.04 (28.70)	3.25 (0.75)	n.a.	n.a.
MRS	404	0	overall	22.51 (3.12)	62.90 (24.84)	4.06 (0.40)	58.57 (32.98)	3.93 (0.52)	4069.94 (4706.06)	7.82 (1.02)
			women	n.a.	n.a.	n.a.	n.a.	n.a.	n.a.	n.a.
			men	22.51 (3.12)	62.90 (24.84)	4.06 (0.40)	58.57 (32.98)	3.93 (0.52)	4069.94 (4706.06)	7.82 (1.02)
**Total HIS**		**11.234**								
ARIC	1.582	63.15	overall	53.06 (5.76)	38.00 (19.77)	3.5 (0.52)	34.20 (24.54)	3.33 (0.63)	22.84 (37.44)	2.38 (1.31)
			women	52.94 (5.63)	36.93 (19.10)	3.48 (0.52)	34.51 (24.38)	3.34 (0.64)	23.98 (37.10)	2.45 (1.30)
			men	53.28 (5.98)	39.84 (20.77)	3.56 (0.51)	33.67 (24.82)	3.32 (0.62)	20.90 (38.00)	2.28 (1.33)
HANDLS	188	54.79	overall	47.32 (8.90)	32.79 (13.91)	3.40 (0.43)	34.04 (13.67)	3.45 (0.41)	483.87 (604.08)	5.68 (1.01)
			women	47.59 (9.17)	32.56 (12.55)	3.41 (0.40)	35.96 (13.79)	3.51 (0.92)	541.57 (566.36)	5.86 (0.97)
			men	47.04 (8.61)	33.06 (15.46)	3.39 (0.47)	31.68 (13.23)	3.37 (0.41)	413.94 (643.31)	5.47 (1.02)

MESA	1.480	54.73	overall	61.9 (10.0)	26.72 (19.78)	3.08 (0.64)	32.72 (27.80)	3.24 (0.70)	n.a.	n.a.
			women	61.84 (9.95)	26.71 (18.53)	3.09 (0.62)	33.33 (25.74)	3.27 (0.68)	n.a.	n.a.
			men	61.95 (10.14)	26.74 (20.99)	3.07 (0.65)	31.99 (30.11)	3.20 (0.72)	n.a.	n.a.
MRS	131	0	overall	23.74 (4.73)	59.83 (22.94)	4.03 (0.37)	58.76 (28.53)	3.96 (0.48)	3804.05 (4239.97)	7.87 (0.91)
			women	n.a.	n.a.	n.a.	n.a.	n.a.	n.a.	n.a.
			men	23.74 (4.73)	59.83 (22.94)	4.03 (0.37)	58.76 (28.53)	3.96 (0.48)	3804.05 (4239.97)	7.87 (0.91)
WHI CT - SHARe	3.518	100	overall	60.84 (6.70)	22.03 (17.67)	2.86 (0.68)	26.79 (24.92)	3.01 (0.73)	n.a.	n.a.
			women	60.84 (6.70)	22.03 (17.67)	2.86 (0.68)	26.79 (24.92)	3.01 (0.73)	n.a.	n.a.
			men	n.a.	n.a.	n.a.	n.a.	n.a.	n.a.	n.a.
**Total AfAm**		**6.899**								
**Stage 3 other ethnicity total**		**18.133**								
**POST-GWAS COHORTS**										
ABCD	1.094	0.51	overall	5.51 (0.38)	73.10 (30.95)	4.2 (0.43)	80.87 (45.91)	4.23 (0.59)	121.39 (62.42)	4.66 (0.54)
			women	5.51 (0.39)	72.42 (30.95)	4.19 (0.43)	79.22 (46.19)	4.21 (0.59)	120.72 (62.65)	4.66 (0.53)
			men	5.50 (0.37)	73.80 (30.96)	4.21 (0.43)	82.58 (45.60)	4.25 (0.60)	122.09 (62.23)	4.67 (0.55)
OFS	1.326	56.03	overall	33.41 (15.73)	61.92 (32.46)	4.02 (0.45)	41.39 (22.66)	3.57 (0.56)	197.43 (2569.48)	10.58 (1.49)
			women	33.98 (14.90)	55.27 (29.54)	3.91 (0.44)	39.34 (23.34)	3.50 (0.59)	97.78 (233.66)	10.35 (1.54)
			men	32.68 (16.73)	70.24 (34.01)	4.16 (0.43)	43.96 (21.53)	3.67 (0.51)	318.79 (3816.42)	10.87 (1.38)

SD: standard deviation; n.a.: not available.

Supplementary Table 4: Technical information on the genotyping and imputation method of stage 1 discovery, stage 2 replication, stage 3 other ethnicity lookup, and post-GWAS cohorts used in the V_gHRV analyses.

Study acronym	Array(s)	Calling Algorithm	QC filters for exclusion of genotyped SNPs	QC filters for exclusion of individuals	Imputation software	Imputation reference panel (build)	Total SNPs after imputation	Statistical software	Genomic control lambda SDNN / RMSSD / pvRSA/HF
**STAGE 1 DISCOVERY COHORTS**									
ARIC	Affymetrix GeneChip SNP Array 6.0	Birdseed	call rate ≤ 95%; MAF <1%; pHWE< 1E-6	call rate <95%	MaCH v1.0.16	HapMap Phase II data in the CEU individuals release 22 (build36)	2.543.887	Probabel	1.015 / 1.011 / 1.012
CHS	Illumina 370CNV	BeadStudio	call rate <97%; MAF: n.a. - excluded SNPs with 0 heterozygotes; pHWE< 1E-5	call rate<=95% or if their genotype was discordant with known sex or prior genotyping	BIMBAM v0.99	HapMap Phase II data in the CEU individuals release 22 (build36)	2.318.082	R	1.011 / 1.009 / 1.016
FHS	Affymetrix 500K, Affymetrix 50K supplemental	BRLMM	call rate ≤ 97%; MAF: n.a.; pHWE< 1E-6	call rate <90%	Mach1 v1.0.15	HapMap Phase II data in the CEU individuals release 22 (build36)	2.466.720	PLINK, R	1.012 / 1.010 / 1.014
FINGESTURE	Affymetrix 6.0	Birdseed (as implemented in Affymetrix powertools 1.10.2 package)	call rate < 95%; MAF < 0.01; pHWE: n.a.	Sample genotyping rate ≤97%	MACH 1.0.15	HapMap Phase II data in the CEU individuals release 22 (build36)	2.543.888	MACH2QTL V1.0.4	0.997/ - / 1.006
FLEMENGHO-EPOGH	ILLUMINA 1M	ILLUMINA Genome Studio	call rate< 99%; MAF <0.01; pHWE< 1E-08	call rate <99%; sex mismatch; heterozygosity > 4SD from mean; non-Caucasians	MACH	HapMap Phase II data in the CEU individuals release 22 (build36)	2.581.944	PLINK, R	- / - / 1.003
GenR	Ilumina 610 Quad array	Genomestudio 2009 V.1.1.9	call rate <95%; MAF <1%; pHWE< 1E-6	Call rate <97,50%; Heterozygosity > 4SD from mean; Caucasian only, ethnic outliers excluded based on PCA	MACH	HapMap Phase II data in the CEU individuals release 22 (build36)	25.438.877	PLINK	- / - / 1.007

GTR	Illumina Cyto SNP12 v2	GenomeStudio	call rate < 95%; MAF <1%; pHWE< 1E-4	call rate <95%; sex mismatch; heterozygosity > 4SD from mean; non-Caucasians	IMPUTE v2	HapMap Phase II data in the CEU individuals release 22 (build36)	2.631.501	SNPTEST v2.2.0	1.013 / 1.022 / 1.017
KORA S4	Affymetrix 6.0	Birdseed v2	call rate< 93%; HapMap SNPs only	call rate< 93%; sex mismatch	MACH v1.0.15	HapMap Phase II data in the CEU individuals release 22 (build36)	2.543.887	MACH2QTL v1.0.8	1.022 / 1.012 / 1.007
MESA	Affymetrix 6.0	Birdseed v2	call rate < 95%; MAF: n.a.; pHWE: n.a; heterozygosity > 53%.	sex mismatches, expected and unexpected duplicates, call rate < 95%	IMPUTE v2.1.0	HapMap Phase I and II CEU individuals release 24 (build36)	3.854.661	R	0.994 / 0.998 / -
MRS	Illumina HumanOmniExpressExome array (HOEE 12v1.0)	Genome Studio	call rate< 95%; MAF: n.a.; pHWE< 5E-08	call rate <98%; heterozygosity <0.211 or >0.302; non-Caucasians	IMPUTEv2	1000Genomes global panel phase 1 release v3 (build 37)	32.179.008	SNPTEST v2.4.1	0.999 / 1.008 / 1.003
NESDA	Perlegen-Affymetrix 5.0; Affymetrix 6.0 907K	Proprietary	call rate ≤ 95%; MAF <0.01; pHWE: n.a.	Non-Caucasians, XO and XXY samples, and samples with a call rate <95%, high genome-wide homo- or heterozygosity, excess IBS	IMPUTE	HapMap Phase II data in the CEU individuals release 22 (build36)	2.135.543	SNPTEST v1.1.4	1.008 / 1.000 / 0.998
NTR	Affymetrix-Perlegen; Illumina 370, 660, 1M Omi; Affymetrix 6.0	Proprietary Affymetrix Calling Algorithms, Birdsuite 1 & 2.	callrate SNPs < 90%, MAF < 0.01, HWE < 0.00001	Callrate < 90%, Sex /IBD mismatch, F(st) > 0.10 or F < 0.10, Ethnicity based on PCs (deviating from maximum European PC values), Mendelian 2 error rate in relation to family member > 2%	IMPUTE	HapMap Phase II CEU B36 rel 24.	2.401.535	SNPTEST v1.1.4	0.998 / 1.002 / 1.015

PIVUS	Illumina OmniExpress + Metabochip	Proprietary	call rate< 95%; MAF< 0.01; pHWE< 1E-6	Call rate <95%; heterozygosity, gender check and relatedness	IMPUTE	HapMap Phase II data in the CEU individuals release 22 (build36)	2.616.874	PLINK	1.004 / - / 1.006
PREVEND	Illumina HumanCytoSNP12 v2 beadchip assay	Illumina Genome Studio software	call rate < 0.95; MAF <0.01; pHWE <E-5	call rate < 0.95; sex mismatch; non-Caucasians	BEAGLE v3.3.1	HapMap Phase II data in the CEU individuals release 22 (build36)	2.269.099	PLINK	1.014 / 1.000 / 1.001
RS1	Illumina, HumanHap550 - chip v3.0	BeadStudio	call rate < 98%; MAF <1%; pHWE< 1E-6	excess autosomal heterozygosity, sex mismatch or outlying identity-by-state clustering estimates	Mach1 v1.0.15	HapMap Phase II data in the CEU individuals release 22 (build36)	2.543.887	ProbABEL,R	1.006 / 1.011 / -
RS2	Illumina550K Duo, 610KQuad	GenomeStudio	call rate < 98%; MAF <1%; pHWE< 1E-6	excess autosomal heterozygosity, sex mismatch or outlying identity-by-state clustering estimates	Machv1.0.16	HapMap Phase II data in the CEU individuals release 22 (build36)	2.543.887	ProbABEL, R	0.995 / 1.007 / -
TRAILS-CC	Illumina Cyto SNP12 v2	GenomeStudio	call rate < 95%; MAF <1%; pHWE< 1E-4	call rate <95%; sex mismatch; heterozygosity > 4SD from mean; non-Caucasians	IMPUTE v2	HapMap Phase II data in the CEU individuals release 22 (build36)	2.631.501	SNPTEST v2.2.0	1.014 / 1.012 / 1.011
TRAILS-Pop	Illumina Cyto SNP12 v2	GenomeStudio	call rate < 95%; MAF <1%; pHWE< 1E-4	call rate <95%; sex mismatch; heterozygosity > 4SD from mean; non-Caucasians	IMPUTE v2	HapMap Phase II data in the CEU individuals release 22 (build36)	2.631.501	SNPTEST v2.2.0	0.994 / 0.994 / 0.992
ULSAM	Illumina OMNI2.5 + Metabochip	Proprietary	call rate< 99%%; MAF <0.01; pHWE: n.a.	Call rate <95%; heterozygosity, gender check and relatedness	IMPUTE v2	HapMap Phase II data in the CEU individuals release 22 (build36)	3.126.254	PLINK v1.07	1.005 / - / 1.007
YFS	Illumina 670k custom	Illuminus	call rate ≤ 95%; MAF<0.01; pHWE< 1E-6	Call rate <95%; heterozygosity, gender check and relatedness	MACH 1.0	HapMap Phase II data in the CEU individuals release 22 (build36)	2.543.887	ProbABEL v0.1-3	1.006 / 1.013 / 1.015

**STAGE 2 REPLICATION COHORTS**									
CARLA	TaqMan® (Applied Biosystems, Darmstadt, Germany)	Sequence Detection Software SDS 2.3	call rate< 95%; MAF <0.01; pHWE: n.a.	non-Caucasians	n.a.	n.a.	n.a.	R	-
FINCAVAS	Metabochip	GenomeStudio	call rate ≤ 95%; MAF <0.01; pHWE< 1E-6	Call rate <95%; heterozygosity, gender check and relatedness	SHAPEIT v2 / IMPUTE 2.3.0	1000Genomes global panel phase 1 release v3 (build 37)	38.943.535	SNPTEST v2.5	-
KORA S4	Affymetrix Axiom	Affymetrix Software	call rate ≤ 98%; MAF: <1%; pHWE< 1E-6	call rate ≤ 97%; sex mismatch; heterozygosity >5SD from mean; non-Caucasian; population outliers; participant in stage 1	SHAPEIT v2, IMPUTE v2.3.0	1000Genomes global panel phase 1 release v3 (build 37)	30.067.091	R	-
Lifelines	Illumina Cyto SNP12 v2	GenomeStudio	call rate < 98%; MAF <1%; pHWE< 1E-5	call rate <95%; sex mismatch; heterozygosity > 4SD from mean; non-Caucasians	MACH minimac	1000Genomes global panel phase 1 release v3 (build 37)	28.681.763	PLINK v1.07	-
MRC NSHD	LGC KASP	Proprietary	call rate < 90%; MAF <1%; pHWE< 0.05	call rate < 90%,	n.a.	n.a.	n.a.	SAS	-
NESDA	Affymetrix 6.0 907K	Birdseed	call rate< 95%; MAF <0.01; pHWE<1E-5.; unmapped; allele frequency difference with reference >20%; ambiguous SNPs with allele frequency>35%	call rate<90%; heterozygosity abs(PLINK F)>0.1; sex mismatch; unexpected relatedness	Minimac	1000Genomes global panel phase 1 release v3 (build 37)	3.000.779	SNPTEST v2.4.1	-

NFBC 1966	Illumina HumanCNV-370DUO Analysis BeadChip	Beadstudio	call rate < 95%; MAF <1%; pHWE< 1E-07	call rate <95%	IMPUTE v2	HapMap Phase II data in the CEU individuals release 22 (build36)	3.855.963	SNPTEST 2.4.1	-
UCSD TWINS	Illumina 610 Quad genotyping array	Proprietary	call rate< 95%; MAF <0.01; pHWE: n.a.	call rate <95%	MaCH	HapMap Phase II data in the CEU individuals release 22 (build36)	2.587.522	PLINK	-
WHI – GARNET	Human Omni1-Quad v1-0 B	BeadStudio v3.1.3.0	call rate ≤ 98%; MAF: n.a.; pHWE< 1E-4	call rate ≤ 98%	BEAGLE v3.3.1	1000Genomes global panel phase 1 release v3 (build 37)	8.905.697	lm.R	-
WHI – MOPMAP	Affymetrix Axiom Genome-Wide Human CEU I	Birdseed	call rate ≤ 90%; MAF <0.5%; pHWE< 1E-06	call rate ≤ 95%	MaCH minimac	HapMap Phase II data in the CEU individuals release 22 (build36)	2.543.886	lm.R	-
WHII	Illumina Metabochip	GenomeStudio	call rate< 0.95	call rate< 0.95, sex mismatch, duplicates	Minimac	1000 Genomes global panel phase 1 release v3 (build 37)	1.217.802	PLINK	-
**STAGE 3 LOOKUP COHORTS OF OTHER ETHNICITY**									
HCHS/SOL	Illumina HumanOmni 2.5-8v1-1 + custom content	GenomeStudio v2011.1	call rate: 98%; MAF: n.a.; pHWE< 1E-05	sex mismatch; unexpected duplicates	IMPUTEv2	1000Genomes global panel phase 1 release v3 (build 37)	25.568.744	R/Bioconductor GENESIS package	-
MRS	Illumina HumanOmni ExpressExome array (HOEE 12v1.0)	Genome Studio	call rate: 95%; MAF: n.a.; pHWE< 5E-08	call rate <98%; heterozygosity <0.211 or >0.302; non-Hispanics	IMPUTEv2	1000Genomes global panel phase 1 release v3 (build 37)	32.179.008	SNPTEST v2.4.1	-

ARIC	Affymetrix GeneChip SNP Array 6.0	Birdseed	call rate ≤ 90%; MAF <1%; pHWE: n.a.	call rate <95%	MaCH v1.0.16	HapMap Phase II data in the YRI+CEU individuals release 22 (build36)	2.653.878	Probabel	-
HANDLS	Illumina genotyping arrays including 1M and 1Mduo	Illumina BeadStudio	Genotype call rate <95%; HWE p-value < 1.0E-7; MAF < 0.01;	Sample call rate < 95%; excess heterozygosity > 3SD from the mean; Ethnic outliers, Cryptic relatedness; Sex mismatch	MACH/minimac	HapMap Phase II data in the YRI+CEU individuals release 22 (build36)	2.939.993	MACH2QTLv1.08	-
MESA	Affymetrix 6.0	Birdseed v2	call rate < 95%; MAF: n.a.; pHWE: n.a; heterozygosity > 53%.	sex mismatches, expected and unexpected duplicates, call rate < 95%	IMPUTE v2.2.2	1000Genomes global panel phase 1 release v3 (build 37)	39.295.080	R	-
MRS	Illumina HumanOmniExpressExome array (HOEE 12v1.0)	Genome Studio	call rate ≤ 95%; MAF: n.a.; pHWE< 5E-08	call rate <98%; heterozygosity <0.211 or >0.302; non-African-Americans	IMPUTEv2	1000Genomes global panel phase 1 release v3 (build 37)	32.179.008	SNPTEST v2.4.1	-
WHI – SHARe	Affymetrix GeneChip SNP Array 6.0	Birdseed	call rate ≤ 95%; MAF <1%; pHWE< 1E-06	call rate ≤ 95%	MaCH v1.0.16	HapMap Phase II data in the YRI+CEU individuals release 22 (build36)	2.203.608	lm.R	-
**POST-GWAS COHORTS**									
ABCD	Illumina HumanCoreExomeChip	GenomeStudio	call rate ≤ 95%; MAF <1%; pHWE< 1E-06	Call rate <95%; heterozygosity, gender check and relatedness	IMPUTE v2	1000Genomes global panel phase 1 release v3 (build 37)	27.448.454	GTOOL; PLINK	-

MAF: minor allele frequency; HWE: Hardy-Weinberg Equilibrium

Supplementary Table 5: Conditional analyses of the SNPs on chromosomes 14 and 15 for confirmation of independent secondary signals within these loci using (a) individual data from Lifelines Cohort Study (n=12,101) and (b) using the joint-and-conditional analysis in the Genome-wide Complex Trait Analysis software package of the stage 1 summary statistics.

**(a)**		**conditioned on**		**SDNN**		**RMSSD**	
**SNP**	**locus**	**SNP**	**locus**	**PCondLL**	**POrigLL**	**PCondLL**	**POrigLL**
rs2052015	14b	rs4899412	14b	1.2E-02	4.7E-05	2.1E-02	1.2E-04
rs2529471	14c	rs4899412	14b	2.5E-05	4.7E-06	2.3E-05	4.6E-06
rs36423	14a	rs4899412	14b	2.1E-03	1.3E-04	2.3E-02	2.4E-03
rs4899412	14b	rs2052015	14b	8.5E-01	1.3E-03	7.8E-01	1.9E-03
rs2529471	14c	rs2052015	14b	6.1E-05	4.7E-06	5.2E-05	4.6E-06
rs36423	14a	rs2052015	14b	1.4E-02	1.3E-04	9.0E-02	2.4E-03
rs4899412	14b	rs2529471	14c	7.8E-03	1.3E-03	1.1E-02	1.9E-03
rs2052015	14b	rs2529471	14c	6.3E-04	4.7E-05	1.4E-03	1.2E-04
rs36423	14a	rs2529471	14c	7.8E-04	1.3E-04	1.0E-02	2.4E-03
rs4899412	14b	rs36423	14a	2.4E-02	1.3E-03	1.8E-02	1.9E-03
rs2052015	14b	rs36423	14a	4.7E-03	4.7E-05	3.5E-03	1.2E-04
rs2529471	14c	rs36423	14a	2.7E-05	4.7E-06	1.8E-05	4.6E-06
rs2680344	15a	rs1812835	15b	4.0E-02	4.0E-03	1.9E-02	9.1E-04
rs1812835	15b	rs2680344	15a	2.7E-02	2.8E-03	8.7E-03	4.3E-04

PCondLL=*p*-value of the SNP in Lifelines when conditioned on other SNP; POrigLL=original *p*-value of the SNP in Lifelines when not conditioned on any other SNP.

**(b)**		**conditional p-value GCTA**	
**SNP**	**locus**	**SDNN**	**RMSSD**
rs4899412	14b	3.1E-04	9.4E-03
rs2052015	14b	2.0E-03	4.6E-09[a]
rs2529471	14c	9.0E-07[b]	4.1E-05
rs36423	14a	1.0E-07[b]	2.7E-03
rs2680344	15a	1.1E-07[c]	7.6E-04
rs1812835	15b	1.3E-03	6.6E-07[d]

[a] rs2052015 was identified as the independent SNP associated with RMSSD in the locus on chromosome 14. The *p*-value given for this SNP is the unconditioned one. The *p*-values for the other three SNPs in this locus are *p*-values conditioned on rs2052015.
[b] rs2529471 and rs36423 were identified as independent SNP associated with SDNN in the locus on chromosome 14. The *p*-values given for these two SNPs are the *p*-values from the model that contained both SNPs. The *p*-values for the other two SNPs in this locus are *p*-values conditioned on rs2529471 and rs36423.
[c] rs2680344 was identified as independent SNP associated with SDNN in the locus on chromosome 15. The *p*-value given for this SNP is the unconditioned one. The *p*-value for the other SNP in this locus is the *p*-value conditioned on rs2680344.
[d] rs1812835 was identified as independent SNP associated with RMSSD in the locus on chromosome 15. The *p*-value given for this SNP is the unconditioned one. The *p*-value for the other SNP in this locus is the *p*-value conditioned on rs1812835.

Supplementary Table 6: Sex-stratified stage 1+2 meta-analysis results for SDNN, RMSSD, and pvRSA/HF for all SNPs that were genome-wide significant ($p<5x10^{-8}/3$) in the overall analysis. SNPs are sorted as in Table 1.

Locus	Chr	SNP	Position (bp) (b36)	Trait	Men					Women				
					N	EAF	β	SE	*p*-value	N	EAF	β	SE	*p*-value
1	19	rs12974991	5845584	RMSSD	18966	0.078	-0.121	0.012	**5.54E-23**	24172	0.078	-0.108	0.011	**2.69E-23**
		rs12974440	5845386	pvRSA/HF†	13625	0.074	-0.246	0.027	**1.87E-20**	15033	0.074	-0.241	0.029	**6.88E-20**
		rs12980262	5844058	SDNN	20270	0.076	-0.065	0.009	**4.79E-13**	25690	0.077	-0.055	0.008	**1.36E-11**
2	12	rs10842383	24663234	SDNN	21606	0.866	-0.048	0.006	**2.98E-14**	26116	0.865	-0.051	0.006	**1.35E-18**
				RMSSD	18979	0.865	-0.060	0.009	**3.90E-12**	24175	0.864	-0.068	0.008	**8.14E-19**
				pvRSA/HF†	14855	0.868	-0.105	0.019	**5.50E-09**	15362	0.869	-0.152	0.019	**1.04E-17**
3	6	rs236349	36928543	SDNN	22771	0.650	-0.036	0.005	**1.17E-14**	28524	0.653	-0.029	0.004	**8.53E-12**
				RMSSD	20144	0.655	-0.040	0.006	**8.13E-10**	26025	0.657	-0.033	0.006	**9.11E-09**
				pvRSA/HF†	16119	0.644	-0.074	0.013	**1.27E-10**	16666	0.648	-0.058	0.014	3.04E-05
4	12	rs7980799	33468257	RMSSD	18979	0.394	-0.041	0.006	**1.40E-10**	25229	0.391	-0.037	0.006	**2.44E-11**
		rs1351682	33490042	pvRSA/HF†	14702	0.436	-0.082	0.013	**5.67E-09**	15074	0.440	-0.063	0.014	1.48E-06
		rs1384598	33514166	SDNN	20223	0.432	-0.027	0.005	**2.64E-08**	27132	0.433	-0.020	0.004	1.81E-06
5	7	rs4262	93389364	SDNN	21606	0.391	-0.028	0.005	**1.81E-08**	27314	0.389	-0.028	0.004	**3.68E-10**
				pvRSA/HF†	14955	0.391	-0.067	0.014	9.44E-08	15457	0.387	-0.042	0.015	6.15E-05
		rs180238	93388383	RMSSD	18979	0.337	-0.037	0.006	**1.77E-08**	25373	0.335	-0.033	0.006	**5.12E-09**
6	14b	rs4899412	71534015	SDNN	20723	0.253	-0.024	0.005	5.80E-06	27443	0.252	-0.027	0.005	**1.54E-08**
		rs2052015	71556806	RMSSD	19526	0.166	-0.033	0.009	1.28E-04	25339	0.164	-0.038	0.008	9.30E-07
	14c	rs2529471	71883022	SDNN	21439	0.428	-0.024	0.005	9.07E-08	28095	0.428	-0.019	0.004	1.83E-06
	14a	rs36423	71422955	SDNN	20652	0.129	-0.032	0.007	4.93E-06	27445	0.130	-0.035	0.006	**1.05E-08**
				RMSSD	19422	0.127	-0.042	0.009	6.88E-06	25373	0.129	-0.039	0.008	2.15E-06
7	15a	rs2680344	71440538	SDNN	22768	0.777	-0.023	0.005	2.64E-05	28517	0.778	-0.025	0.005	4.42E-07
	15b	rs1812835	71294557	RMSSD	18979	0.416	-0.025	0.006	4.51E-05	25374	0.419	-0.026	0.005	1.53E-06
8	20	rs6123471	36273570	RMSSD	20144	0.532	-0.029	0.006	6.45E-06	26025	0.536	-0.019	0.006	5.10E-04

Chr: chromosome; bp: base pair position; N: sample size; EAF: effect allele frequency; β: beta/effect size; SE: standard error of β.
†*p*-value, allele, EAF, N from *p*-value weighted meta-analysis of all cohorts using METAL and β, SE from inverse-variance meta-analysis of only HF cohorts using GWAMA.

Supplementary Table 7: Comparison of the meta-analysis results for the four stage 1 cohorts with ambulatory ECG measurements (CHS, FHS, FINGESTURE, ULSAM) versus those for the other stage 1 cohorts with HRV at rest in a laboratory setting for the 17 genome-wide significantly associated HRV SNPs identified in this study. SNPs are sorted as in Table 1.

Locus	Chr	SNP	Trait	Laboratory rest					Ambulatory					Difference in β's between laboratory rest and ambulatory	
				N	β	SE	*p*-value	I^2	N	β	SE	*p*-value	I^2	*p*-value	I^2
		rs12974991	RMSSD	23646	-0.119	0.011	7.75E-27	40%	2703	-0.094	0.024	1.13E-04	**82%**	3.34E-01	0%
1	19	rs12974440	pvRSA/HF†	20705	-0.241	0.024	7.64E-30	39%	2770	-0.272	0.045	2.73E-09	34%	5.40E-01	0%
		rs12980262	SDNN	24480	-0.055	0.008	2.08E-12	59%	2770	-0.037	0.016	2.46E-02	75%	2.92E-01	10%
			SDNN	24487	-0.055	0.006	4.56E-22	15%	2770	-0.029	0.011	8.62E-03	54%	3.41E-02	**78%**
2	12	rs10842383	RMSSD	23664	-0.074	0.008	9.57E-22	0%	2703	-0.041	0.016	1.25E-02	54%	6.24E-02	71%
			pvRSA/HF†	20705	-0.137	0.017	1.78E-20	48%	2770	-0.100	0.030	1.01E-03	5%	2.79E-01	15%
			SDNN	24487	-0.031	0.004	1.84E-12	17%	2770	-0.017	0.008	4.67E-02	0%	1.25E-01	57%
3	6	rs236349	RMSSD	23662	-0.034	0.007	1.48E-06	9%	2703	-0.013	0.015	3.93E-01	42%	2.05E-01	38%
			pvRSA/HF†	20705	-0.075	0.013	9.61E-11	33%	2770	-0.056	0.024	2.27E-02	0%	4.84E-01	0%
		rs7980799	RMSSD	23453	-0.043	0.006	7.13E-14	0%	2703	-0.026	0.013	4.02E-02	0%	2.20E-01	34%
4	12	rs1351682	pvRSA/HF†	20263	-0.072	0.012	1.19E-09	4%	2770	-0.075	0.023	3.52E-03	0%	8.88E-01	0%
		rs1384598	SDNN	22839	-0.025	0.004	2.97E-08	0%	2770	-0.021	0.008	1.10E-02	9%	7.17E-01	0%
		rs4262	SDNN	24486	-0.030	0.004	9.91E-12	25%	2770	-0.020	0.009	2.71E-02	0%	2.89E-01	11%
5	7		pvRSA/HF†	20704	-0.050	0.012	4.12E-08	14%	2770	-0.045	0.025	7.05E-02	0%	8.55E-01	0%
		rs180238	RMSSD	23663	-0.044	0.006	7.35E-14	25%	2703	-0.016	0.013	2.00E-01	0%	5.28E-02	73%
	14b	rs4899412	SDNN	24485	-0.027	0.005	2.25E-08	46%	2770	-0.014	0.010	1.52E-01	0%	2.55E-01	23%
		rs2052015	RMSSD	23663	-0.046	0.008	1.57E-08	7%	1944	-0.033	0.022	1.32E-01	n.a.	5.77E-01	0%
6	14c	rs2529471	SDNN	24487	-0.021	0.004	6.54E-07	6%	2770	-0.017	0.008	4.17E-02	0%	6.38E-01	0%
	14a	rs36423	SDNN	24482	-0.029	0.006	1.95E-06	54%	2703	-0.045	0.013	7.09E-04	0%	2.62E-01	20%
			RMSSD	23659	-0.042	0.008	4.14E-07	32%	2703	-0.025	0.020	2.13E-01	17%	4.37E-01	0%
7	15a	rs2680344	SDNN	24485	-0.028	0.005	5.39E-08	0%	2770	-0.010	0.010	3.25E-01	0%	1.10E-01	61%
	15b	rs1812835	RMSSD	23664	-0.027	0.006	1.30E-06	0%	2703	-0.016	0.012	1.97E-01	0%	4.12E-01	0%
8	20	rs6123471	RMSSD	23664	-0.024	0.006	3.75E-05	14%	2703	-0.048	0.012	6.49E-05	0%	7.09E-02	69%

Chr: chromosome; bp: base pair position; N: sample size; EAF: effect allele frequency; β: beta/effect size; SE: standard error of β. †*p*-value, allele, EAF, N from *p*-value weighted meta-analysis of all cohorts using METAL and β, SE from inverse-variance meta-analysis of only HF cohorts using GWAMA.

Supplementary Table 8: Analyses of the 17 HRV top SNPs on HRV traits corrected for heart rate.

We performed three corrections: (1) analytical construction of a meta-analysis of the log-transformed coefficients of variation (CV) of SDNN - ln ((SDNN/mean heart period)*100%) and RMSSD- ln ((RMSSD/mean heart period)*100%) using GWIS on the HRV and heart rate GWASs summary statistics; (2) meta-analysis of SNP associations with the log-transformed CV(SDNN) and CV(RMSSD) in Lifelines, NESDA, and TRAILS-Pop; (3) mediation analysis using Sobel's test testing whether heart rate mediates the SNP association with HRV in a meta-analysis of HRV traits and heart rate in LifeLines, NESDA, and TRAILS-Pop. For the mediation analysis for pvRSA/HF only data of NESDA and TRAILS-Pop were available. Significant *p*-values after Bonferroni correction for 11 independent SNPs (<0.0045) are shown in bold.

							GWIS log-transformed CV(SDNN) and CV(RMSSD)		SNP associations log-transformed CV(SDNN) and CV(RMSSD)		Mediation analysis (HRV SNPs -> HR -> HRV traits)			
				HRV		HR p-value			Meta-analysis of three cohorts#		Meta-analysis of three cohorts#			
**Locus**	**Chr**	**HRV SNP**	**HRV Trait**	**β**	***p*-value**		**β (SE)**	***p*-value**	**β (SE)**	***p*-value**	***p*-value**	***p*-value HR corrected**	**%mediated**	**Sobel *p*-value***
1	19	rs12974991	RMSSD	-0.116	**4.6E-46**	1.8E-01	-0.113 (0.010)	**9.5E-29**	-0.129 (0.012)	**6.8E-27**	**9.5E-22**	**4.4E-31**	1.0%	4.8E-01
		rs12974440	pvRSA/HF	-0.244	**1.9E-41**	1.8E-01	n.a.		n.a.		**2.9E-13**	**1.2E-16**	-13.5%	2.9E-01
		rs12980262	SDNN	-0.060	**2.3E-23**	1.8E-01	-0.050 (0.007)	**4.8E-12**	-0.085 (0.011)	**1.3E-15**	**7.3E-14**	**8.1E-16**	4.9%	4.1E-01
2	12	rs10842383	SDNN	-0.049	**9.3E-31**	**1.5E-10**	-0.042 (0.005)	**1.3E-16**	-0.049 (0.008)	**1.3E-09**	**2.0E-11**	**1.7E-08**	26.1%	**3.7E-04**
			RMSSD	-0.065	**2.5E-29**		-0.061 (0.007)	**3.4E-18**	-0.060 (0.009)	**1.8E-10**	**4.5E-11**	**1.8E-08**	30.2%	**3.4E-04**
			pvRSA/HF	-0.124	**1.2E-25**		n.a.		n.a.		**1.3E-04**	5.1E-03	26.8%	**3.1E-03**
3	6	rs236349	SDNN	-0.033	**3.7E-25**	**4.0E-05**	-0.025 (0.004)	**8.6E-10**	-0.031 (0.006)	**7.1E-07**	**2.0E-07**	**1.0E-06**	17.7%	1.4E-01
			RMSSD	-0.035	**9.1E-17**		-0.027 (0.005)	**6.3E-07**	-0.039 (0.007)	**1.3E-07**	**2.8E-07**	**1.9E-07**	17.1%	9.3E-02
			pvRSA/HF	-0.069	**3.2E-15**		n.a.		n.a.		7.5E-02	1.7E-01	23.8%	2.8E-01
4	12	rs7980799	RMSSD	-0.039	**3.2E-20**	**1.2E-12**	-0.034 (0.005)	**5.5E-11**	-0.032 (0.007)	**1.7E-06**	**6.1E-08**	**1.6E-04**	43.0%	**5.9E-06**
		rs1351682	pvRSA/HF	-0.073	**5.7E-15**	**6.7E-09**	n.a.		n.a.		**8.4E-05**	**1.1E-04**	1.5%	1.0E-01
		rs1384598	SDNN	-0.023	**7.4E-13**	**5.5E-08**	-0.019 (0.004)	**8.8E-07**	-0.012 (0.006)	3.2E-02	**2.9E-03**	6.6E-02	45.7%	**9.8E-05**

5	7	rs4262	SDNN	-0.028	**4.3E-17**	**3.1E-05**	-0.025 (0.004)	**1.1E-09**	-0.025 (0.006)	**4.7E-05**	**5.2E-06**	**9.8E-04**	36.6%	**4.4E-04**
			pvRSA/HF	-0.05	**1.8E-11**		n.a.		n.a.		**2.9E-05**	**3.9E-03**	31.0%	**9.2E-04**
		rs180238	RMSSD	-0.034	**8.0E-16**	**5.1E-06**	-0.035 (0.005)	**4.9E-11**	-0.029 (0.007)	**3.7E-05**	**1.9E-05**	**1.3E-03**	38.5%	**1.8E-03**
6	14b	rs4899412	SDNN	-0.026	**3.1E-13**	1.7E-02	-0.022 (0.004)	**3.1E-07**	-0.017 (0.006)	9.0E-03	**1.7E-03**	1.5E-02	31.9%	9.4E-03
		rs2052015	RMSSD	-0.036	**3.6E-10**	7.2E-02	-0.042 (0.004)	**3.0E-08**	-0.031 (0.009)	**6.6E-04**	**1.4E-04**	1.2E-02	46.1%	**1.7E-03**
	14c	rs2529471	SDNN	-0.021	**1.9E-12**	1.3E-02	-0.018 (0.004)	**1.6E-06**	-0.022 (0.006)	**1.0E-04**	**5.5E-06**	**2.5E-04**	29.1%	**1.2E-03**
	14a	rs36423	SDNN	-0.033	**6.3E-13**	**3.5E-03**	-0.028 (0.006)	**4.0E-07**	-0.030 (0.009)	**1.0E-03**	**4.9E-04**	**3.2E-03**	25.5%	2.2E-01
			RMSSD	-0.04	**5.4E-11**		-0.036 (0.008)	**2.4E-06**	-0.028 (0.010)	7.0E-03	7.5E-03	2.6E-02	32.0%	2.2E-01
7	15a	rs2680344	SDNN	-0.024	**4.9E-11**	**3.8E-09**	-0.019 (0.005)	**4.2E-05**	-0.026 (0.007)	**1.7E-04**	**6.2E-05**	**3.4E-04**	21.5%	1.2E-02
	15b	rs1812835	RMSSD	-0.025	**5.2E-10**	**1.1E-06**	-0.021 (0.005)	**3.1E-05**	-0.024 (0.007)	**2.3E-04**	**2.2E-03**	1.3E-02	33.8%	1.4E-02
8	20	rs6123471	RMSSD	-0.024	**1.3E-08**	**1.6E-20**	-0.022 (0.005)	**4.2E-05**	-0.015 (0.008)	4.5E-02	6.3E-03	6.8E-01	87.8%	**2.2E-05**

Chr: chromosome; HR: heart rate; CV: coefficient of variation; SE: standard error.
#Lifelines (N=12,101), NESDA (N=2,118), TRAILS-Pop (N=1,191).
*Sobel *p*-values of the meta-analyses are calculated from meta z-scores that are determined using a sqrt(sample size) weighted *z*-score meta-analysis.

Supplementary Table 9: Association between HRV SNPs and heart rate.

Results in (a) are the look-up results of the 17 HRV SNPs identified in this study in the meta-analysis of heart rate (Den Hoed et al., 2013). Panels (b) and (c) present the explained variances in heart rate in the Lifelines (n=12,101), NESDA (n=2,118), TRAILS-Pop (n=1,191), and ABCD (n=1,094) cohorts by (b) the weighted multi-SNP genetic risk score based on the independent genome-wide significant HRV SNPs in the stage 1+2 meta-analysis, and (c) the optimal polygenic risk scores computed at the *p*-value threshold that explained the largest percentage of phenotypic variance. ΔR^2 is the difference in percentage of explained variance by the multi-SNP genetic or polygenic risk score between the models with and without the risk score while adjusting both for age, sex, and principal components. The maximum number of overlap between the HRV and heart rate meta-analysis was N=10,612.

a) Look-up results

**Identified heart rate variability SNPs**					**Heart rate lookup**			
**Locus**	**Chr**	**HRV SNP**	**Position (bp)**	**HRV Trait**	**β#**	**SE**	**N**	***p*-value***
1	19	rs12974991	5845584	RMSSD	0.1521	0.1072	80077.3	1.77E-01
		rs12974440	5845386	pvRSA/HF	0.1505	0.1072	80091.2	1.77E-01
		rs12980262	5844058	SDNN	0.1486	0.1072	80091.3	1.77E-01
2	12	rs10842383	24663234	SDNN, RMSSD, pvRSA/HF	0.4922	0.0731	87263.7	**1.52E-10**
3	6	rs236349	36928543	SDNN, RMSSD, pvRSA/HF	0.2583	0.0598	67947	**3.95E-05**
4	12	rs7980799	33468257	RMSSD	0.4052	0.0542	85488.7	**1.19E-12**
		rs1351682	33490042	pvRSA/HF	0.3136	0.0514	93068.9	**6.72E-09**
		rs1384598	33514166	SDNN	0.305	0.0534	87057.3	**5.52E-08**
5	7	rs4262	93389364	SDNN, pvRSA/HF	0.2514	0.0574	82597.3	**3.13E-05**
		rs180238	93388383	RMSSD	0.2788	0.0581	86814.9	**5.08E-06**
6	14b	rs4899412	71534015	SDNN	0.1485	0.0594	89581.2	1.73E-02
		rs2052015	71556806	RMSSD	0.1473	0.0778	86557.5	7.19E-02
	14c	rs2529471	71883022	SDNN	0.1325	0.0505	93152.6	1.26E-02
	14a	rs36423	71422955	SDNN, RMSSD	0.2357	0.0767	90388.5	**3.49E-03**
7	15a	rs2680344	71440538	SDNN	0.3787	0.0611	91249.2	**3.80E-09**
	15b	rs1812835	71294557	RMSSD	0.2745	0.0536	84616.4	**1.09E-06**
8	20	rs6123471	36273570	RMSSD	0.5012	0.0513	89348.2	**1.56E-20**

Chr: chromosome; bp: base pair position based on build 36 (hg18); SE: standard error of β; N: sample size.

# Effect size is shown for the allele increasing the risk of low levels of HRV.

* *P*-values from the discovery stage using only Europeans. Significant *p*-values $<0.05/11$ (independent SNPs) are shown in bold.

b) Multi-SNP genetic risk scores

		Multi-SNP genetic risk score*											
		Lifelines			NESDA			TRAILS-Pop			ABCD		
trait	risk score	N SNPs	*p*-value	ΔR^2	N SNPs	*p*-value	ΔR^2	N SNPs	*p*-value	ΔR^2	N SNPs	*p*-value	ΔR^2
Heart rate	SDNN	9	3.70E-08	0.23%	9	1.40E-03	0.39%	10	1.40E-02	0.55%	10	2.70E-01	0.11%
Heart rate	RMSSD	7	1.90E-05	0.14%	11	1.60E-03	0.38%	11	3.50E-04	1.07%	11	1.90E-01	0.16%
Heart rate	pvRSA/HF	5	5.20E-04	0.09%	5	4.20E-02	0.13%	5	2.90E-04	1.13%	4	3.10E-01	0.10%

n.a.=not available.
* For Lifelines, NESDA, and TRAILS-Pop the weights (i.e. effects sizes) and number of genome-wide significant SNPs included in the risk score were adjusted by analytically extracting the cohort's effect size and standard error from the meta effect size and standard error, respectively, and recalculating the *p*-value based on these adjusted effect sizes and standard errors, since these cohorts were included in stage 1 and/or 2.

c) Polygenic risk scores

	Polygenic risk score*															
	Lifelines				NESDA				TRAILS-Pop				ABCD			
risk score	*p* cutoff	N SNPs	*p*-value	ΔR^2	*p* cutoff	N SNPs	*p*-value	ΔR^2	*p* cutoff	N SNPs	*p*-value	ΔR^2	*p* cutoff	N SNPs	*p*-value	ΔR^2
SDNN	<5E-3	3186	2.30E-08	0.25%	<5E-8	6	9.00E-03	0.29%	<5E-8	7	5.60E-03	0.69%	<5E-5	71	1.80E-02	0.51%
RMSSD	<5E-5	80	1.30E-10	0.34%	<5E-3	3341	1.70E-03	0.43%	<5E-7	11	5.80E-04	0.99%	<5E-4	466	5.80E-02	0.33%
pvRSA/HF	<0.05	20747	5.90E-06	0.17%	<5E-4	399	8.10E-04	0.50%	<5E-7	6	5.80E-03	0.68%	<5E-5	67	6.50E-02	0.31%

* Weighted polygenic risk score were determined based on independent SNPs in the stage 1 meta-analysis. For NESDA and TRAILS-Pop the weights (i.e. effects sizes) and *p*-values were adjusted by analytically extracting the cohort's effect size and standard error from the meta effect size and standard error, respectively, and recalculating the *p*-value based on these adjusted effect size and standard error, since these cohorts were included in stage 1.

Supplementary Table 10: Association between heart rate SNPs and the three HRV traits. Look-up of the 21 heart rate SNPs identified in the GWAS meta-analysis of heart rate by Den Hoed et al.[14] in the current study's meta-analyses results for the three HRV traits (a). The effects of the genetic risk score for the heart rate SNPs on SDNN, RMSSD, and pvRSA/HF (b). Explained variance in HRV traits in the Lifelines (n=12,101), NESDA (n=2,118), TRAILS-Pop (n=1,191), and ABCD (n=1,094) cohorts by the weighted multi-SNP genetic risk score based on the 21 heart rate SNPs (c), and the optimal polygenic risk scores for heart rate computed at the *p*-value threshold that explained the largest percentage of phenotypic variance in the HRV traits (d).

**(a) Look-up of previously identified heart rate loci**				**HRV look-up**				
**Locus**	**Chr**	**HR SNP**	**Position (bp)**	**HRV Trait**	**β#**	**SE**	**N**	**p-value***
1	14	rs365990	22931651	SDNN	-0.0132	0.0039	27257	**8.08E-04**
				RMSSD	-0.0153	0.0053	26367	4.28E-03
				pvRSA/HF†	-0.0189	0.0109	20660	2.18E-01
2	6	rs1015451	122173184	pvRSA/HF†	0.0353	0.0180	18020	8.27E-02
				SDNN	-0.0041	0.0059	24617	4.92E-01
				RMSSD	-0.0116	0.0086	23727	1.83E-01
3	7	rs13245899	100335067	SDNN	-0.0116	0.0046	27256	1.23E-02
				pvRSA/HF†	0.0158	0.0131	20659	8.31E-02
				RMSSD	0.0011	0.0063	26366	8.58E-01
4	1	rs11118555	206007476	SDNN	-0.0130	0.0057	25873	2.46E-02
				RMSSD	-0.0091	0.0081	24984	2.62E-01
				pvRSA/HF†	-0.0099	0.0167	19277	9.06E-01
5	11	rs174549	61327958	pvRSA/HF†	-0.0204	0.0114	20660	8.18E-02
				RMSSD	-0.0052	0.0055	26367	3.49E-01
				SDNN	-0.0024	0.0040	27257	5.54E-01
6	6	rs11153730	118774215	pvRSA/HF†	0.0226	0.0108	19277	4.10E-03
				SDNN	0.0019	0.0039	25041	6.29E-01
				RMSSD	0.0015	0.0052	24984	7.78E-01
7	12	rs17287293	24662145	pvRSA/HF†	-0.1263	0.0152	19081	**3.59E-21**
				SDNN	-0.0488	0.0052	25873	**5.46E-21**
				RMSSD	-0.0670	0.0071	24984	**1.25E-20**
8	20	rs6127471	36277452	RMSSD	-0.0283	0.0052	26367	**5.83E-08**
				SDNN	-0.0160	0.0038	27257	**2.58E-05**
				pvRSA/HF†	-0.0299	0.0107	20660	**6.84E-04**
9	2	rs17362588	179429291	RMSSD	-0.0376	0.0089	24981	**2.75E-05**
				pvRSA/HF†	-0.0560	0.0184	19274	**7.16E-05**
				SDNN	-0.0233	0.0062	25871	**1.91E-04**
10	12	rs7980799	33468257	RMSSD	-0.0402	0.0052	26156	**1.92E-14**
				pvRSA/HF†	-0.0719	0.0111	20464	**1.29E-11**
				SDNN	-0.0232	0.0038	26992	**1.80E-09**
11	15	rs4489968	71452559	SDNN	-0.0242	0.0050	27256	**1.94E-06**
				RMSSD	-0.0309	0.0071	26366	**1.47E-05**
				pvRSA/HF†	-0.0469	0.0144	20659	**3.97E-04**
12	3	rs7612445	180655673	SDNN	-0.0233	0.0048	27251	**1.60E-06**
				RMSSD	-0.0295	0.0067	26366	**1.13E-05**
				pvRSA/HF†	-0.0333	0.0136	20659	5.68E-03

13	14	rs17796783	84879664	RMSSD	-0.0121	0.0055	26153	3.00E-02
				SDNN	-0.0046	0.0041	26989	2.63E-01
				pvRSA/HF†	0.0042	0.0113	20657	6.39E-01
14	7	rs2350782	136293174	pvRSA/HF†	-0.0387	0.0189	17983	5.57E-03
				RMSSD	-0.0156	0.0095	23691	1.04E-01
				SDNN	-0.0100	0.0066	24514	1.28E-01
15	5	rs6882776	172596769	pvRSA/HF†	0.0209	0.0134	18716	3.14E-02
				RMSSD	0.0054	0.0065	24212	4.10E-01
				SDNN	0.0031	0.0047	25048	5.09E-01
16	7	rs180242	93387532	RMSSD	-0.0417	0.0061	22345	**1.44E-11**
				SDNN	-0.0284	0.0044	23235	**1.27E-10**
				pvRSA/HF†	-0.0499	0.0127	16638	**1.56E-07**
17	2	rs13030174	231979528	RMSSD	-0.0034	0.0057	26363	5.54E-01
				pvRSA/HF†	-0.0028	0.0118	20656	8.29E-01
				SDNN	0.0004	0.0042	27253	9.28E-01
18	3	rs9647379	173267862	SDNN	-0.0073	0.0041	25874	7.54E-02
				RMSSD	-0.0094	0.0057	24984	9.92E-02
				pvRSA/HF†	-0.0071	0.0116	19277	1.69E-01
19	12	rs2067615	105673552	SDNN	0.0024	0.0038	25874	5.31E-01
				RMSSD	-0.0010	0.0051	24984	8.45E-01
				pvRSA/HF†	-0.0018	0.0110	19081	8.88E-01
20	12	rs826838	37392998	pvRSA/HF†	-0.0470	0.0108	20464	**2.84E-09**
				RMSSD	-0.0290	0.0050	26367	**1.04E-08**
				SDNN	-0.0172	0.0037	27257	**3.36E-06**
21	2	rs4140885	188041309	pvRSA/HF†	0.0109	0.0115	20660	1.56E-01
				SDNN	-0.0005	0.0040	27257	9.00E-01
				RMSSD	-0.0001	0.0056	26367	9.82E-01

Chr: chromosome; bp: base pair position based on build 36 (hg18); HR: heart rate; SE: standard error of β; N: sample size.
Effect size is shown for the allele increasing the heart rate.
**P*-values from the discovery stage using only Europeans. Significant *p*-values <0.05/21 are shown in bold.

(b) effect of the multi-SNP genetic risk score of heart rate composed of all genome-wide significant SNPs based on summary statistics

			genetic risk score heart rate	
**HRV parameter**	**Sample size**	**N SNPs**	**Effect size (95% CI)**	***p*-value***
SDNN	27257	20	-0.032 (-0.04,-0.03)	**1.7E-37**
RMSSD	26367	21	-0.043 (-0.05,-0.04)	**1.0E-38**
pvRSA/HF	20660	21	-0.05 (-0.06,-0.04)	**1.6E-13**

†*p*-value, allele, EAF, N from *p*-value weighted meta-analysis of all cohorts using METAL and β, SE from inverse-variance meta-analysis of only HF cohorts using GWAMA.

(c) effects of the multi-SNP genetic risk score of heart rate based on individual level data

	Multi-SNP genetic risk score*											
	Lifelines			NESDA			TRAILS-Pop			ABCD		
**Trait**	**N SNPs**	***p*-value**	**ΔR^2**	**N SNPs**	***p*-value**	**ΔR^2**	**N SNPs**	***p*-value**	**ΔR^2**	**N SNPs**	***p*-value**	**ΔR^2**
SDNN	15	3.30E-09	0.24%	21	4.60E-07	0.90%	21	6.00E-02	0.29%	20	4.90E-02	0.35%
RMSSD	15	3.20E-15	0.44%	21	2.70E-07	0.90%	21	1.50E-02	0.49%	20	1.70E-02	0.52%
pvRSA/HF	15	n.a.	n.a.	21	2.10E-06	0.64%	21	5.40E-02	0.31%	20	6.70E-02	0.31%
heart rate	15	3.30E-41	1.44%	21	2.50E-10	1.69%	21	7.30E-05	1.25%	20	4.60E-03	0.73%

* For Lifelines, NESDA, and TRAILS-Pop the weights (i.e. effects sizes) and number of genome-wide significant SNPs included in the risk score were adjusted by analytically extracting the cohort's effect size and standard error from the meta effect size and standard error, respectively, and recalculating the *p*-value based on these adjusted effect sizes and standard errors, since these cohorts were included in stage 1 and/or 2.

(d) effects of the polygenic risk score of heart rate based on individuals level data

	Polygenic risk score*															
	Lifelines				NESDA				TRAILS-Pop				ABCD			
**Trait**	***p* cut-off**	**N SNPs**	***p*-value**	**ΔR^2**	***p* cut-off**	**N SNPs**	***p*-value**	**ΔR^2**	***p* cut-off**	**N SNPs**	***p*-value**	**ΔR^2**	***p* cut-off**	**N SNPs**	***p*-value**	**ΔR^2**
SDNN	<5E-8	19	1.20E-09	0.26%	<5E-4	430	5.00E-06	0.78%	<5E-5	97	1.80E-02	0.46%	<5E-4	438	1.30E-02	0.57%
RMSSD	<5E-8	19	3.50E-16	0.48%	<5E-4	430	1.90E-06	0.82%	<5E-8	18	8.20E-03	0.58%	<5E-4	438	3.80E-02	0.39%
pvRSA/ HF	n.a.	n.a.	n.a.	n.a.	<5E-4	430	1.50E-06	0.70%	<5E-5	97	2.70E-02	0.41%	<5E-2	18305	1.50E-01	0.19%
heart rate	<5E-7	28	3.50E-42	1.50%	<5E-8	21	8.10E-10	1.66%	<5E-8	18	3.10E-06	1.73%	<5E-4	438	1.40E-04	1.32%

* Weighted polygenic risk score were determined based on independent SNPs in the stage 1 meta-analysis. For NESDA and TRAILS-Pop the weights (i.e. effects sizes) and *p*-values were adjusted by analytically extracting the cohort's effect size and standard error from the meta effect size and standard error, respectively, and recalculating the *p*-value based on these adjusted effect size and standard error, since these cohorts were included in stage 1.

Supplementary Table 11: Look-up results of genome-wide association consortia targeting cardiometabolic outcomes.
In panel (a) results are shown for the associations with the genetic risk scores composed of the HRV SNPs identified in this study. Panel (b) shows the genetic correlations with the HRV traits are shown as computed using LD Score regression based on GWAS summary statistics.

(a)			SDNN		RMSSD		pvRSA/HF*	
Trait or disease	Reference	Sample size	Effect size# (95% CI)	*p*-value	Effect size# (95% CI)	*p*-value	Effect size# (95% CI)	*p*-value
Heart rate	Den Hoed et al., 2013[14]	93153	8.65 (7.35,9.94)	**3.7E-39**	6.93 (6.02,7.83)	**1.5E-50**	2.66 (2.10,3.23)	**2.2E-20**
Systolic blood pressure	Ehret et al., 2011[15]	69616	1.64 (-0.79,4.08)	1.9E-01	0.94 (-0.78,2.65)	2.8E-01	0.16 (-0.90,1.22)	7.7E-01
Diastolic blood pressure	Ehret et al., 2011[15]	69604	-0.37 (-1.91,1.17)	6.4E-01	-0.83 (-1.91,0.25)	1.3E-01	-0.22 (-0.89,0.45)	5.2E-01
Heart failure	Smith et al., 2010[16]	20926	0.58 (0.27,1.23)	1.6E-01	0.69 (0.41,1.16)	1.6E-01	0.90 (0.66,1.25)	5.2E-01
Coronary Artery Disease	Deloukas et al., 2013[17]	83174	1.04 (0.72,1.52)	8.3E-01	1.08 (0.83,1.37)	6.0E-01	0.96 (0.82,1.12)	6.4E-01
Atrial fibrillation	Ellinor et al., 2012[18]	59133	0.62 (0.38,1.02)	5.8E-02	0.72 (0.51,1.02)	6.7E-02	1.15 (0.93,1.43)	2.1E-01
Sudden cardiac death	SCD consortium, unpublished	29928	1.10 (0.52,2.33)	8.0E-01	1.37 (0.81,4.55)	2.4E-01	0.95 (0.68,1.32)	7.6E-01
Type 2 diabetes	Morris et al., 2012[19]	69033	0.99 (0.86,1.14)	9.1E-01	1.01 (0.90,1.11)	9.2E-01	0.96 (0.83,1.11)	6.1E-01
Body mass index	Locke et al., 2015[20]	233947	-0.0052 (-0.10,0.09)	9.1E-01	0.01 (-0.05,0.07)	7.3E-01	-0.0074 (-0.035,0.03)	7.2E-01
eGFR for creatinin	Pattaro et al., 2016[21]	74354	0.02 (-0.01,0.05)	1.7E-01	0.005 (-0.02,0.03)	6.3E-01	0.001 (-0.01,0.01)	9.4E-01
Urinary albumin excretion(UACR)	Teumer et al., 2016[22]	31077	0.11 (-0.13,0.34)	4.2E-01	0.10 (-0.06,0.27)	2.2E-01	0.08 (-0.03,0.18)	1.4E-01

* *p*-value from *z*-score weighted meta-analysis of all cohorts using METAL and β and standard error from inverse-variance meta-analysis of only HF cohorts using GWAMA were used
# Effect size is either the incremental change in the phenotype for quantitative traits or the odds ratio for binary traits when the multi-SNP genetic risk score of HRV decreases by one unit.

(b)<br>Trait or disease	Reference	Sample size	SDNN<br>genetic correlation (SE)	SDNN<br>*p*-value	RMSSD<br>genetic correlation (SE)	RMSSD<br>*p*-value	pvRSA/HF*<br>genetic correlation (SE)	pvRSA/HF*<br>*p*-value
Heart rate	Den Hoed et al., 2013[14]	93153	-0.656 (0.09)	**1.0E-12**	-0.738 (0.10)	**1.3E-14**	-0.548 (0.11)	**2.2E-07**
Systolic blood pressure	Ehret et al., 2011[15]	69616	-0.204 (0.08)	8.4E-03	-0.230 (0.08)	6.4E-03	-0.314 (0.10)	**2.1E-03**
Diastolic blood pressure	Ehret et al., 2011[15]	69604	-0.267 (0.08)	**4.0E-04**	-0.271 (0.08)	**4.0E-04**	-0.349 (0.10)	**3.0E-04**
Heart failure	Smith et al., 2010[16]	20926	-0.291 (0.20)	1.5E-01	-0.095 (0.19)	6.1E-01	-0.281 (0.20)	1.5E-01
Coronary Artery Disease	Deloukas et al., 2013[17]	83174	-0.133 (0.09)	1.2E-01	-0.113 (0.09)	2.3E-01	-0.116 (0.10)	2.3E-01
Atrial fibrillation	Ellinor et al., 2012[18]	59133	0.050 (0.13)	7.0E-01	0.058 (0.13)	1.3E-01	-0.019 (0.12)	8.8E-01
Sudden cardiac death	SCD consortium, unpublished	29928	-0.123(0.23)	5.9E-01	0.080(0.22)	7.2E-01	-0.100 (0.26)	7.0E-01
Type 2 diabetes	Morris et al., 2012[19]	69033	-0.263 (0.11)	1.2E-02	-0.209 (0.13)	1.2E-01	-0.160 (0.13)	2.1E-01
Body mass index	Locke et al., 2015[20]	233947	-0.085 (0.04)	4.5E-02	0.042 (0.05)	3.6E-01	-0.036 (0.05)	4.5E-01
eGFR for creatinin	Pattaro et al., 2016[21]	175579	-0.011 (0.07)	8.7E-01	-0.017 (0.07)	8.1E-01	-0.031 (0.07)	6.6E-01
Urinary albumin excretion(UACR)	Teumer et al., 2016[22]	51886	0.002 (0.13)	9.9E-01	-0.055 (0.14)	6.9E-01	-0.183 (0.15)	2.1E-01

* *p*-value from *z*-score weighted meta-analysis of all cohorts using METAL and β and standard error from inverse-variance meta-analysis of only HF cohorts using GWAMA were used.

Supplementary Table 12: Effects of the HRV SNPs identified in this study on atrial fibrillation (N=59,133).

Odds ratios (OR) with confidence intervals (95%CI) are given for the HRV decreasing allele. Significantly associated SNPs (*p*-value <0.0045) are shown in bold.

Locus	Chr	SNP	Position (bp)	HRV trait	OR (95%CI)	*p*-value
1	19	rs12974440	5845584	RMSSD	1.00 (0.92-1.07)	9.2E-01
		rs12974991	5845386	pvRSA/HF	1.00 (0.92-1.07)	9.2E-01
		rs12980262	5844058	SDNN	1.00 (0.92-1.07)	9.3E-01
2	12	rs10842383	24663234	SDNN, RMSSD, pvRSA/HF	**1.16 (1.10-1.23)**	**3.5E-07**
3	6	rs236349	36928543	SDNN, RMSSD, pvRSA/HF	0.94 (0.91-0.98)	5.3E-03
4	12	rs1351682	33468257	pvRSA/HF	0.98 (0.95-1.03)	4.5E-01
		rs1384598	33490042	SDNN	0.99 (0.95-1.03)	6.4E-01
		rs7980799	33514166	RMSSD	0.99 (0.95-1.03)	6.5E-01
5	7	rs180238	93389364	RMSSD	1.00 (0.96-1.05)	8.7E-01
		rs4262	93388383	SDNN, pvRSA/HF	1.02 (0.98-1.06)	3.8E-01
6	14b	rs2052015	71534015	RMSSD	1.00 (0.94-1.07)	9.7E-01
		rs2529471	71556806	SDNN, RMSSD	0.96 (0.92-0.99)	2.3E-02
	14c	rs36423	71883022	SDNN, RMSSD	0.94 (0.88-1.00)	4.2E-02
	14a	rs4899412	71422955	SDNN	0.98 (0.94-1.03)	5.0E-01
7	15a	rs1812835	71440538	SDNN, RMSSD	0.95 (0.92-0.99)	2.2E-02
	15b	rs2680344	71294557	SDNN, RMSSD	**0.89 (0.84-0.93)**	**4.3E-07**
8	20	rs6123471	36273570	RMSSD	0.99 (0.95-1.02)	4.5E-01

Chr: chromosome; bp: base pair position (build 36).

Supplementary Table 13: RegulomeDB main results of functional variant analysis.

HRV SNP	Chr	Position (bp; b37)	RegulomeDB score
rs12980262	19	5893057	TF binding + any motif + DNase Footprint + DNase peak
rs12974440	19	5894385	TF binding + any motif + DNase peak
rs12974991	19	5894583	TF binding + DNase peak
rs236349	6	36820564	TF binding + DNase peak
rs180238	7	93550446	TF binding + DNase peak
rs4262	7	93551427	TF binding + DNase peak
rs36423	14	72353201	TF binding or DNase peak
rs2529471	14	72813268	TF binding or DNase peak
rs2680344	15	73653484	TF binding or DNase peak
rs6123471	20	36840155	TF binding or DNase peak
rs7980799	12	33576989	other
rs1351682	12	33598774	other
rs1384598	12	33622898	other
rs2052015	14	72487052	other
rs1812835	15	73507503	other
rs10842383	12	24771966	NA
rs4899412	14	72464261	NA

TF binding: the variant is located at a site where transcription factors are anticipated to bind.

Supplementary Table 14: eQTL analysis. (a) The 17 HRV SNPs were assessed in the NESDA-NTR eQTL database[23, 24]. Only SNPs with a significant *cis* effect on gene expression are shown. All HRV SNPs with significant results in the NESDA/NTR eQTL database[23, 24] were looked up in several other eQTL databases for multiple tissues: (b) a second [25] and (c) third independent whole blood eQTL database [26], (d) lymphoblastoid cell lines[27], (e) eQTLs for ten different regions of the brain [28], (f) GTex, and (g) a heart eQTL database [29].

**(a)**			**Association between HRV SNP and gene expression in NESDA/NTR**			**Top eQTL with strongest association with gene expression**			***p*-value HRV SNP conditional on top eQTL**
**HRV SNP**	**Gene**	**chr:bp_allele ; Probeset_id**	***p*-value**	**FDR**	**Beta**	**SNP & Allele**	***p*-value**	**LD HRV SNP**	
rs4262	*GNG11*	7:93551428_C ; 11722379_at	2.35E-07	1.75E-03	-0.100	7:93540958_C	1.48E-08	0.797	>0.05
rs180238	*GNG11*	7:93550447_C ; 11722379_at	6.00E-08	5.26E-04	-0.109	7:93540958_C	1.48E-08	0.971	>0.05
rs4899412	*RGS6*	14:72464262_T ; 11752774_a_at	2.71E-06	1.60E-02	0.097	14:72465028_C	2.35E-06	0.999	>0.05
rs4899412	*RGS6*	14:72464262_T ; 11750930_a_at	4.63E-07	3.50E-03	0.108	14:72425522_T	1.86E-07	0.896	>0.05
rs1812835	*NEO1*	15:73507504_A ; 11717210_a_at	1.65E-07	1.37E-03	-0.106	15:73420724_A	3.12E-09	0.775	>0.05
rs1812835	*NEO1*	15:73507504_A ; 11717212_a_at	5.91E-11	<1.34e-05	-0.131	15:73377436:G_GT_I	2.89E-13	0.743	>0.05

**(b)**			**Association between HRV SNP and expression in whole blood**		**Top eQTL in BIOS whole blood**		
**HRV SNP**	**Gene**	**chr:bp_allele**	***p*-value**	***Z*-score**	**SNP**	***p*-value**	**LD HRV SNP**
rs4262	*GNG11*	7:93551428_C	NS	NS	rs180279	1.45E-19	0.002
rs180238	*GNG11*	7:93550447_C	NS	NS	rs180279	1.45E-19	0.001
rs4899412 *(by its proxy rs1987722 [r^2=0.99])*	*RGS6*	14:72465028_C	1.27E-36	12.64	rs1987722	1.27E-36	0.99
rs1812835	*NEO1*	15:73507504_A	4.93E-80	-18.944	rs1038137	1.72E-119	0.75

**(c)**			**Association between HRV SNP and expression in whole blood**		**Top eQTL in Westra et al. whole blood**		
**HRV SNP**	**Gene**	**chr:bp_allele**	***p*-value**	***Z*-score**	**SNP**	***p*-value**	**LD HRV SNP**
rs4262 *(by its proxy rs180236 r^2=0.77)*	*GNG11*	7:93391277_A	1.12E-04	3.86	rs180275	7.16E-09	0.002
rs180238 *(by its proxy rs180236 r^2=0.32)*	*GNG11*	7:93391277_A	1.12E-04	3.86	rs180275	7.16E-09	0.001
rs4899412	*RGS6*	n.a.	n.a.	n.a.	rs8008967	6.83E-04	0.002
rs1812835	*NEO1*	15:73507504_A	5.60E-04	-3.45	rs3784801	1.63E-04	0.92

**(d)**			**Association between HRV SNP and expression in Geuvadis**		**Top eQTL in Geuvadis**		
**HRV SNP**	**Gene**	**chr:bp_allele**	***p*-value**	**Beta**	**SNP**	***p*-value**	**LD HRV SNP**
rs4262	*GNG11*	7:93551428_C	NS	NS	NS	NS	NS
rs180238	*GNG11*	7:93550447_C	NS	NS	NS	NS	NS
rs4899412	*RGS6*	14:72464262_T	NS	NS	rs1548687	3.44E-07	0.002
rs1812835	*NEO1*	*15:73507504_A*	1.39E-17	n.a.	rs62016808	4.18E-25	0.75

**(e)**			**Association between HRV SNP and expression in Braineac (tissue)**		**Top eQTL in Braineac**		
**HRV SNP**	**Gene**	**chr:bp_allele**	***p*-value**	**Beta**	**SNP**	***p*-value**	**LD HRV SNP**
rs4262	*GNG11*	7:93551428_C	2.8E-04 (medulla)	n.a.	rs4266	1.20E-05	1
rs180238	*GNG11*	7:93550447_C	0.0013 (medulla)	n.a.	rs4266	1.20E-05	0.047
rs4899412	*RGS6*	14:72464262_T	NS	n.a.	NS		
rs1812835	*NEO1*	15:73507504_A	NS	n.a.	NS		

**(f)**			**Association between HRV SNP and expression in GTEx (tissue)**		**Top eQTL in GTEx**		
**HRV SNP**	**Gene**	**chr:bp_allele**	***p*-value**	**Beta**	**SNP**	***p*-value**	**LD HRV SNP**
rs4262	*GNG11*	7:93551428_C	8.10E-09 (Tibial artery)	-0.21	rs4262	8.10E-09	1
rs180238	*GNG11*	7:93550447_C	NS	NS	rs4262	8.10E-09	0.81
rs4899412	*RGS6*	14:72464262_T	NS	NS	rs2108469	4.10E-10	0.006
rs1812835	*NEO1*	15:73507504_A	4.4E-06 (EBV- lympho-blastoid cell line)	-0.62	rs2169951	NS	0.84

**(g)**			**Association between HRV SNP and expression in Koopmans Heart eQTL database**		**Top eQTL in Heart eQTL database**		
**HRV SNP**	**Gene**	**chr:bp_allele**	***p*-value**	**Beta**	**SNP**	***p*-value**	**LD HRV SNP**
rs4262	*GNG11*	7:93551428_C	NS	NS		NS	
rs180238	*GNG11*	7:93550447_C	NS	NS		NS	
rs4899412	*RGS6*	14:72464262_T	NS	NS		NS	
rs1812835	*NEO1*	15:73507504_A	NS	NS		NS	

chr: chromosome; bp: base pair position (build 37); FDR: false discovery rate; LD: linkage disequilibrium (r^2**).**

Supplementary Table 15: mQTL analysis using the BIOS (*cis*) mQTL database[30] to assess the effect of the 17 HRV SNPs on methylation in nearby genes.

HRV SNP	Chr:bp	Effect allele	mQTL							Top mQTL		
			Methylation Site	Gene(s)	Position methylation site (bp)	Location in gene region	Relation to UCSC CpG Island	*p*-value	Beta	SNP	*p*-value	LD HRV SNP
rs12974440	19:5894386	A	cg22854549	*VMAC; NDUFA11*	5904785	TSS200; TSS1500	Island	5.72E-82	-19.18	rs55660714	1.71E-103	0.73
rs12974440	19:5894386	A	cg03715305	*NDUFA11*	5894715	3'UTR	S_Shelf	1.63E-25	-10.44	rs17271904	3.89E-26	0.98
rs12974440	19:5894386	A	cg19211619	*CAPS*	5913923	TSS1500	N_Shore	8.07E-07	4.93	rs12982903	1.76E-07	0.95
rs12974991	19:5894584	A	cg22854549	*VMAC; NDUFA11*	5904785	TSS200; TSS1500	Island	8.38E-83	-19.28	rs55660714	1.71E-103	0.71
rs12974991	19:5894584	A	cg03715305	*NDUFA11*	5894715	3'UTR	S_Shelf	4.21E-26	-10.57	rs17271904	3.89E-26	1.00
rs12974991	19:5894584	A	cg19211619	*CAPS*	5913923	TSS1500	N_Shore	3.47E-07	5.10	rs12982903	1.76E-07	0.97
rs12980262	19:5893058	A	cg22854549	*VMAC; NDUFA11*	5904785	TSS200; TSS1500	Island	9.98E-82	-19.15	rs55660714	1.71E-103	0.73
rs12980262	19:5893058	A	cg03715305	*NDUFA11*	5894715	3'UTR	S_Shelf	1.52E-25	-10.45	rs17271904	3.89E-26	0.98
rs12980262	19:5893058	A	cg19211619	*CAPS*	5913923	TSS1500	N_Shore	7.94E-07	4.94	rs12982903	1.76E-07	0.95
rs7980799	12:33576990	A	cg21043657	*SYT10*	33590837	Body	N_Shore	1.72E-23	9.99	rs6488162	2.92E-25	0.90
rs1351682	12:33598775	G	cg21043657	*SYT10*	33590837	Body	N_Shore	5.52E-21	9.40	rs6488162	2.92E-25	0.80
rs1384598	12:33622899	T	cg21043657	*SYT10*	33590837	Body	N_Shore	9.71E-21	9.34	rs6488162	2.92E-25	0.80
rs4262	7:93551428	C	cg08038054	*GNG11*	93550781	TSS1500		5.22E-51	-15.02	rs180236	2.60E-55	0.87
rs4262	7:93551428	C	cg06439941	*GNG11*	93550756	TSS1500		3.63E-30	-11.41	rs180236	9.11E-36	0.87
rs2529471	14:72813269	C	cg17922283	*RGS6*	72799938	Body		2.84E-14	7.61	rs2090737	3.74E-19	0.71
rs4899412	14:72464262	T	cg19493789	*RGS6*	72396233		N_Shelf	NS	NS	rs2238280	4.16E-07	0.88
rs1812835	15:73507504	A	cg11357013	*NEO1*	73588054	Body		6.18E-30	-11.37	rs62016851	1.15E-31	0.93
rs1812835	15:73507504	A	cg19281068	*NEO1*	73345607	Body	S_Shore	3.88E-17	8.42	rs4609810	7.37E-22	0.78
rs1812835	15:73507504	A	cg11552023	*NEO1*	73595120	3'UTR		7.39E-10	-6.16	rs4132536	2.28E-17	0.77
rs1812835	15:73507504	A	cg17150474	*NEO1*	73343980	TSS1500	Island	3.29E-09	5.92	rs1023924	1.31E-11	0.83

Chr: chromosome; bp: base pair position (build 37); LD: linkage disequilibrium (r^2). TSS200= methylation site is located within 200 bp of a transcription start site; TSS1500= methylation site is located within 1500 bp of a transcription start site; 3’UTR= methylation site is located in the 3’untranslated region; Body= methylation site is located within a gene (gene body); Island= methylation site is located in a CpG island; S_Shelf= methylation site is located in the south shelf of a CpG island; N_Shelf= methylation site is located in the north shelf of a CpG island; S_Shore= methylation site is located in the south shore of a CpG island; N_Shore= methylation site is located in the north shore of a CpG island

Supplementary Table 16: DEPICT tissue enrichment analysis summarized by tissue or cell type.
Genes were considered to be enriched for certain tissues or cell-types if they showed a Z-score for enrichment > 2.0 in Supplementary Data 3.

	**Enriched tissue or cell type**												
**Gene**	**Blood**	**Central Nervous System**	**Respiratory**	**Salivary/ mucosa**	**Immuno logical**	**Stem cells**	**Heart**	**Muscle**	**Sensory**	**Epithelium**	**Endo crine**	**(Embryonic) membrane**	**Vasculature**
C6orf89	x				x	x		x					
CAPS			x	x					x	x			
CPNE5	x	x	x		x								
FUT5	x			x				x					
GNG11	x									x		x	x
GNGT1	x			x					x			x	
HCN4	x		x	x		x	x						
KIAA1755		x					x						
LINC00477	x		x		x			x					
NDUFA11							x		x		x		
NEO1		x											
PPIL1	x				x	x							
RANBP3		x									x		
RGS6	x	x				x							
**Sum**	9	5	4	4	4	4	3	3	3	2	2	2	1

Supplementary Table 17: Results of VEGAS showing gene-based significance based on the stage 1 GWAS meta-analyses results for SDNN, RMSSD, and pvRSA/HF. Genes are shown for which the i-value was significant at the multiple testing correction level of $2.5x10^{-6}$ (bolded) for at least one of the HRV traits. Genes in red italics were newly detected compared to the SNP-based analysis.

		SDNN				RMSSD				pvRSA/HF			
Chr	Gene	nSNPs	*p*-value	Best-SNP	SNP-*p*-value	nSNPs	*p*-value	Best-SNP	SNP-*p*-value	nSNPs	*p*-value	Best-SNP	SNP-*p*-value
2	*CCDC141*	149	2.01E-03	rs17362588	0.000191	149	**<1.0E-06**	rs13004438	1.58E-07	149	5.41E-04	rs12693173	1.05E-05
7	*TFPI2*	133	**<1.0E-06**	rs4262	1.56E-12	110	**<1.0E-06**	rs180238	2.24E-13	134	**<1.0E-06**	rs4262	7.41E-09
7	*GNGT1*	120	**<1.0E-06**	rs4262	1.56E-12	102	**<1.0E-06**	rs180238	2.24E-13	121	**<1.0E-06**	rs4262	7.41E-09
7	*GNG11*	120	**<1.0E-06**	rs4262	1.56E-12	106	**<1.0E-06**	rs180238	2.24E-13	121	**<1.0E-06**	rs4262	7.41E-09
12	*C12orf67*	98	**<1.0E-06**	rs10842383	1.51E-22	95	**<1.0E-06**	rs10842383	2.61E-22	98	**<1.0E-06**	rs10842383	7.61E-23
12	*SYT10*	181	**<1.0E-06**	rs1384598	1.20E-09	176	**<1.0E-06**	rs7980799	1.92E-14	176	**<1.0E-06**	rs6488162	2.42E-12
12	*ALG10*	80	1.74E-03	rs1705748	0.000109	78	3.00E-06	rs1705748	6.80E-09	79	**2.00E-06**	rs4001713	4.52E-09
12	*ALG10B*	101	4.45E-04	rs11183514	4.69E-05	90	**<1.0E-06**	rs4575342	1.34E-08	101	**<1.0E-06**	rs4575342	9.55E-10
12	*CPNE8*	289	1.27E-04	rs826879	2.74E-06	272	**<1.0E-06**	rs826879	8.41E-09	284	**<1.0E-06**	rs11168761	6.08E-10
14	*RGS6*	1243	**1.00E-06**	rs36423	1.03E-08	1216	4.00E-06	rs2052015	5.97E-09	1236	1.85E-02	rs17108294	0.0001619
15	*HCN4*	110	**<1.0E-06**	rs2680344	1.26E-07	109	**<1.0E-06**	rs7173389	1.16E-06	110	3.60E-05	rs2680344	4.23E-06
19	*NRTN*	56	6.00E-05	rs8108862	4.03E-12	54	**<1.0E-06**	rs8108862	2.77E-28	55	**<1.0E-06**	rs8108862	1.81E-37
19	*FUT6*	58	1.10E-05	rs8108862	4.03E-12	57	**<1.0E-06**	rs8108862	2.77E-28	57	**<1.0E-06**	rs8108862	1.81E-37
19	*FUT3*	74	**<1.0E-06**	rs12980262	3.00E-13	72	**<1.0E-06**	rs12974991	7.80E-30	73	**<1.0E-06**	rs12974440	9.09E-38
19	*FUT5*	75	**2.00E-06**	rs12980262	3.00E-13	73	**<1.0E-06**	rs12974991	7.80E-30	74	**<1.0E-06**	rs12974440	9.09E-38
19	*NDUFA11*	72	**1.00E-06**	rs12980262	3.00E-13	68	**<1.0E-06**	rs12974991	7.80E-30	70	**<1.0E-06**	rs12974440	9.09E-38
19	*VMAC*	71	3.00E-06	rs12980262	3.00E-13	66	**<1.0E-06**	rs12974991	7.80E-30	69	**<1.0E-06**	rs12974440	9.09E-38
19	*CAPS*	72	**1.00E-06**	rs12980262	3.00E-13	67	**<1.0E-06**	rs12974991	7.80E-30	70	**<1.0E-06**	rs12974440	9.09E-38
19	*RANBP3*	94	3.00E-06	rs12980262	3.00E-13	89	**<1.0E-06**	rs12974991	7.80E-30	93	**<1.0E-06**	rs12974440	9.09E-38
19	*RFX2*	101	2.82E-03	rs7258475	2.11E-12	98	5.00E-06	rs7258475	3.50E-26	102	**2.00E-06**	rs7258475	3.46E-30
20	*KIAA1755*	147	9.00E-06	rs6127466	1.53E-05	144	**<1.0E-06**	rs6123471	5.15E-08	143	2.85E-03	rs6123471	0.0003297

Supplementary Table 18: Common SNP and narrow-sense heritabilities of the HRV traits and heart rate as calculated (a) by genomic restricted maximum likelihood analysis in 9,571 unrelated individuals from the Lifelines Cohort Study, (b) by LD Score regression on the stage 1 meta-analysis results and (c) from classical biometrical modeling in the Oman Family Study.

For (a): Common SNP heritabilities are shown on the diagonal, genetic correlations above the diagonal. For (b): Narrow-sense heritabilities are shown on the diagonal, genetic correlations above the diagonal. For (c): Narrow-sense heritabilities are shown on the diagonal, genetic correlations above the diagonal, and environmental correlations below the diagonal. The *p*-values (between brackets) indicate whether the genetic correlation is different from 1.

**(a)**	SDNN	RMSSD	pvRSA/HF
SDNN	0.1076	0.98 (n.s.)	n.a.
RMSSD		0.1319	n.a.
pvRSA/HF			n.a.

n.a.: not applicable.

**(b)**	SDNN	RMSSD	pvRSA/HF
SDNN	0.1112	1.00 (n.s.)	1.00 (n.s.)
RMSSD		0.1125	1.00 (n.s.)
pvRSA/HF			0.1177

**(c)**	SDNN	RMSSD	HF
SDNN	0.1521	0.71 (2.5E-06)	0.73 (1.7E-05)
RMSSD	0.74	0.1989	0.90 (n.s.)
HF	0.74	0.80	0.1723
Heart Rate	-0.61	-0.69	-0.64

n.s.: not significant.

Supplementary Table 19: Prioritization of genes in HRV GWAS-identified loci (±40 kb of lead SNPs) using MetaRanker, ToppGene, Endeavour, and DEPICT.

HRV SNP	Chr	Gene	*p*-value for the gene being causal*						Prioritized for functional follow-up
			MetaRanker	ToppGene	Endeavour	DEPICT#			
						SDNN	RMSSD	pvRSA/HF	
rs10842383	12	*LINC00477*	6.2E-13	-	-	0.53	0.43	0.07	1
rs12974991, rs12974440, rs12980262	19	*AC024592.9*	-	0.06	-	-	-	-	0
rs12974991, rs12974440, rs12980262	19	*CAPS*	7.5E-13	-	-	0.30	0.64	0.58	1
rs12974991, rs12974440, rs12980262	19	*FUT5*	3.2E-13	0.07	0.56	0.78	0.35	0.52	1
rs12974991, rs12974440, rs12980262	19	*NDUFA11*	7.7E-14	0.06	-	0.52	0.54	0.42	1
rs12974991, rs12974440, rs12980262	19	*RANBP3*	-	-	-	0.86	0.76	0.71	1
rs12974991, rs12974440, rs12980262	19	*VMAC*	7.7E-13	-	-	-	-	-	0
rs236349	6	*C6orf89*	2.1E-13	0.14	0.78	0.97	0.81	0.45	1
rs236349	6	*CPNE5*	2.5E-13	-	-	0.42	0.14	0.17	1
rs236349	6	*PPIL1*	1.1E-13	-	-	0.12	0.26	0.78	1
rs2680344, rs1812835	15	*HCN4*	4.9E-15	-	-	0.45	0.09	0.09	1
rs2680344, rs1812835	15	*NEO1*	-	-	-	0.58	0.21	-	1
rs4262, rs180238	7	*GNG11*	4.5E-13	-	-	0.74	0.30	0.24	1
rs4262, rs180238	7	*GNGT1*	1.5E-13	2.3E-03	0.19	0.84	0.32	0.51	1
rs4262, rs180238	7	*TFPI2*	5.7E-13	-	-	-	-	-	0
rs4899412, rs2052015, rs2529471, rs36423	14	*RGS6*	-	-	-	0.69	0.43		1
rs6123471	20	*KIAA1755*	-	-	-		0.71	-	1
rs7980799	12	*SYT10*	2.7E-13	0.02	0.16	-	-	-	1

* Caution should be exercised when using the *p*-value as a measure of confidence that the highlighted genes are indeed causal for the associations identified by GWAS, or as a means to compare evidence across tools. Each tool uses different databases and algorithms to generate these *p*-values and as such, *p*-values cannot necessarily be compared across tools. We advise to use the presence of a *p*-value as an indication that the respective tool identifies a gene as potentially being causal.

# DEPICT is considered to be the most advanced tool since it uses the most up-to-date information to derive which genes are likely to be causal. For DEPICT, only identified genes that were located within ±40 kb of GWAS lead SNPs are reported.

Supplementary Table 20: Network and functional enrichment analyses.

Gene ontology (GO) terms related to cell membrane signal transduction are highlighted in yellow; GO terms related to cellular anabolic, catabolic, and respiratory processes are highlighted in green. Significant false discovery rate *q*-values are shown in red font, suggestive ones in purple.

GO id	Description	*q*-value	Occurrences in Sample	Occurrences in Genome
GO:0071377	cellular response to glucagon stimulus	3.01E-13	10	33
GO:0033762	response to glucagon	3.01E-13	10	33
GO:0036065	fucosylation	1.90E-11	7	11
GO:0008417	fucosyltransferase activity	7.35E-11	7	13
GO:0006004	fucose metabolic process	1.67E-08	6	13
GO:0006112	energy reserve metabolic process	5.55E-07	10	143
GO:0015980	energy derivation by oxidation of organic compounds	3.00E-05	11	285
GO:0071375	cellular response to peptide hormone stimulus	7.32E-05	10	244
GO:1901653	cellular response to peptide	7.32E-05	10	247
GO:0043434	response to peptide hormone	8.89E-05	10	255
GO:1901652	response to peptide	9.70E-05	10	260
GO:0043413	macromolecule glycosylation	1.65E-04	9	209
GO:0070085	glycosylation	2.02E-04	9	216
GO:0016758	transferase activity, transferring hexosyl groups	3.49E-04	7	113
GO:0019320	hexose catabolic process	4.23E-04	6	72
GO:0046365	monosaccharide catabolic process	4.67E-04	6	74
GO:0003924	GTPase activity	1.01E-03	7	136
GO:0006486	protein glycosylation	1.39E-03	8	208
GO:0008277	regulation of G-protein coupled receptor protein signaling pathway	1.53E-03	6	93
GO:0044724	single-organism carbohydrate catabolic process	1.86E-03	6	97
GO:0016757	transferase activity, transferring glycosyl groups	2.15E-03	7	157
GO:0016052	carbohydrate catabolic process	2.27E-03	6	102
GO:0009101	glycoprotein biosynthetic process	6.01E-03	8	262
GO:0031305	integral component of mitochondrial inner membrane	1.21E-02	3	13
GO:0031304	intrinsic component of mitochondrial inner membrane	1.47E-02	3	14
GO:0006626	protein targeting to mitochondrion	2.74E-02	4	49
GO:0070585	protein localization to mitochondrion	3.09E-02	4	51
GO:0019318	hexose metabolic process	4.84E-02	6	182
GO:0005834	heterotrimeric G-protein complex	5.25E-02	3	22
GO:0005996	monosaccharide metabolic process	9.49E-02	6	209
GO:0030695	GTPase regulator activity	9.49E-02	5	132

SUPPLEMENTARY NOTES

1. Gene-based GWAS (VEGAS)

The results of the VEGAS gene-based GWAS analysis corroborate those of the SNP-based analysis (Supplementary Table 17). In seven of our eight loci with genome-wide significant SNPs (Table 1) the closest gene or other nearby genes also emerged from VEGAS as significantly associated with at least one of the HRV traits. Only the loci on chromosome 6 (*PPIL*) and 15 (*NEO1*) were not represented. In addition, a few new genes close to the top hits in our SNP-based analyses emerged: *TFPI2* and *GNGT1* (both on chromosome 7), *ALG10*, *ALG10B*, and *CPNE8* (all on chromosome 12), and *NRTN*, *FUT6*, *FUT3*, *FUT5*, *VMAC*, *CAPS*, *RANBP3*, and *RFX2* (all on chromosome 19). Only one gene in a new locus was identified: *CCDC141* (on chromosome 2, next to *TTN* coding for the titin protein, a key determinant of myocardial passive stiffness). *CCDC141* and *CPNE8* have previously been reported to be associated in a GWAS for heart rate[14].

2. Heritability and genetic correlations

The common SNP heritability estimated by Genomic Restricted Maximum Likelihood (GREML) analysis was 10.8% and 13.2% for SDNN and RMSSD in the Lifelines cohort (Supplementary Table 18, panel a). Applying LD score regression analysis on the summary statistics of the stage 1 meta-analysis suggested a common SNP heritability of 11.1% for SDNN, 11.2% for RMSSD, and 11.8% for pvRSA/HF (Supplementary Table 18, panel b). Classical modeling on family data from the Oman Family Study yielded heritability estimates between 15.2 and 20.0%.

Previous studies showed high phenotypic correlation between pvRSA/HF and RMSSD[31, 32] and suggested a large overlap in their genetic architecture[33, 34]. We confirmed the phenotypic correlations in the TRAILS-Pop, NESDA, and Lifelines cohorts where they ranged from 0.70 (SDNN-pvRSA/HF) to 0.96 (RMSSD-pvRSA/HF). Bivariate analysis using GREML and LD score regression analysis further showed genetic correlations of unity between pvRSA/HF and RMSSD, but also with SDNN, indicating complete overlap between the genetic variants influencing all three HRV traits (Supplementary Table 18, panel a+b). Modeling of the family data from the Oman Family Study confirmed high genetic correlation between the HRV traits, including SDNN (0.71-0.90) (Supplementary Table 18, panel c). This shows that SDNN, notwithstanding its different etiology, is influenced by sets of genetic variants that also influence the RMSSD and pvRSA/HF traits.

We observed large negative genetic correlations (between -0.74 and -0.55) of SDNN, RMSSD, and pvRSA/HF with heart rate (Supplementary Table 11, panel b). High genetic overlap between heart rate and HRV fits the notion that HRV increases with a net increase of the tonic vagal effects on the sinoatrial node, which in turn reduces average heart rate. However, we note that the full relationship between HRV and heart rate is more complex because heart rate is under parallel sympathetic control, changes in which are not captured well by the HRV measures used [35, 36] and because changes in heart rate can lead to lower HRV, even at unchanged vagal input [37].

3. Association between heart rate SNPs and HRV

After establishing the effects of HRV SNPs on the variance in heart rate we also examined the reverse question, i.e. whether SNPs known to be associated with heart rate accounted for part of the variance in HRV. The reverse association of the 21 heart rate SNPs identified by a GWAS

study for heart rate[14] with the three HRV traits showed that nine heart rate SNPs were associated with HRV, all in the expected direction, after correction for multiple testing (Supplementary Table 10, panel a)). Using *gtx*, a multi-SNP genetic risk scores for heart rate was associated with the three HRV traits (Supplementary Table 10, panel b).

In the Lifelines, NESDA, TRAILS-Pop, and ABCD cohorts, a multi-SNP genetic risk score for heart rate explained 0.2 to 0.9% of HRV variance in the four cohorts (Supplementary Table 10, panel c). The full polygenic risk score for heart rate explained a similar amount of the variance in the three HRV traits: 0.2 to 0.8% (Supplementary Table 10, panel d).

4. Network and functional enrichment analyses

Based on our 17 HRV SNPs, we selected candidate genes as input for the interaction network analysis with the GeneMANIA algorithm to prioritize potentially causal genes [38] with additional input from the VEGAS analyses (Supplementary Table 17) and four publicly available bioinformatics tools (Supplementary Table 19). As one of the genes (*LINC00477*) could not be found by GeneMANIA, 24 query genes were used as input. Functional network and enrichment analysis on these genes resulted in 31 significantly enriched gene ontology terms of which 23 had false discovery rate ≤ 0.01 (Supplementary Table 20). These 23 gene ontology terms showed that the genes near our hits were broadly related to two categories of significantly enriched biological processes, namely: (1) cellular signaling and cellular responses, including G-protein coupled receptor protein signaling and the responses to glucagon and peptides, and (2) metabolic processes in the cell (e.g. fucosylation and glycosylation) (Supplementary Fig. 10).

5. Tissue and gene-set enrichment analyses

In silico tissue enrichment analysis using DEPICT[6] highlights a role for hormones in HRV regulation, with enrichment for the adrenal cortex, endocrine glands, gonads, gastrointestinal tract and female reproductive organs (Supplementary Fig. 8a,c,e). Gene-set enrichment analysis using DEPICT highlights the importance of cardiac development (Supplementary Fig. 8b,d,f).

6. Known biological functions of the genes closest to the HRV SNPs

Here we describe the known biological functions of the genes closest to the eight HRV lead SNPs identified in the two-stage meta-analysis.

NDUFA11 (Chr 19, full name: NADH dehydrogenase (ubiquinone) 1 alpha sub-complex, 11, also known as B14.7 and Cl-B14.7) encodes a subunit of the membrane-bound mitochondrial complex I involved in mitochondrial respiration and electron transport. NDUFA11's cDNA was cloned and sequenced from human heart mitochondria. The non-synonymous lead SNP in our meta-analysis induces a change in the structure of this complex with as yet unknown functional effects. Clinically, five phenotypes are frequently seen with homozygous mutations of this gene: severe neonatal lactic acidosis, cardiomyopathy-encephalopathy, hepatopathy-tubulopathy, leukodystrophy with macrocephaly, and Leigh's and Leigh-like neurodegenerative disorder, which are the most common presentations of complex I deficiency at a young age [39, 40].
LINC00477 (C12orf67) (Chr 12, full name: long intergenic non-protein coding RNSA 477) is of unknown biological function but the locus has also been associated with resting heart rate [14] and PR interval [41].

PPIL1 (Chr 6, full name: peptidylprolyl isomerase (cyclophilin)-like 1) is a member of the cyclophilin family. After being recruited by Ski interaction protein (SKIP) PPIL1 participates in the activation of spliceosomes and thereby facilitates the folding of proteins in the spliceosomes [42, 43]. No relation with heart rate or HRV has been described but *PPIL1* has been reported to be one of two key driver genes involved in coronary artery disease networks [44].
SYT10 (Chr 12, full name: synaptotagmin-10) has a role in the regulation of calcium dependent exocytosis, including calcium regulated release of neurotransmitter from presynaptic nerve terminals [45, 46]. *SYT10* has been previously identified as being associated with heart rate [14]. Synaptotagmin-1 plays a known role in calcium-triggered acetylcholine release from the neuromuscular junction [47]. Our meta-analyses results hint at a possible role for synaptotagmin-10 in sinoatrial acetylcholine release but this remains to be tested.
GNG11 (Chr 7, full name: guanine nucleotide binding protein (G protein, gamma 11) which is also a previously identified heart rate gene [14] encodes the γ11 subunit of Gαβγ heterotrimers. *GNG11* is one of 12 genes encoding the Gγ subunits that all undergo post-translational isoprenylation of their C termini, in case of γ11 by a farnesyl [48]. Because the γ subunits have lower amino acid sequence homology then the β subunits, they are thought to be the main determinant of signaling diversity and fidelity of the Gβγ components [49, 50]. γ11 shows a unique pattern of expression compared to the other 11 γ subunits in that it is abundantly expressed in the heart but poorly in the brain[50].
RGS6 (Chr 14, full name: regulator of G-protein signaling 6) is a large gene coding one of the RGS superfamily that act as GTPase-activating proteins (GAPs) for the α subunit of Gαβγ heterotrimers by accelerating their intrinsic GTPase activity. This ends both Gα and Gβγ mediated cellular signaling. RGS6 exhibits a uniquely robust expression in the heart [10]. RGS6 is known to reduce parasympathetic signaling through the M_2R in the sinoatrial node as well as $G\alpha_o$ coupled adenosine A1 receptors [10, 51, 52].
HCN4 (Chr 15, full name: hyperpolarization activated cyclic nucleotide gated potassium channel 4) codes for a non-GIRK potassium channel in the sinoatrial node that is a core part of the 'clock circuit' generating the pacemaker potential. *HCN4* is expressed abundantly in the heart from the early embryonic phase onward [12]. *HCN4* was previously found to be associated with heart rate [14]. Depending on total load and severity, loss-of-function mutations in *HCN4* can lead to asymptomatic bradycardia, Brugada syndrome and Sick Sinus Syndrome, atrial fibrillation and possibly left ventricular noncompaction cardiomyopathy [53, 54].
NEO1 (Chr 15, full name: neogenin 1, aka *HsT16534*, *IGDCC2*, *NGN* or *NTN1R2*) is a multifunctional transmembrane receptor closely related to the immunoglobulin (Ig) superfamily and is a netrin receptor [55]. Neogenin plays a role in early cerebellar neurite out-growth and projection in chicken and quail [56, 57] and is known to be a regulator of axonal guidance in the nervous system [58]. No role in HRV or heart rate has been described previously.
KIAA1755 (Chr 20, no full name in Ensembl or at NCBI) which has also been associated with heart rate [14]. Effects of the non-synonymous lead SNP (rs6123471) is unclear as the function of this gene remains unknown.

7. Software and internet databases used

Annovar	URL: http://annovar.openbioinformatics.org/en/latest/
DEPICT	URL: http://www.broadinstitute.org/mpg/depict/
Endeavour	URL: http://homes.esat.kuleuven.be/~bioiuser/endeavour/index.php
GeneMANIA	URL: http://genemania.org/
Genome-wide Complex Trait Analysis (GCTA) version 1.24.4	URL: http://cnsgenomics.com/software/gcta/
gtx (R package)	URL: https://cran.r-project.org/web/packages/gtx/index.html
GWAMA	URL: http://www.well.ox.ac.uk/gwama/
LD score regression	URL: https://github.com/bulik/ldsc
LocusZoom	URL: http://locuszoom.sph.umich.edu/locuszoom/
METAL	URL: http://csg.sph.umich.edu//abecasis/Metal/index.html
MetaRanker	URL: http://www.cbs.dtu.dk/services/MetaRanker/
PriorityPruner version 0.1.1	URL: http://prioritypruner.sourceforge.net/
PLINK version 1.07	URL: http://pngu.mgh.harvard.edu/purcell/plink/
PolyPhen	URL: http://genetics.bwh.harvard.edu/pph2/
RegulomeDB	URL: http://regulomedb.org/
SIFT	URL: http://sift.jcvi.org/
SNP annotation and proxy search (SNAP)	URL: https://www.broadinstitute.org/mpg/snap/
SOLAR	URL: http://solar-eclipse-genetics.org/
ToppGene	URL: https://toppgene.cchmc.org/

8. Acknowledgments, study consent, and funding

Stage 1 cohorts

ARIC: We thank the staff and participants of the ARIC study for their important contributions. The Atherosclerosis Risk in Communities Study is carried out as a collaborative study supported by National Heart, Lung, and Blood Institute contracts (HHSN268201100005C, HHSN268201100006C, HHSN268201100007C, HHSN268201100008C, HHSN268201100009C, HHSN268201100010C, HHSN268201100011C, and HHSN268201100012C), R01HL087641, R01HL59367 and R01HL086694; National Human Genome Research Institute contract U01HG004402; and National Institutes of Health contract HHSN268200625226C. Infrastructure was partly supported by Grant Number UL1RR025005, a component of the National Institutes of Health and NIH Roadmap for Medical Research. The Atherosclerosis Risk in Communities (ARIC) Study (#11-0734) was approved by the Biomedical Institutional Review Board (IRB) at the University of North Carolina (Chapel Hill, NC).

CHS: We thank the contributing CHS investigators and institutions, a list of principal contributors can be found at CHS-NHLBI.org. CHS research was supported by NHLBI contracts

HHSN268201200036C, HHSN268200800007C, N01HC55222, N01HC85079, N01HC85080, N01HC85081, N01HC85082, N01HC85083, N01HC85086; and NHLBI grants U01HL080295, R01HL087652, R01HL105756, R01HL103612, R01HL120393, R01HL062181, and R01HL130114, with additional contribution from the National Institute of Neurological Disorders and Stroke (NINDS). Additional support was provided through R01AG023629 from the National Institute on Aging (NIA). The provision of genotyping data was supported in part by the National Center for Advancing Translational Sciences, CTSI grant UL1TR000124, and the National Institute of Diabetes and Digestive and Kidney Disease Diabetes Research Center (DRC) grant DK063491 to the Southern California Diabetes Endocrinology Research Center. The content is solely the responsibility of the authors and does not necessarily represent the official views of the National Institutes of Health.
Written informed consent was obtained from all Cardiovascular Health Study participants. The study was approved by the Institutional Review Boards of the University of Washington, Seattle, WA, USA; the University of California, Davis, CA, USA; the Johns Hopkins School of Public Health, Baltimore, MD, USA; the University of Pittsburgh, Pittsburgh, PA, USA; the Institutional Review Board in the Office of Research, Wake Forest University, Winston-Salem, NC, USA; and the Committee on Human Subjects of the University of Vermont, Burlington, VT, USA. Current approval at the University of Washington IRB runs through 12/12/17.

FHS: The National Heart, Lung and Blood Institute's Framingham Heart Study is supported by contract N01-HC-25195 and HHSN268201500001I.
The FHS study protocol was approved by the Institutional Review Board of the Boston University Medical Center and all participants gave written informed consent.

FINGESTURE: Is supported by the Sigrid Juselius Foundation and the Finnish Foundation for Cardiovascular Research, Helsinki, Finland. The genotyping of this cohort was supported by the Montreal Heart Institute Foundation.
Written informed consent was obtained from all FINGESTURE participants. The study was approved by the Regional Ethics Committee of the Northern Ostrobothnia Hospital District, Oulu, Finland (IRB number 21/2007).

FLEMENGHO-EPOGH: Nuclear families were recruited in the framework of the European Project On Genes in Hypertension, which was supported by the European Union (contract numbers IC15-CT98-0329-EPOGH and QLGI-CT-2000-01137-EURNETGEN). The study was also supported by research grants G.0174.97, G.0291.98, and G.0424.03 from the Fonds voor Wetenschappelijk Onderzoek Vlaanderen (Brussels, Belgium); by a special research grant (Onderzoekstoelage OT/99/28) from the Katholieke Universiteit Leuven (Leuven, Belgium); by research grants (OK 375 and OK 376) from the Czech Ministry of Education; and by the International Scientific Collaboration between Poland and Flanders (contract number BIL 00/18). Both the FLEMENGHO and EPOGH studies were conducted according to the principles outlined in the Helsinki Declaration for Investigation of Human Participants. Each local institutional review board approved the study protocol. Participants provided written informed consent.

GenR: We gratefully acknowledge the contribution of general practitioners, hospitals, midwives and pharmacies in Rotterdam to The Generation R Study. GenR is conducted by the Erasmus Medical Center Rotterdam in close collaboration with the Faculty of Social Sciences of the Erasmus University Rotterdam, the Municipal Health Service Rotterdam area, the Rotterdam

Homecare Foundation and the Stichting Trombosedienst & Artsenlaboratorium Rijnmond (STAR), Rotterdam.
Written informed consent was obtained from mothers and fathers of all participants. The Medical Ethical Committee of the Erasmus Medical Centre, Rotterdam (MEC-2007-413-NL21545.078; MEC 198.782.2001.31; MEC 217.595/2002/202; MEC-2007-413, MEC-2012-165) approved the study protocol.
GTR: The sample for the Twin Interdisciplinary Neuroticism Study (TWINS) was selected from the Groningen Twin Register (GTR). TWINS was supported by the Netherlands Organization for Health Research and Development (ZonMw 904-57-130), and the UK-Netherlands Partnership Program in Science (BR 56-481 and BR 96-229), which is jointly run and financed by the British Council and the Netherlands Organization for Scientific Research (NWO).
The GTR study was approved by the Ethics Committee of the University Medical Center Groningen (METc 2000/060e), and all subjects gave written consent prior to participation.

KORA: The KORA study was initiated and financed by the Helmholtz Zentrum München – German Research Center for Environmental Health, which is funded by the German Federal Ministry of Education and Research (BMBF) and by the State of Bavaria. Furthermore, KORA research was supported within the Munich Center of Health Sciences (MC-Health), Ludwig-Maximilians-Universität, as part of LMUinnovativ. Statistical analyses were carried out on the Genetic Cluster Computer (http://www.geneticcluster.org) hosted by SURFsara and financially supported by the Netherlands Scientific Organization (NWO 480-05-003) along with a supplement from the Dutch Brain Foundation and the VU University Amsterdam.
All participants of KORA S4 provided written informed consent for participation in the study, which was approved by the Ethics Committee of the Bavarian Medical Association (#99186).

MESA: We thank the other investigators, the staff, and the participants of the MESA study for their valuable contributions. A full list of participating MESA investigators and institutions can be found at http://www.mesa-nhlbi.org. MESA and the MESA SHARe project are conducted and supported by the National Heart, Lung, and Blood Institute (NHLBI) in collaboration with MESA investigators. Support for MESA is provided by contracts HHSN268201500003I, N01-HC-95159, N01-HC-95160, N01-HC-95161, N01-HC-95162, N01-HC-95163, N01-HC-95164, N01-HC-95165, N01-HC-95166, N01-HC-95167, N01-HC-95168, N01-HC-95169, UL1-TR-001079, UL1-TR-000040, and DK063491. Funding for SHARe genotyping was provided by NHLBI Contract N02-HL-64278. Genotyping was performed at Affymetrix (Santa Clara, California, USA) and the Broad Institute of Harvard and MIT (Boston, Massachusetts, USA) using the Affymetrix Genome-Wide Human SNP Array 6.0. This publication was developed under a STAR research assistance agreement, No. RD831697 (MESA Air), awarded by the U.S Environmental protection Agency. It has not been formally reviewed by the EPA. The views expressed in this document are solely those of the authors and the EPA does not endorse any products or commercial services mentioned in this publication.
Written informed consent was obtained from all MESA participants. The study was approved by the Institutional Review Board at each field center and the data coordinating center. Each Institutional Review Board is certified by the U.S. Office of Human Research Protections.

MRS: We thank the MRS study team and the 1st Marine Division and Navy Medicine at 29 Palms and at Camp Pendleton. Funding for this study was provided by NIH grant 1 R01MH093500 (to CMN). The Marine Corps and Navy Bureau of Medicine and Surgery

(BUMED) and VA Health Services and Development (HSR&D) provided funding for MRS-I and MRS-II (DGB, VBR, CMN, AM, MAG, AXM).
Written informed consent was obtained from all MRS participants. The study was approved by the University of California-San Diego Institutional Review Board (IRB number 150563).
NESDA: Funding was obtained from the Netherlands Organization for Scientific Research (Geestkracht program grant 10-000-1002); the Center for Medical Systems Biology (CSMB, NOW Genomics), Biobanking and Biomolecular Resources Research Infrastructure (BBMRI-NL), VU University's Institutes for Health and Care Research (EMGO+) and Neuroscience Campus Amsterdam, University Medical Center Groningen, Leiden University Medical Center, National Institutes of Health (NIH, R01D0042157-01A, MH081802, Grand Opportunity grants 1RC2 MH089951 and 1RC2 MH089995). Part of the genotyping and analyses were funded by the Genetic Association Information Network (GAIN) of the Foundation for the National Institutes of Health. Computing was supported by BiG Grid, the Dutch e-Science Grid, which is financially supported by NWO. Part of the statistical analyses were carried out on the Genetic Cluster Computer (http://www.geneticcluster.org) hosted by SURFsara and financially supported by the Netherlands Scientific Organization (NWO 480-05-003) along with a supplement from the Dutch Brain Foundation and the VU University Amsterdam.
Written informed consent was obtained from all NESDA participants. The study was approved by the Central Ethics Committee on Research Involving Human Subjects of the VU University Medical Centre, Amsterdam, an Institutional Review Board certified by the U.S. Office of Human Research Protections (IRB number IRB00002991 under Federal-wide Assurance-FWA00017598; IRB/institute code 03-183).

NTR, Netherland Twin Register: Funding was obtained from the Netherlands Organization for Scientific Research (NWO) and The Netherlands Organization for Health Research and Development (ZonMW) grants 904-61-090, 985-10-002, 912-10-020, 904-61-193,480-04-004, 463-06-001, 451-04-034, 400-05-717, Addiction-31160008, Middelgroot-911-09-032, Spinozapremie 56-464-14192, Biobanking and Biomolecular Resources Research Infrastructure (BBMRI –NL, 184.021.007). VU Institute for Health and Care Research (EMGO+); the European Community's Seventh Framework Program (FP7/2007-2013), ENGAGE (HEALTH-F4-2007-201413); the European Research Council (ERC Advanced, 230374, ERC Starting grant 284167), Rutgers University Cell and DNA Repository (NIMH U24 MH068457-06), the Avera Institute, Sioux Falls, South Dakota (USA) and the National Institutes of Health (NIH, R01D0042157-01A, MH081802; R01 DK092127-04, Grand Opportunity grants 1RC2 MH089951 and 1RC2 MH089995). Part of the genotyping and analyses were funded by the Genetic Association Information Network (GAIN) of the Foundation for the National Institutes of Health. Computing was supported by BiG Grid, the Dutch e-Science Grid, which is financially supported by NWO.
Written informed consent was obtained from all NTR participants. NTR studies were approved by the Central Ethics Committee on Research Involving Human Subjects of the VU University Medical Centre, Amsterdam, an Institutional Review Board certified by the U.S. Office of Human Research Protections (IRB number IRB00002991 under Federal-wide Assurance-FWA00017598; IRB/institute codes, NTR 03-180).

PIVUS: We thank the SNP&SEQ Technology Platform (www.genotyping.se), which is part of the National Genomics Infrastructure hosted by Science for Life laboratory at Uppsala University for excellent genotyping. This project was supported by Knut and Alice Wallenberg Foundation

(Wallenberg Academy Fellow), European Research Council (ERC Starting Grant), Swedish Diabetes Foundation (2013-024), Swedish Research Council (2012-1397, 2012-1727, and 2012-2215), Marianne and Marcus Wallenberg Foundation, County Council of Dalarna, Dalarna University, and Swedish Heart-Lung Foundation (20120197). The computations were performed on resources provided by SNIC through Uppsala Multidisciplinary Center for Advanced Computational Science (UPPMAX) under Project b2011036. Genotyping was funded by the Wellcome Trust under award WT064890. Analysis of genetic data was funded by the Wellcome Trust under awards WT098017 and WT090532.
Written informed consent was obtained from all participants of the PIVUS study. The protocols were approved by the Ethics Committee of Uppsala University.

PREVEND: PREVEND genetics is supported by the Dutch Kidney Foundation (Grant E033), the EU project grant GENECURE (FP-6 LSHM CT 2006 037697), the National Institutes of Health (grant 2R01LM010098), The Netherlands organization for health research and development (NWO-Groot grant 175.010.2007.006, NWO VENI grant 916.761.70, ZonMw grant 90.700.441), and the Dutch Inter University Cardiology Institute Netherlands (ICIN).
All participants of the PREVEND study provided informed consent. This study has been approved by the review board of the University Medical Center Groningen. This study adheres to the principles expressed in the Declaration of Helsinki.

RS: We are grateful to the study participants, the staff from the Rotterdam Study and the participating general practitioners and pharmacists. Furthermore, we thank Pascal Arp, Mila Jhamai, Marijn Verkerk, Lizbeth Herrera and Marjolein Peters, MSc, and Carolina Medina-Gomez, MSc, for their help in creating the GWAS database, and Karol Estrada, PhD, Yurii Aulchenko, PhD, and Carolina Medina-Gomez, MSc, for the creation and analysis of imputed data. The generation and management of GWAS genotype data for the Rotterdam Study (RS I, RS II) was executed by the Human Genotyping Facility of the Genetic Laboratory of the Department of Internal Medicine, Erasmus MC, Rotterdam, The Netherlands. The GWAS datasets are supported by the Netherlands Organisation of Scientific Research NWO Investments (nr. 175.010.2005.011, 911-03-012), the Genetic Laboratory of the Department of Internal Medicine, Erasmus MC, the Research Institute for Diseases in the Elderly (014-93-015; RIDE2), the Netherlands Genomics Initiative (NGI)/Netherlands Organization for Scientific Research (NWO) Netherlands Consortium for Healthy Aging (NCHA), project nr. 050-060-810. The Rotterdam Study is funded by Erasmus Medical Center and Erasmus University, Rotterdam, Netherlands Organization for the Health Research and Development (ZonMw), the Research Institute for Diseases in the Elderly (RIDE), the Ministry of Education, Culture and Science, the Ministry for Health, Welfare and Sports, the European Commission (DG XII), and the Municipality of Rotterdam.
The Rotterdam study has been approved by the Medical Ethics Committee of the Erasmus MC and by the Ministry of Health, Welfare and Sport of the Netherlands, on the basis of the Wet Bevolkingsonderzoek ERGO. All participants provided written informed consent.

TRAILS: This research is part of the TRacking Adolescents' Individual Lives Survey (TRAILS), which includes the TRAILS population cohort (TRAILS-Pop) and the TRAILS clinical cohort (TRAILS-CC). Participating centers of TRAILS include the University Medical Center and University of Groningen, the Erasmus University Medical Center Rotterdam, the University of

Utrecht, the Radboud Medical Center Nijmegen, and the Parnassia Bavo group, all in the Netherlands. We are grateful to everyone who participated in this research or worked on this project to make it possible. TRAILS has been financially supported by various grants from the Netherlands Organization for Scientific Research NWO (Medical Research Council program grant GB-MW 940-38-011; ZonMW Brainpower grant 100-001-004; ZonMw Risk Behavior and Dependence grants 60-60600-97-118; ZonMw Culture and Health grant 261-98-710; Social Sciences Council medium-sized investment grants GB-MaGW 480-01-006 and GB-MaGW 480-07-001; Social Sciences Council project grants GB-MaGW 452-04-314 and GB-MaGW 452-06-004; NWO large-sized investment grant 175.010.2003.005; NWO Longitudinal Survey and Panel Funding 481-08-013 and 481-11-001), the Dutch Ministry of Justice (WODC), the European Science Foundation (EuroSTRESS project FP-006), Biobanking and Biomolecular Resources Research Infrastructure BBMRI-NL (CP 32), and the participating universities. Part of statistical analyses were carried out on the Genetic Cluster Computer (http://www.geneticcluster.org) hosted by SURFsara and financially supported by the Netherlands Scientific Organization (NWO 480-05-003) along with a supplement from the Dutch Brain Foundation and the VU University Amsterdam.
TRAILS was approved by Dutch Central Committee on Research Involving Human Subjects (CCMO); www.ccmo.nl. If both parents and children agreed to participate, parental written informed consent was obtained after the procedures had been fully explained.

ULSAM: We thank the SNP&SEQ Technology Platform (www.genotyping.se), which is part of the National Genomics Infrastructure hosted by Science for Life laboratory at Uppsala University for excellent genotyping. This project was supported by Knut and Alice Wallenberg Foundation (Wallenberg Academy Fellow), European Research Council (ERC Starting Grant), Swedish Diabetes Foundation (2013-024), Swedish Research Council (2012-1397, 2012-1727, and 2012-2215), Marianne and Marcus Wallenberg Foundation, County Council of Dalarna, Dalarna University, and Swedish Heart-Lung Foundation (20120197). The computations were performed on resources provided by SNIC through Uppsala Multidisciplinary Center for Advanced Computational Science (UPPMAX) under Project b2011036. Genotyping was funded by the Wellcome Trust under award WT064890. Analysis of genetic data was funded by the Wellcome Trust under awards WT098017 and WT090532. Andrew P Morris is a Wellcome Trust Senior Fellow in Basic Biomedical Science (award WT098017).
Written informed consent was obtained from all ULSAM participants. The study was approved by the ethics committee at Uppsala University (IRB numbers 251/90 and 2013/350).

YFS: We gratefully acknowledge the expert technical assistance in the statistical analyses by Irina Lisinen. The Young Finns Study has been financially supported by the Academy of Finland: grants 286284, 134309 (Eye), 126925, 121584, 124282, 129378 (Salve), 117787 (Gendi), and 41071 (Skidi); the Social Insurance Institution of Finland; Kuopio, Tampere and Turku University Hospital Medical Funds (grant X51001); Juho Vainio Foundation; Paavo Nurmi Foundation; Finnish Foundation of Cardiovascular Research ; Finnish Cultural Foundation; Tampere Tuberculosis Foundation ; Emil Aaltonen Foundation ; and Yrjö Jahnsson Foundation.
The Young Finns Study was approved by the local ethics committees (University Hospitals of Helsinki, Turku, Tampere, Kuopio and Oulu) and was conducted following the guidelines of the Declaration of Helsinki. All participants gave their written informed consent.

Stage 2 cohorts
CARLA: This study was funded by a grant from the Deutsche Forschungsgemeinschaft (DFG, German Research Foundation) as part of the Collaborative Research Center 598 ‘‘Heart failure in the elderly–cellular mechanisms and therapy’’ at the Medical Faculty of the Martin-Luther-University Halle-Wittenberg; by a grant of the Wilhelm-Roux Programme of the Martin-Luther-University Halle-Wittenberg; by the Federal Employment Office; and by the Ministry of Education and Cultural Affairs of Saxony-Anhalt.
Written informed consent was obtained from all CARLA participants. The study was approved by the Ethics Committee of The Medical Faculty of the Martin-Luther University Halle-Wittenberg and by the State Data Privacy Commissioner of Saxony-Anhalt, Germany.

FINCAVAS: We thank the staff of the Department of Clinical Physiology for collecting the exercise test data. The Finnish Cardiovascular Study (FINCAVAS) has been financially supported by the Competitive Research Funding of the Tampere University Hospital (Grant 9M048 and 9N035), the Finnish Cultural Foundation, the Finnish Foundation for Cardiovascular Research, the Emil Aaltonen Foundation, Finland, and the Tampere Tuberculosis Foundation. The FINCAVAS study protocol was approved by the Ethical Committee of the Hospital District of Pirkanmaa, Finland, and all patients have given informed consent prior to the interview and measurements as stipulated in the Declaration of Helsinki.

HANDLS: The Healthy Aging in Neighborhoods of Diversity across the Life Span (HANDLS) study was supported by the Intramural Research Program of the NIH, National Institute on Aging and the National Center on Minority Health and Health Disparities (project # Z01-AG000513 and human subject’s protocol number 09-AG-N248). We thank the HANDLS study participants, staff and field workers for their contribution to this work. Data analyses for the HANDLS study utilized the high-performance computational resources of the Biowulf Linux cluster at the National Institutes of Health, Bethesda, MD, (http://hpc.nih.gov).
HANDLS is reviewed and approved by the National Institute of Environmental Health Sciences Institutional Review Board at NIH

HCHS/SOL: We thank the staff and participants of HCHS/SOL for their important contributions. A complete list of staff and investigators is available on the study website http://www.cscc.unc.edu/hchs/. The Hispanic Community Health Study/Study of Latinos was carried out as a collaborative study supported by contracts from the National Heart, Lung, and Blood Institute (NHLBI) to the University of North Carolina (N01-HC65233), University of Miami (N01-HC65234), Albert Einstein College of Medicine (N01-HC65235), Northwestern University (N01-HC65236), and San Diego State University (N01-HC65237). The following Institutes/Centers/Offices contribute to the HCHS/SOL through a transfer of funds to the NHLBI: National Center on Minority Health and Health Disparities, the National Institute of Deafness and Other Communications Disorders, the National Institute of Dental and Craniofacial Research, the National Institute of Diabetes and Digestive and Kidney Diseases, the National Institute of Neurological Disorders and Stroke, and the Office of Dietary Supplements. The Genetic Analysis Center at University of Washington was supported by NHLBI and NIDCR contracts (HHSN268201300005C AM03 and MOD03). Genotyping efforts were supported by NHLBI HSN 26220/20054C, NCATS CTSI grant UL1TR000124, and NIDDK Diabetes Research Center (DRC) grant DK063491.

Written informed consent was provided by all HCHS/SOL participants; IRB approvals were obtained by all field sites recruiting participants: Bronx Site at Albert Einstein College of Medicine, Yeshiva University (FWA00000140) and Montifiore Medical Center (FWA0000258); Chicago Site at University of Illinois, Chicago (FWA00000083, IRB00000117 IRB#3); San Diego Site at San Diego State University: (IORG0000333, IRB00000576); Miami site at University of Miami, Florida (FWA00002247).

Lifelines: We wish to acknowledge the services of the Lifelines Cohort Study, the contributing research centers delivering data to Lifelines, and all the study participants. The Lifelines Cohort Study, and generation and management of GWAS genotype data for the Lifelines Cohort Study is supported by the Netherlands Organization of Scientific Research NWO (grant 175.010.2007.006), the Economic Structure Enhancing Fund (FES) of the Dutch government, the Ministry of Economic Affairs, the Ministry of Education, Culture and Science, the Ministry for Health, Welfare and Sports, the Northern Netherlands Collaboration of Provinces (SNN), the Province of Groningen, University Medical Center Groningen, the University of Groningen, Dutch Kidney Foundation and Dutch Diabetes Research Foundation. Part of the statistical analyses were carried out on the Genetic Cluster Computer (http://www.geneticcluster.org) hosted by SURFsara and financially supported by the Netherlands Scientific Organization (NWO 480-05-003) along with a supplement from the Dutch Brain Foundation and the VU University Amsterdam.
All participants signed an informed consent form before they received an invitation for the physical examination. The LifeLines Cohort Study is conducted according to the principles of the Declaration of Helsinki and in accordance with research code University Medical Center Groningen (UMCG). The LifeLines study is approved by the medical ethical committee of the UMCG, the Netherlands (METc 2007/152).

MRC NSHD: We are very grateful to the members of this birth cohort for their continuing interest and participation in the study. We would like to acknowledge the Swallow group, UCL, who performed the DNA extractions (Rousseau, et al 2006). DOI: 10.1111/j.1469-1809.2006.00250.x MRC NSHD was funded by the Medical Research Council (MC_UU_12019/1); doi: 10.5522/NSHD/Q102.
Written informed consent was obtained from all NSHD study members. Ethical approval was given by the Central Manchester Research Ethics Committee (07/H1008/168, 07/H1008/245) and the Scottish A Research Ethics Committee (08/MRE00/12).

NFBC 1966: We thank the late Professor Paula Rantakallio (launch of NFBCs), and Ms Outi Tornwall, Ms Minttu Jussila (DNA biobanking), Ms Nelli Perkiömäki (HRV analysis), the participants in the 46y study and the NFBC project center. The authors would like to acknowledge the contribution of the late Academian of Science Leena Peltonen. The DNA extractions, sample quality controls, biobank up-keeping and aliquotting was performed in the National Public Health Institute, Biomedicum Helsinki, Finland and supported financially by the Academy of Finland and Biocentrum Helsinki. NFBC1966 received financial support from the Academy of Finland (project grants 104781, 120315, 129269, 1114194, 24300796, 267435, 285547 Center of Excellence in Complex Disease Genetics and SALVE), University of Oulu, Oulu University Hospital and Biocenter, Oulu, Finland [grant numbers 24301140, 24000692, 75617], European Regional Development Fund [grant number 539/2010 A31592], NHLBI grant 5R01HL087679-02 through the STAMPEED program (1RL1MH083268-01), NIH/NIMH

(5R01MH63706:02), ENGAGE project and grant agreement HEALTH-F4-2007-201413, EU FP7 EurHEALTHAgeing -277849, the Medical Research Council, UK (G0500539, G0600705, G1002319, PrevMetSyn/SALVE), the MRC, Centenary Early Career Award, the Sigrid Juselius Foundation and the Finnish Foundation for Cardiovascular Research. The program is currently being funded by the H2020-633595 DynaHEALTH action and the Academy of Finland EGEA-project.
Written informed consent was obtained from all NFBC1966 participants. The study was approved by the Regional Ethics Committee of the Northern Ostrobothnia Hospital District, Oulu, Finland (IRB number 94/2011).

UCSD TWINS: We wish to dedicate this work to the memory Daniel T. O'Connor, whose body of work, particularly with the University of California San Diego Twins cohort, and passion for new knowledge were unprecedented. This study was supported by P30 DK079337 and U01 HL69758-01.
Written informed consent was obtained from all UCSD twin participants. The study was approved by the University of California-San Diego Institutional Review Board (IRB number 120582).

WHI: We thank all contributors to WHI science, which are listed at https://www.whi.org/researchers/Documents%20%20Write%20a%20Paper/WHI%20Investigator%20Long%20List.pdf.
The Women's Health Initiative study was approved by the human subjects review committee at each participating institution and all participants provided written informed consent.

WHI CT – GARNET: The Women's Health Initiative clinical trials were funded by the National Heart, Lung, and Blood Institute, National Institutes of Health, U.S. Department of Health and Human Services through contracts HHSN268201100046C, HHSN268201100001C, HHSN268201100002C, HHSN268201100003C, HHSN268201100004C, and HHSN271201100004C. Within the Genomics and Randomized Trials Network, a GWAS of Hormone Treatment and CVD and Metabolic Outcomes in the WHI was funded by the National Human Genome Research Institute, National Institutes of Health, U.S. Department of Health and Human Services through cooperative agreement U01HG005152 (Reiner).
The Women's Health Initiative study was approved by the human subjects review committee at each participating institution and all participants provided written informed consent.

WHI CT – MOPMAP: The Women's Health Initiative clinical trials were funded by the National Heart, Lung, and Blood Institute, National Institutes of Health, U.S. Department of Health and Human Services through contracts HHSN268201100046C, HHSN268201100001C, HHSN268201100002C, HHSN268201100003C, HHSN268201100004C, and HHSN271201100004C. The Modification of PM-Mediated Arrhythmogenesis in Populations was funded by the National Institute of Environmental Health Sciences, National Institutes of Health, U.S. Department of Health and Human Services through grant R01ES017794 (Whitsel).
The Women's Health Initiative study was approved by the human subjects review committee at each participating institution and all participants provided written informed consent.

WHI - SHARe: The Women's Health Initiative clinical trials were funded by the National Heart, Lung, and Blood Institute, National Institutes of Health, U.S. Department of Health and Human

Services through contracts HHSN268201100046C, HHSN268201100001C, HHSN268201100002C, HHSN268201100003C, HHSN268201100004C, and HHSN271201100004C. The SNP Health Association Resource project was funded by the National Heart, Lung and Blood Institute, National Institutes of Health, U.S. Department of Health and Human Services through contract N02HL64278 (Kooperberg).
The Women's Health Initiative study was approved by the human subjects review committee at each participating institution and all participants provided written informed consent.

WHII: The Whitehall II study is supported by grants from the Medical Research Council (G0902037), British Heart Foundation (RG/07/008/23674), Stroke Association, National Heart Lung and Blood Institute (5RO1 HL036310) and National Institute on Aging (5RO1AG13196 and 5RO1AG034454).
Ethical approval for the Whitehall II study was obtained from University College London Medical School committee on the ethics of human research (reference number 85/0938), and the London-Harrow and Scotland A Research Ethics Committees on Ethics of Human Research. All participants provided written informed consent.

Post-GWAS cohorts and consortia performing post-GWAS analyses and lookups
ABCD: We thank all participating hospitals, obstetric clinics, general practitioners and primary schools for their assistance in implementing the ABCD study. We also gratefully acknowledge all the women and children who participated in this study for their cooperation. The ABCD study has been supported by grants from The Netherlands Organisation for Health Research and Development (ZonMW) and The Netherlands Heart Foundation. Genotyping was funded by the BBMRI-NL grant CP2013-50. Dr M.H. Zafarmand was supported by BBMRI-NL (CP2013-50). Dr. T.G.M. Vrijkotte was supported by ZonMW (TOP 40–00812–98–11010).
All ABCD participants gave written informed consent for themselves and their children. Approval was obtained from the Central Ethics Committee on Research Involving Human Subjects of the Academic Medical Centre (AMC), Amsterdam and the Registration Committee of Amsterdam (IRB number: MEC 02/039#07.17.1039) .

AFGen consortium: See PMID 22544366 for contributors and funding information. Dr. Lubitz is further supported by NIH K23HL114724 and a Doris Duke Charitable Foundation Clinical Scientist Development Award 2014105.

BIOS (Biobank-based Integrative Omics Study) consortium: eQTL and mQTL results were provided by the Biobank-based Integrative Omics Study (BIOS) Consortium, which is funded by the Biobanking and Biomolecular Research Infrastructure (BBMRI-NL, NWO project 184.021.007).
Management Team: Bastiaan T. Heijmans (chair), Peter A.C. 't Hoen, Joyce van Meurs, Aaron Isaacs, Rick Jansen, Lude Franke. Cohort collection: Dorret I. Boomsma, René Pool, Jenny van Dongen, Jouke J. Hottenga (Netherlands Twin Register); Marleen MJ van Greevenbroek, Coen D.A. Stehouwer, Carla J.H. van der Kallen, Casper G. Schalkwijk (Cohort study on Diabetes and Atherosclerosis Maastricht); Cisca Wijmenga, Lude Franke, Sasha V. Zhernakova, Ettje F. Tigchelaar (Lifelines Deep); P. Eline Slagboom, Marian Beekman, Joris Deelen, Diana van Heemst (Leiden Longevity Study); Jan H. Veldink, Leonard H. van den Berg (Prospective ALS Study Netherlands); Cornelia M. van Duijn, Bert A. Hofman, Aaron Isaacs, André G.

Uitterlinden3 (Rotterdam Study). Data Generation Joyce van Meurs (Chair), P. Mila Jhamai, Michael Verbiest, H. Eka D. Suchiman, Marijn Verkerk, Ruud van der Breggen, Jeroen van Rooij, Nico Lakenberg. Data management and computational infra-structure Hailiang Mei (Chair), Maarten van Iterson, Michiel van Galen, Jan Bot, Sasha V. Zhernakova, Rick Jansen, Peter van 't Hof, Patrick Deelen, Irene Nooren, Peter A.C. 't Hoen, Bastiaan T. Heijmans, Matthijs Moed. Data Analysis Group Lude Franke (Co-Chair), Martijn Vermaat, Sasha V. Zhernakova, René Luijk, Marc Jan Bonder, Maarten van Iterson, Patrick Deelen, Freerk van Dijk, Michiel van Galen, Wibowo Arindrarto, Szymon M. Kielbasa, Morris A. Swertz, Erik. W van Zwet, Rick Jansen, Peter-Bram 't Hoen (Co-Chair), Bastiaan T. Heijmans (Co-Chair).

CHARGE-HF consortium: For a full list of CHARGE - Heart Failure Working Group members contributing to this work funding sources, and further acknowledgements, please see PMID 20445134.

CHARGE-SCD consortium: Foram N. Ashar, Christine M. Albert, Christopher Newton-Cheh, Jennifer A. Brody, Martina Muller-Nurasyid, Anna Moes, Angel Mak, Heikki Huikuri, M Juhani Junttila, Philippe Goyette, Sara L. Pulit, Aravinda Chakravarti, Georg Ehret, Josef Coresh, Man Li, Ronald J. Prineas, Catherine O. Johnson, Oscar H. Franco, Pui-Yan Kwok, Thomas Lumley, Florence Dumas, Barbara McKnight, Jerome I. Rotter, Rozenn N. Lemaitre, Susan Heckbert, Christopher J. O'Donnell, Shih-Jen Hwang, Jean-Claude Tardif, Marja-Leena Kortelainen, Martin VanDenBurgh, Andre G Uitterlinden, Albert Hofman, Bruno H. C. Stricker, Thomas Meitinger, Mark Eijgelsheim, John D. Rioux, Ken Rice, Xavier Jouven, Stefan Kaab, Bruce M. Psaty, David S. Siscovick, Dan E. Arking, Nona Sotoodehnia, for the SCD working group of the CHARGE Consortium.

CKDGen consortium: We thank all members of the CKDGen Consortium for the sharing of summary statistics.

Lifelines Cohort Study: Behrooz Z. Alizadeh, H. Marike Boezen, Lude Franke, Pim van der Harst, Gerjan Navis, Marianne Rots, Harold Snieder, Morris Swertz, Bruce H.R. Wolffenbuttel, Cisca Wijmenga.

OFS: We thank the people of the Taimsa, Birkat AI-Moz, Radat Al-Busaidi, Karsha, and Farq villages for their continuous encouragement and participation. We thank the Ministry of Health and the Wali (Governor) of Nizwa District and the H. M. Sultan Qaboos Strategic Research Trust Fund (Grant SR/MED/PHYS/ O4/01) for supporting this work.
The Oman Family Study (OFS) was approved by the Research and Ethics Committee of College of Medicine and Health Sciences at Sultan Qaboos University, Muscat Oman under the title "Hypertension and the metabolic syndrome in Oman intervention clinical trial and mapping of genes" (SR/MED/PHYS/01/01 and SR/MED/PHYS/04/01). All participants fully consented to the study by signing the written consent form or by thumb stamping for those who were illiterate.

N. Sotoodehnia was supported by grants R01HL116747 and R01HL111089.
M. den Hoed was supported by the Swedish research Council (2015-03657) and Swedish Heart-Lung Foundation (20140543).

S.W. van der Laan was funded through grants from the Netherlands CardioVascular Research Initiative ("GENIUS", CVON2011-19), the Interuniversity Cardiology Institute of the Netherlands (ICIN, 09.001), and through the FP7 EU project CVgenes@target (HEALTH-F2-2013-601456)

SUPPLEMENTARY REFERENCES

1. Kumar,P., Henikoff,S., & Ng,P.C. Predicting the effects of coding non-synonymous variants on protein function using the SIFT algorithm. *Nature Protocols* **4**, 1073-1081 (2009).

2. Adzhubei,I.A. *et al.* A method and server for predicting damaging missense mutations. *Nature Methods* **7**, 248-249 (2010).

3. Boyle,A.P. *et al.* Annotation of functional variation in personal genomes using RegulomeDB. *Genome Research* **22**, 1790-1797 (2012).

4. Liu,J.Z. *et al.* A versatile gene-based test for genome-wide association studies. *American Journal of Human Genetics* **87**, 139-145 (2010).

5. Pers,T.H., Dworzynski,P., Thomas,C.E., Lage,K., & Brunak,S. MetaRanker 2.0: a web server for prioritization of genetic variation data. *Nucleic Acids Research* **41**, W104-W108 (2013).

6. Pers,T.H. *et al.* Biological interpretation of genome-wide association studies using predicted gene functions. *Nature Communications* **6**, 5890 (2015).

7. Chen,J., Xu,H., Aronow,B.J., & Jegga,A.G. Improved human disease candidate gene prioritization using mouse phenotype. *BMC Bioinformatics* **16**, 392 (2007).

8. Arena,J. *et al.* Complex I defect in muscle from patients with Huntington's disease. *Annals of Neurology* **43**, 398-400 (1998).

9. Mostafavi,S., Debajyoti,R., Warde-Farley,D., Grouios,C., & Morris,Q. GeneMANIA: a real-time multiple association network integration algorithm for prediciting gene function. *Genome Biology* **9**, S4 (2008).

10. Stewart,A., Huang,J., & Fisher,R.A. RGS proteins in heart brakes on the vagus. *Frontiers in Physiology* **3**, (2012).

11. Posokhova,E., Wydeven,N., Allen,K.L., Wickman,K., & Martemyanov,K.A. RGS6/Gbeta5 complex accelerates IKACh gating kinetics in atrial myocytes and modulates parasympathetic regulation of heart rate. *Circulation Research* **107**, 1350-1354 (2010).

12. Baruscotti,M., Barbuti,A., & Bucchi,A. The cardiac pacemaker current. *Journal of Molecular and Cellular Cardiology* **48**, 55-64 (2010).

13. Baruscotti,M., Bottelli,G., Milanesi,R., DiFrancesco,J.C., & DiFrancesco,D. HCN-related channelopathies. *Pflugers Archiv-European Journal of Physiology* **460**, 405-415 (2010).

14. den Hoed M. *et al.* Identification of heart rate-associated loci and their effects on cardiac conduction and rhythm disorders. *Nature Genetics* **45**, 621-631 (2013).

15. International Consortium for Blood Pressure Genome-Wide Association Studies *et al.* Genetic variants in novel pathways influence blood pressure and cardiovascular disease risk. *Nature* **478**, 103-109 (2011).

16. Smith,N.L. *et al.* Assocation of genome-wide variation with the risk of incident heart failure in adults of European and African ancestry: a prospective meta-analysis from the cohorts for heart and aging research in genomic epidemiolgy (CHARGE) consortium. *Circulation Cardiovascular Genetics* **3**, 256-266 (2010).

17. Deloukas,P. *et al.* Large-scale association analysis identifies new risk loci for coronary artery disease. *Nature Genetics* **45**, 25-33 (2013).

18. Ellinor,P.T. *et al.* Meta-analysis identifies six new susceptibility loci for atrial fibrililation. *Nature Genetics* **44**, 670-675 (2012).

19. Morris,A.P. *et al.* Large-scale association analysis provides insights into the genetic architecture and pathophysiology of type 2 diabetes. *Nature* **44**, 981-990 (2012).

20. Locke,A.E. *et al.* Genetic studies of body mass index yield new insights for obesity biology. *Nature* **518**, 197-206 (2015).

21. Pattaro,C. *et al.* Genetic associations at 53 loci highlight cell types and biological pathways relevant for kidney function. *Nat. Commun.* **7**, 10023 (2016).

22. Teumer,A. *et al.* Genome-wide Association Studies Identify Genetic Loci Associated With Albuminuria in Diabetes. *Diabetes* **65**, 803-817 (2016).

23. Jansen,R. *et al.* Sex differences in the human peripheral blood transcriptome. *BMC Genomics* **15**, 33 (2014).

24. Wright,F.A. *et al.* Heritability and genomics of gene expression in peripheral blood. *Nature Genetics* **46**, 430-437 (2014).

25. Zhernakova,D.V. *et al.* Identification of context-dependent expression quantitative trait loci in whole blood. *Nat. Genet.* **49**, 139-145 (2017).

26. Westra,H.J. *et al.* Systematic identification of trans eQTLs as putative drivers of known disease assocations. *Nature Genetics* **1238-1243**, (2013).

27. Lappalainen,T. *et al.* Transcriptome and genome sequencing uncovers functional variation in humans. *Nature* **501**, 506-511 (2013).

28. Ramasamy,A. *et al.* Genetic variability in the regulation of gene expression in ten regions of the human brain. *Nature Neuroscience* **17**, 1418-1428 (2014).

29. Koopmann,T.T. *et al.* Genome-wide identification of expression quantitative trait loci (eQTLs) in human heart. *PLoS ONE* **9**, e97380 (2014).

30. Bonder,M.J. *et al.* Disease variants alter transcription factor levels and methylation of their binding sites. *Nat. Genet.* **49**, 131-138 (2017).

31. Goedhart,A.D., van der Sluis,S., Houtveen,J.H., Willemsen,G., & de Geus,E.J. Comparison of time and frequency domain measures of RSA in ambulatory recordings. *Psychophysiology* **44**, 203-215 (2007).

32. Grossman,P., van Beek,J., & Wientjes,C. A comparison of three quantification methods for estimation of respiratory sinus arrhythmia. *Psychophysiology* **27**, 702-714 (1990).

33. Wang,X., Thayer,J.F., Treiber,F., & Snieder,H. Ethnic differences and heritability of heart rate variability in African- and European American youth. *The American Journal of Cardiology* **96**, 1166-1172 (2005).

34. Neijts,M. *et al.* Heritability of cardiac vagal control in 24-h heart rate variability recordings: influence of ceiling effects at low heart rates. *Psychophysiology* **51**, 1023-1036 (2014).

35. Goedhart,A.D., Willemsen,G., Houtveen,J.H., Boomsma,D.I., & de Geus,E.J. Comparing low frequency heart rate variability and preejection period: two sides of a different coin. *Psychophysiology* **45**, 1086-1090 (2008).

36. Reyes Del Paso,G.A., Langewitz,W., Mulder,L.J., van Roon,A., & Duschek,S. The utility of low frequency heart rate variability as an index of sympathetic cardiac tone: a review with emphasis on a reanalysis of previous studies. *Psychophysiology* **50**, 477-487 (2013).

37. Monfredi,O. *et al.* Biophysical Characterization of the Underappreciated and Important Relationship Between Heart Rate Variability and Heart Rate. *Hypertension* **64**, 1334-U386 (2014).

38. Vaez,A. *et al.* An in silico post-GWAS analysis of C-reactive proteing loci suggests an important role for interferons. *Circulation: Cardiovascular Genetics* **8**, 487-497 (2015).

39. Berger,I. *et al.* Mitochondrial complex deficiency caused by a deleterious NDUFA11 mutation. *Annals of Neurology* **63**, 405-408 (2008).

40. Finsterer,J. Leigh and Leigh-like syndrome in children and adults. *Pediatric Neurology* **39**, 223-235 (2008).

41. Pfeufer,A. *et al.* Genome-wide association study of PR interval. *Nature Genetics* **42**, 153-159 (2010).

42. Folk,P., Puta,F., & Skruzny,M. Transcriptional coregulator SNW/SKIP: the concealed tie of dissimilar pathways. *Cellular and Molecular Life Sciences* **61**, 629-640 (2004).

43. Xu,C. *et al.* Solution structure of human peptidyl prolyl isomerase-like protein 1 and insights into its interaction with SKIP. *The Journal of Biological Chemistry* **281**, 15900-15908 (2006).

44. Makinen,V.P. *et al.* Integrative genomics reveals novel molecular pathways and gene networks for coronary artery disease. *PLoS Genetics* **10**, e1004502 (2014).

45. Cao,P., Maximov,A., & Sudhof,T.C. Activity-dependent IGF-1 exocytosis is controlled by the Ca(2+)-sensor synaptotagmin-10. *Cell* **145**, 300-311 (2011).

46. Sudhof,T.C. Calcium control of neurotransmitter release. *Cold Spring Harb Perspectives in Biology* **4**, a011353 (2012).

47. Dunant,Y. & Cordeiro,J.M. Presynaptic K(+) channels, vesicular Ca(2+)/H (+) antiport--synaptotagmin, and acetylcholinesterase, three mechanisms cutting short the cholinergic signal at neuromuscular and nerve-electroplaque junctions. *Journal of Molecular Neuroscience* **53**, 377-386 (2014).

48. Oldham,W.M. & Hamm,H.E. Heterotrimeric G protein activation by G-protein-coupled receptors. *Nature Reviews Molecular Cell Biology* **9**, 60-71 (2008).

49. Hossain,M.N., Sakemura,R., Fujii,M., & Ayusawa,D. G-protein gamma subunit GNG11 strongly regulates cellular senescence. *Biochemical and Biophysical Research Communications* **351**, 645-650 (2006).

50. Balcueva,E.A. *et al.* Human G protein gamma(11) and gamma(14) subtypes define a new functional subclass. *Experimental Cell Research* **257**, 310-319 (2000).

51. Yang,J. *et al.* RGS6, a modulator of parasympathetic activation in heart. *Circulation Research* **107**, 1345-1349 (2010).

52. Wydeven,N., Posokhova,E., Xia,Z., Martemyanov,K.A., & Wickman,K. RGS6, but not RGS4, is the dominant regulator of G protein signaling (RGS) modulator of the parasympathetic regulation of mouse heart rate. *The Journal of Biological Chemistry* **289**, 2440-2449 (2014).

53. Milanesi,R., Bucchi,A., & Baruscotti,M. The genetic basis for inherited forms of sinoatrial dysfunction and atrioventricular node dysfunction. *Journal of Interventional Cardiac Electrphysiology* **43**, 121-134 (2015).

54. Milano,A. *et al.* HCN4 mutations in multiple families with bradycardia and left ventricular noncompaction cardiomyopathy. *Journal of the American College of Cardiology* **64**, 745-756 (2014).

55. Wilson,N.H. & Key,B. Neogenin: one receptor, many functions. *International Journal of Biochemistry and Cellular Biology* **39**, 874-878 (2007).

56. Le Douarin,N.M. Embryonic neural chimaeras in the study of brain development. *Trends in Neurosciences* **16**, 64-72 (1993).

57. Vielmetter,J., Kayyem,J.F., Roman,J.M., & Dreyer,W.J. Neogenin, an avian cell surface protein expressed during terminal neuronal differentiation, is closely related to the human tumor suppressor molecule deleted in colorectal cancer. *The Journal of Cell Biology* **127**, 2009-2020 (1994).

58. Slorach,E.M. & Werb,Z. Epithelial morphogenesis: Netrin comes to a sticky and terminal end. *Current Biology* **13**, R491-R493 (2003).
