# Supplementary figures and images for: Erratum: Genetic loci associated with heart rate variability and their effects on cardiac disease risk
Source: Nat Commun. 2017 Aug 2;8:16140. doi: 10.1038/ncomms16140 (PMC5543301; doi:10.1038/ncomms16140)

(a)

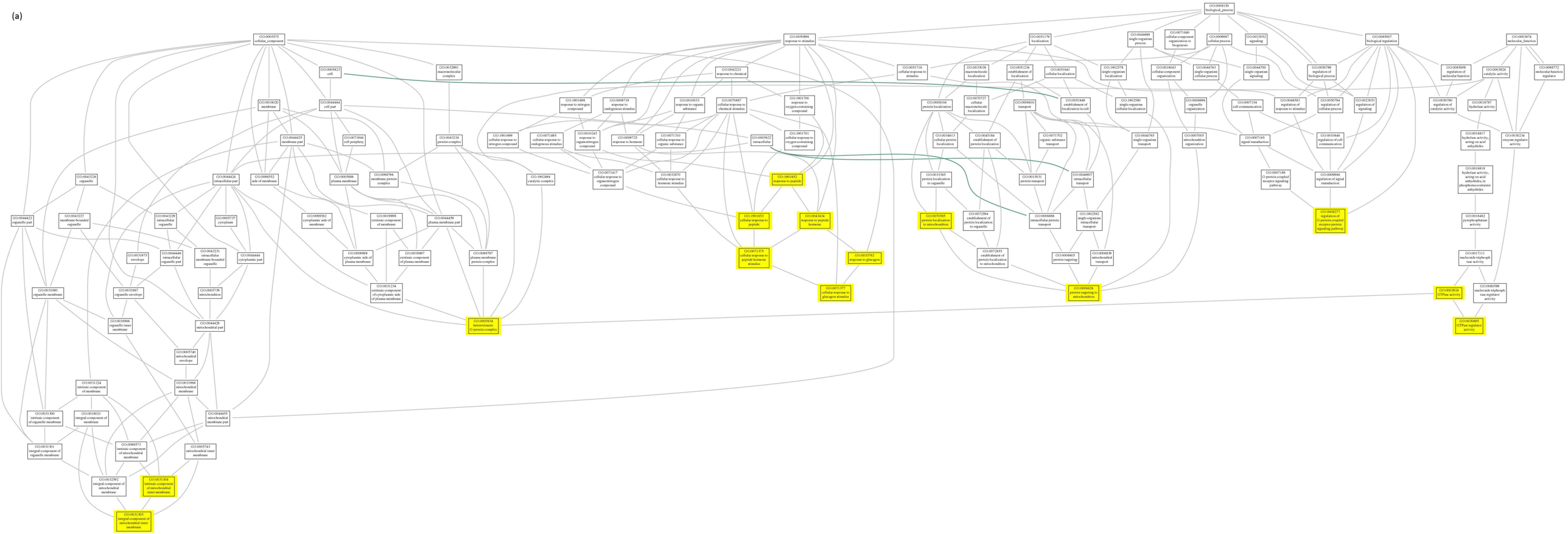

(b)

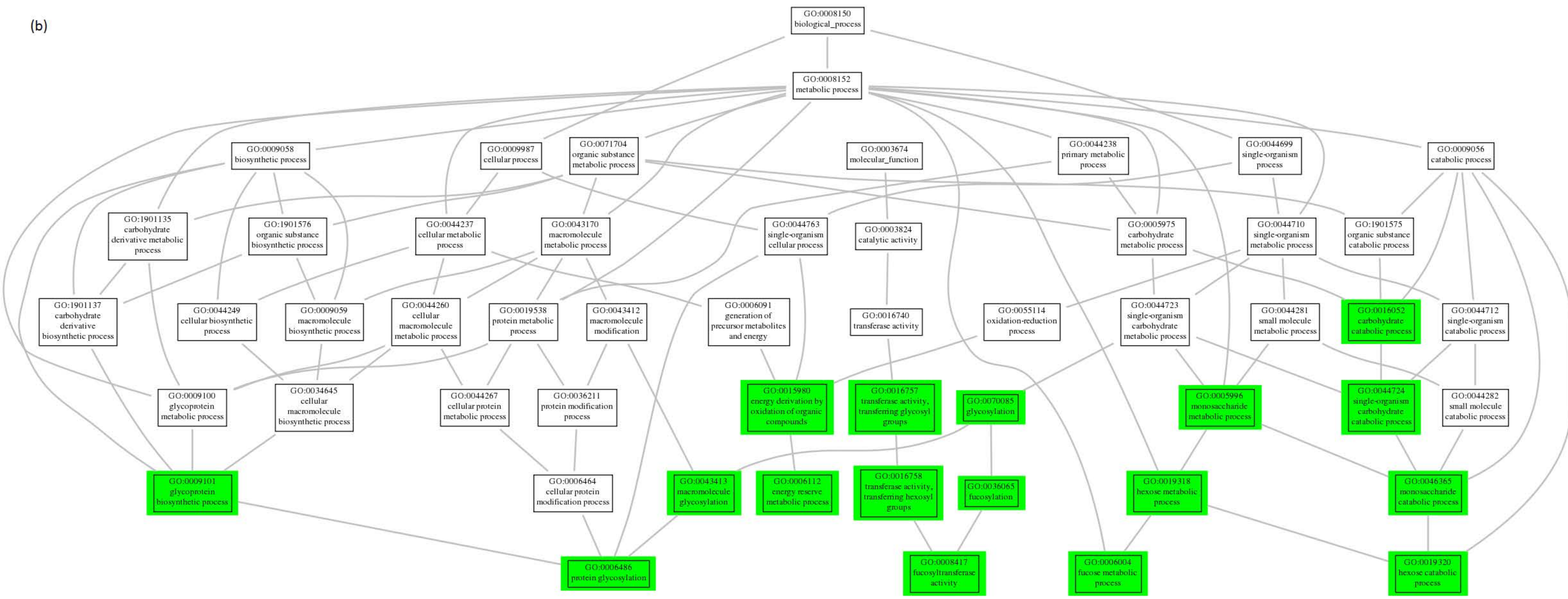

Supplement: Supplementary Information [file ncomms16140-s1.pdf]
